# Design and Characterization of Dual‐Modal Fluorinated BODIPY Probes

**DOI:** 10.1002/cplu.70222

**Published:** 2026-08-13

**Authors:** Nazeeha Ayaz, Marta Rosati, Marco Damoli, Johnny Camilo Perez Rosas, Andrea Pizzi, Pierangelo Metrangolo, Simonetta Orlandi, Marta Penconi, Marco Cavazzini, Francesca Baldelli Bombelli

**Affiliations:** ^1^ Bombelli Laboratory of Supramolecular and Bio‐Nanomaterials (SupraBioNano Lab) Department of Chemistry, Materials and Chemical Engineering, “Giulio Natta” Politecnico di Milano Milan Italy; ^2^ Institute of Chemical Sciences and Technologies “Giulio Natta” (CNR‐SCITEC) National Research Council (CNR) Milan Italy

**Keywords:** ^19^F‐NMR, boron–dipyrromethene, fluorine, poly(lactic‐co‐glycolic acid) nanoparticles

## Abstract

Multimodal imaging can improve the detection of molecular and pathophysiological changes by combining complementary readouts within a single probe. Here we report the synthesis and characterization of a fluorinated BODIPY dye (**F** **_54_** **‐BODIPY**) designed as dual fluorescence/^19^F‐MRI imaging agent. The molecular design replaces the native BF_2_ unit with multibranched perfluoro‐tert‐butoxy chains appended at the boron atom via B—O hexyloxy linkages, providing 54 magnetically equivalent ^19^F nuclei per molecule. Additionally, 2,6‐dimethylphenyl groups are introduced at 2,6‐positions of **F** **_54_** **‐BODIPY**, and (E)‐4‐methylstyryl residues at the 3,5‐positions extend emission into the far‐red region of visible spectrum. This probe was structurally characterized by NMR spectroscopy, and its photophysical properties were evaluated by UV–Vis absorption, fluorescence spectroscopy, and fluorescence quantum yield (*Φ* *_f_*) measurements. Subsequently **F** **_54_** **‐BODIPY** was encapsulated in poly(lactic‐co‐glycolic acid) (PLGA) nanoparticles (NPs) for dispersion in aqueous media. Co‐encapsulation of a second fluorinated phase, namely perfluoro‐15‐crown‐5 ether (PFCE), significantly modified fluorescence properties and introduced a second ^19^F‐NMR signal, yielding a bimodal nanosystem with dual ^19^F‐NMR signals and red fluorescence emission for combined fluorescence/^19^F‐MRI bioimaging.

## Graphical abstract

Here we show a fluorinated BODIPY dye that combine ^19^F traceability with photoluminescence, properly designed to reduce aggregation‐induced quenching and to adjust the packing in condensed environments. The molecule can be encapsulated in Pluronic F68‐stabilized poly(lactic‐co‐glycolic acid) nanoparticles. We demonstrate that the addition of perfluoro‐15‐crown‐5 ether resulted in an improvement of nanoformulation imaging performance.

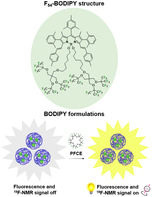

## 1 Introduction

Recent advances in biomedical imaging have increasingly focused on the development of multimodal imaging strategies to enable more accurate detection of molecular, pathophysiological, and anatomical alterations. While individual imaging modalities offer distinct advantages, each also presents inherent limitations that can be overcome by combining complementary techniques. In this framework, the development of a single molecular probe capable of supporting dual imaging modalities is highly desirable, as it ensures co‐localized signal generation, unified pharmacokinetics, and simplified probe administration [1, 2].

Boron–dipyrromethene (BODIPY) dyes constitute a well‐established class of fluorophores distinguished by their high molar absorption coefficients, favorable fluorescence quantum yields, and exceptional chemical and photostability. The integration of magnetic resonance imaging (MRI) capability into BODIPY‐based probes represents an appealing strategy to expand their functionality beyond optical imaging. In particular, the incorporation of fluorine atoms into the BODIPY scaffold offers the opportunity to enable detection by ^19^F‐NMR/MRI, techniques that provide quantitative, background‐free imaging due to the absence of endogenous fluorine in soft tissues. The ^19^F‐NMR signal is proportional to the number of fluorine nuclei, allowing accurate probe quantification, while wide ^19^F chemical shift range enables unambiguous identification of fluorinated compounds [3, 6].

Combining excellent optical properties of BODIPY dyes with the specificity and quantitative potential of ^19^F‐NMR/MRI within a single molecular platform would therefore yield a powerful dual‐modal imaging probe capable of delivering complementary anatomical, molecular, and functional information in vivo [7, 8]. In support of this concept, it has been reported that self‐assembled polyethylene glycol–BODIPY amphiphiles functionalized with 54 fluorine atoms formed theranostic emulsions in presence of perfluorohexane (PFH), which were detectable by fluorescence (FL), photoacoustic (PA), and ^19^F‐MRI, while also exhibiting photodynamic therapeutic activity [9]. Additionally, redox‐responsive fluorinated aza‐BODIPY emulsions have been employed for phototherapeutic treatment of lung cancer, while being detectable by FL, PA, ^19^F‐MRI, and photothermal imaging. This multimodal imaging capability enabled accurate tumor localization and real‐time image‐guided therapy, thereby enhancing treatment precision and therapeutic efficacy [10].

In this framework, we previously reported the synthesis and spectroscopic characterization of a series of fluoroalkoxy‐substituted BODIPY derivatives containing either 27 or 54 magnetically equivalent fluorine atoms per molecule, using multibranched fluorinated chains inspired by the performing ^19^F‐MRI probe PERFECTA [11, 14]. Among these compounds, those functionalized with styryl groups at the 3,5‐positions proved particularly promising as bioimaging probes, as they exhibited far‐red emission spectra, enabling improved tissue penetration and enhanced suitability for in vivo imaging applications [15]. We also observed that the incorporation of two iodine atoms in their structure generally reduced their fluorescence quantum yield (*Φ* *_f_*), as consequence of the intramolecular heavy‐atom effect, whereby iodine enhances spin–orbit coupling and accelerates intersystem crossing (ISC) between lowest singlet excited state *S*_1_ and the triplet manifold *T*_1_ at the expense of radiative emission. Conversely, the presence of the two bulky branched fluorinated substituents in the derivative—containing a total of 54 fluorine atoms—significantly increased the separation between the BODIPY cores, reducing π–π stacking interactions and ultimately led to a higher *Φ*_*f*_ for this compound [15, 16]. For these reasons, this compound, **F**_**54**_ **I‐BODIPY,** was identified as the most promising candidate for bimodal imaging applications, but to further improve its features we synthesized an analog in which the two iodine atoms were replaced with 2,6‐dimethylphenyl groups to further explore structure–property relationships. However, these multimodal probes are not inherently dispersible in aqueous media and therefore required appropriate formulation to enable their use in biomedical applications.

**FIGURE 1:**
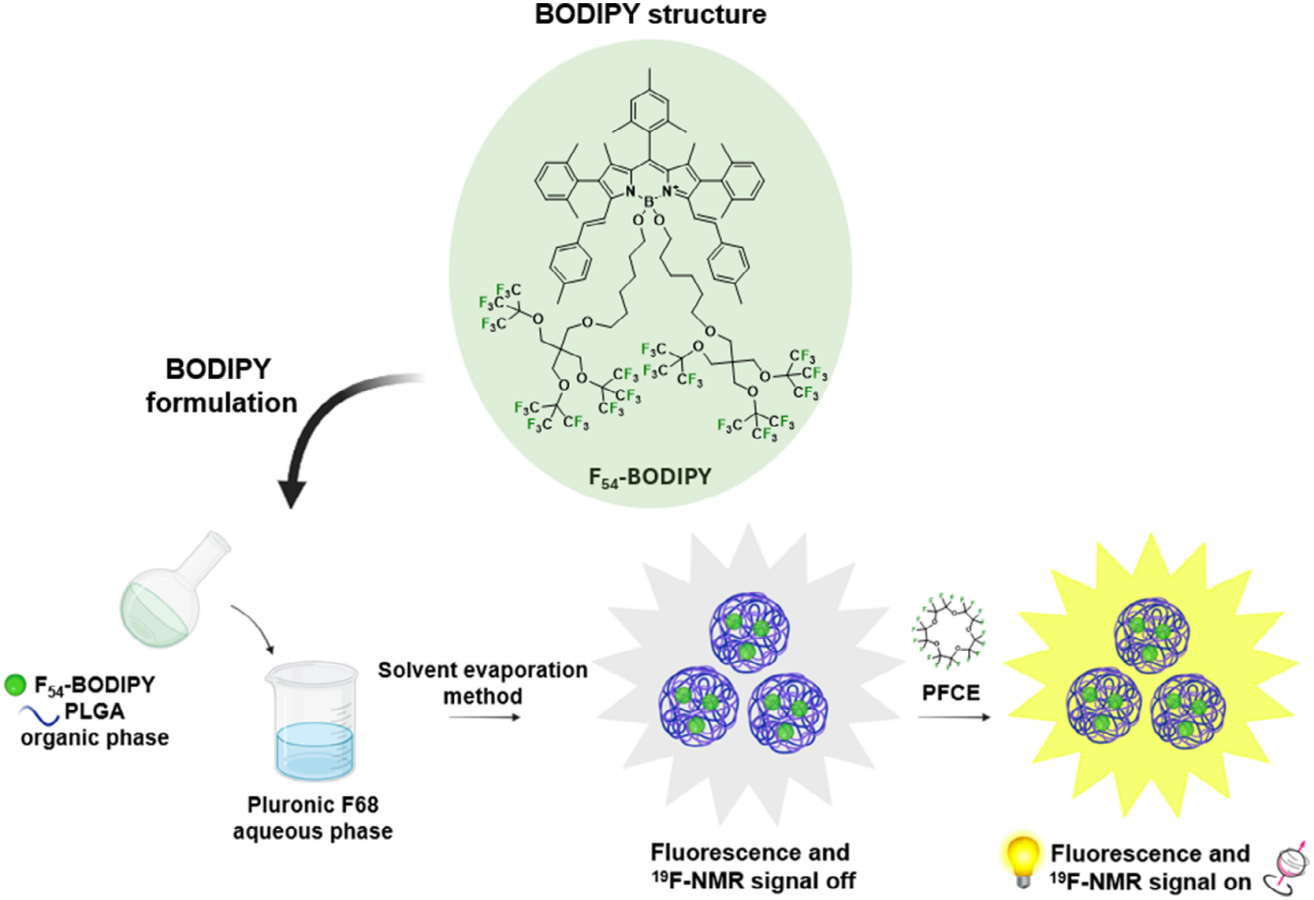
Upper section presents the chemical structure of fluorinated **F**_**54**_ **‐BODIPY** dye; lower section features a schematic illustration of BODIPY formulation in polymeric nanoparticles, highlighting the activation effect on ^19^F‐NMR and fluorescence upon the addition of PFCE to the nanoparticles.

In this work, we report the synthesis and characterization of a new iodine‐free analog (**F**_**54**_ **‐BODIPY**), along with the development of poly(lactic‐co‐glycolic acid) (PLGA) nanoparticle (NP) formulations designed to disperse these F_54_‐BODIPYs (**F**_**54**_ **‐BODIPY** and **F**_**54**_ **I‐BODIPY**) derivatives in aqueous solutions and enable their application as bimodal imaging probes for ^19^F‐NMR/MRI and FL (Figure 1). The resulting NP formulations were systematically optimized in terms of size, colloidal stability, and spectroscopic properties to meet the requirements of biomedical use and compared in terms of imaging features. Notably, incorporation of a fluorinated phase such as perfluoro‐15‐crown‐5 ether (PFCE) led to a marked enhancement of the imaging performance for both derivatives. The iodine‐free derivative exhibited the most favorable overall profile, combining increased fluorescence emission with a clear and well‐resolved dual ^19^F‐NMR signal. This resulted in a multimodal nanosystem characterized by two distinct ^19^F‐NMR resonances alongside far‐red fluorescence emission, making it the most promising candidate for further development (Figure 1).

## 2 Results and Discussion

### 2.1 Synthesis and Physicochemical Characterization of F_54_‐BODIPY

**SCHEME 1:**
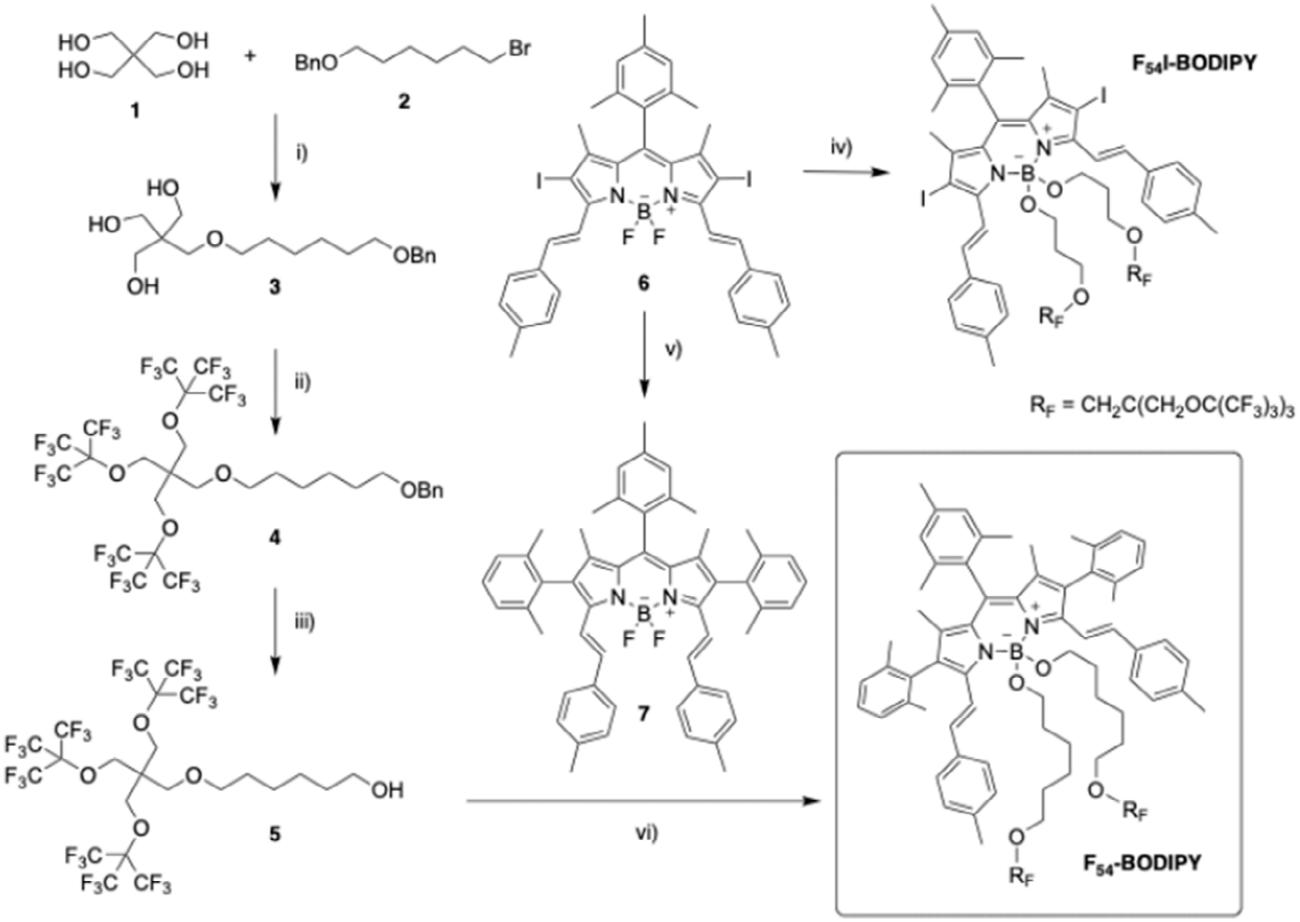
(i) KOH, DMSO, rt, 38%; (ii) nonafluoro‐tert‐butyl alcohol, PPh_3_, DIAD, Et_2_O, 0 °C → reflux, 40 h, 74%; (iii) H_2_, 10% Pd/C, EtOAc, *p* = 1 bar, rt, 92%; (iv) HO(CH_2_)_3_OR_F_, AlCl_3_, THF, 60 °C, 5 min, 52% [15]; (v) (2,6‐dimethylphenyl)boronic acid, Pd(OAc)_2_, S‐PHOS, K_3_PO_4_, CPME, *T* = 115 °C, 3 h, 89%; and (vi) **5**, AlCl_3_, CH_2_Cl_2_/THF, rt, 18 h, 81%.

The synthesis of **F**_**54**_ **‐BODIPY** is outlined in Scheme 1. Compared with the BODIPYs previously prepared by us [15], **F**_**54**_ **‐BODIPY** exhibits two distinct features. First, the iodine atoms at the C2 and C6 positions of the BODIPY core were replaced with sterically bulky 2,6‐dimethylphenyl substituents, with the aim of interfere with, and possibly suppressing, two independent quenching mechanisms at different levels of molecular organization. The substitution of iodine atoms with aryl groups prevents ISC pathway, which is the main cause of the reduced fluorescence quantum yield observed for iodine‐BODIPY even in dilute solutions. At the supramolecular level, however, intermolecular π‐π stacking is possible between BODIPY cores within a condensed environment, such as the hydrophobic core of a PLGA NP, which opens up additional non‐radiative decay pathways/channels that further dampen fluorescence [17, 20]. The choice of sterically hindering aryl substituents in **F**_**54**_ **‐BODIPY** is based on the hypothesis that they would inhibit coplanar aggregation as much as possible from multiple directions: The orthogonally oriented *meso*‐mesityl group shields one face of the boraindacene π‐system [17, 21], while the 2,6‐dimethylphenyl substituents at C2, C6 positions provide lateral steric hindrance that further disrupts close‐range interactions [18, 19, 22]. Furthermore, the presence of methyl groups in the ortho positions limits the internal rotation of the aryl rings relative to the boraindacene plane, allowing for the suppression of nonradiative relaxation phenomena and thus enhancing *Φ*_f_ [23].

Second, the alkyl spacer connecting the fluorinated residue to the optically active BODIPY core was lengthened to six carbon atoms to better decouple the two domains. Extending the spacer length reduces the through‐bond and through‐space influence of the strongly electron‐withdrawing fluorinated block on the chromophore. Moreover, the separation of the two domains should help maintain segmental motion of the fluorinated groups under condensed‐phase conditions, such as high local concentration or nanoparticle encapsulation. This is beneficial for minimizing aggregation‐related quenching and for maintaining a detectable, less broadened ^19^F‐NMR/MRI signal.

Initial attempts to access alcohol **3** via literature procedures for mono‐derivatization of pentaerythritol proved poorly reproducible. Although the protected intermediate (1‐methyl‐2,6,7‐trioxabicyclo [2.2.2]octan‐4‐yl)methanol could be obtained with literature‐consistent characteristics, subsequent alkylation steps consistently gave complex mixtures and delivered triol **3** in low overall yield (typically <40%) with demanding purification. For these reasons, we pursued the more direct route shown in Scheme 1, consisting of the direct alkylation of pentaerythritol **1** with bromohexyl derivative **2**. In this way triol **3** was obtained at 38% yield comparable to the previous synthetic route, but in a single step with obvious improvement in terms of chemistry economy. Reaction with an excess of nonafluoro‐tert‐butyl alcohol under Mitsunobu conditions temperature provided the fluorinated alcohol **4** in 74% yield. Subsequent hydrogenolysis at atmospheric pressure and room temperature yielded alcohol **5** in an overall yield of 25% for the three synthetic steps. In parallel, the diiodo BODIPY **6** underwent Suzuki–Miyaura cross‐coupling with (2,6‐dimethylphenyl)boronic acid in the presence of palladium acetate and the Buchwald‐type ligand S‐Phos, providing BODIPY **7** in 89% yield. Boron functionalization was then carried out by treatment of **7** with alcohol **5** in the presence of AlCl_3_ in a CH_2_Cl_2_/THF mixture (Scheme 1). This Lewis‐acid‐mediated approach provides a straightforward and modular route to incorporate multibranched fluorinated substituents at the boron center, enabling high fluorine loading while retaining the optical features of the BODIPY chromophore.

**FIGURE 2:**
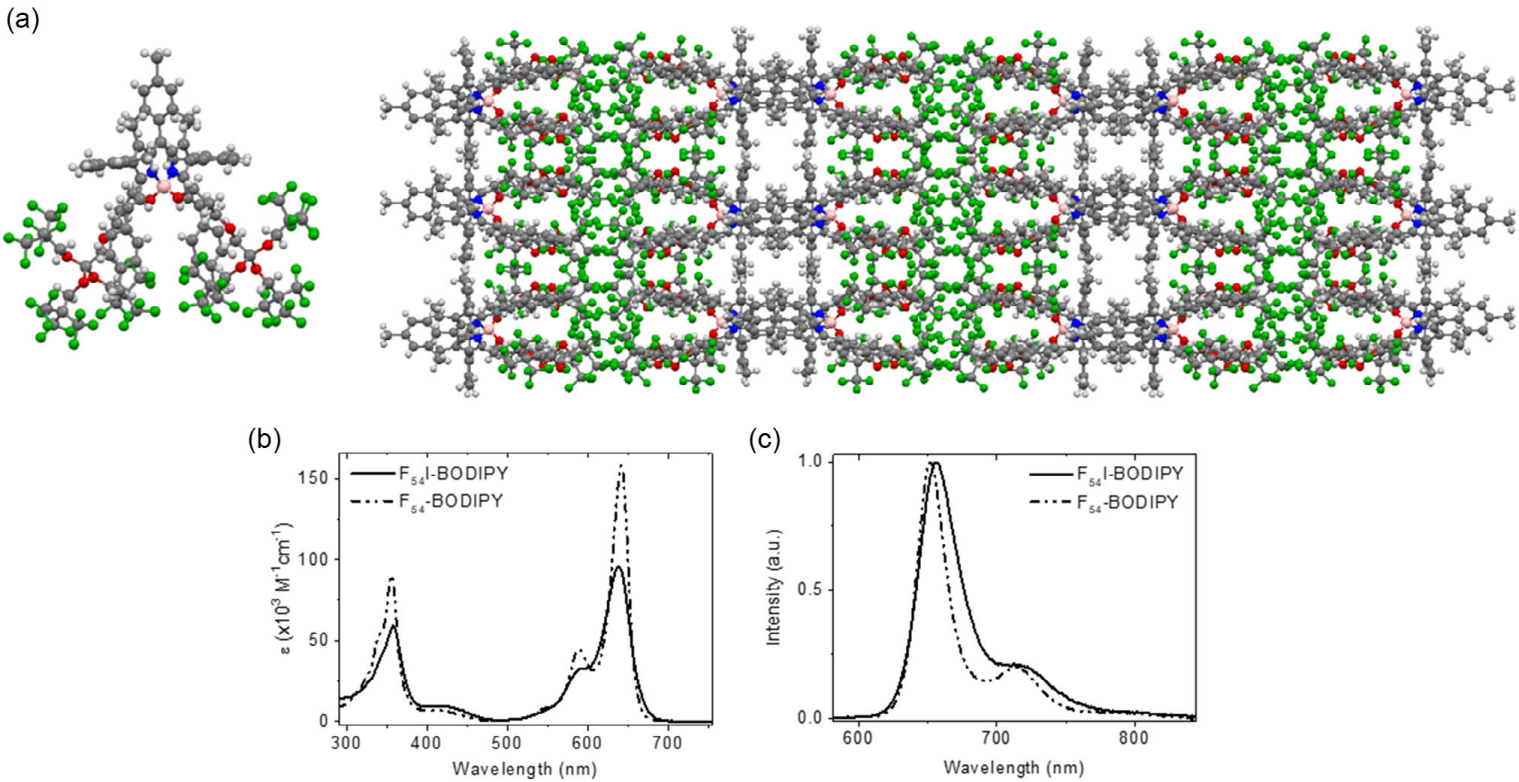
(a) Crystal structures of **F**_**54**_ **‐(CH**_**2**_ **)**_**3**_ **BODIPY**, showing asymmetric units; view of the crystal packing of **F**_**54**_ **‐(CH**_**2**_ **)**_**3**_ **BODIPY** along *c* axis, displaying segregation of hydrocarbon and fluorocarbon regions. Color code: carbon (dark gray), nitrogen (blue), oxygen (red), boron (pink), fluorine (green), hydrogen (light gray). (b) UV–vis absorption spectra and (c) normalized photoluminescence spectra of **F**_**54**_ **I‐BODIPY** and **F**_**54**_ **‐BODIPY** dyes in EtOAc.

Despite several attempts, good quality single crystals of **F**_**54**_ **‐BODIPY** suitable for structure determination could not be obtained. Nevertheless, the best crystals were examined, allowing the determination of a reasonable unit cell and space group (see Supporting Information). Interestingly, this preliminary structural information enabled a qualitative comparison with the crystal structure of an analogous dye, **F**_**54**_ **‐(CH**_**2**_ **)**_**3**_ **BODIPY** (Figure S1), in which the six‐carbon spacer group is replaced by one of three CH_2_ groups. In this regard, **F**_**54**_ **‐(CH**_**2**_ **)**_**3**_ **BODIPY** gave single crystals of better quality for which was possible to resolve the structure that showed a monoclinic C2/*c* system as observed for **F**_**54**_ **‐BODIPY** (see Supporting Information). For this reason, we hypothesized with a certain degree of confidence that the crystal structure described below for **F**_**54**_ **‐(CH**_**2**_ **)**_**3**_ **BODIPY** could serve as reliable model for the crystal packing of **F**_**54**_ **‐BODIPY.** The substituents in the lattice are orthogonal to the boradiazaindacene plane, and fluoroalkoxy chains fold along the same direction of the BODIPY core (see Figure 2a). The high fluorine content promotes pronounced segregation between fluorocarbon and hydrocarbon regions, with adjacent BODIPY molecules adopting antiparallel arrangement in order to maximize packing efficiency. Differently from the previously reported **F**_**54**_ **I‐BODIPY** structure [15], where partial overlap between π systems was present, as assumed in the BODIPY's design, **F**_**54**_ **‐(CH**_**2**_ **)**_**3**_ **BODIPY** lacks π‐π stacking. Overall, main interactions stabilizing solid‐state organization, are weak F···F contacts and C—H···F hydrogen bonds involving the —CF_3_ groups and the aromatic moieties of the BODIPY core.

The absorption and emission spectra of **F**_**54**_ **I‐BODIPY** and **F**_**54**_ **‐BODIPY** dissolved in ethyl acetate (EtOAc), along with their molar extinction coefficients (*ε*_0_), quantum yields (*Φ*_f_), and lifetimes (*τ*), are reported in Figure 2 and Table 1. The results show that the substitution of iodine atoms with 2,6‐dimethylphenyl groups on the BODIPY core does not significantly alter the profile of absorption and emission spectra. In particular, the absorption spectra feature a dominant band at 638 and 642 nm, for **F**_**54**_ **I‐BODIPY** and **F**_**54**_ **‐BODIPY**, respectively, attributed to the HOMO→LUMO (S_0_→S_1_) electronic transition, with a less intense peak at about 595 nm, assigned to the 0–1 vibrational progression. Moreover, a weaker band is present at around 360 nm, associated with the S_0_→S_2_ transition, consistent with BODIPY optical features. Photoluminescence spectra well mirror the lower energy absorption band, with maxima at 656 and 652 nm for **F**_**54**_ **I‐BODIPY** and **F**_**54**_ **‐BODIPY**, respectively, and a vibrational progression above 700 nm. Moreover, higher extinction coefficient and quantum yield were observed for the non‐iodinated compound, as expected.

**TABLE 1:** Photophysical properties of the dyes in EtOAc.

	*λ*_abs_, nm (*ε*, M^−1^ cm^−1^)	*λ*_em_, nm	*Φ*_f_	*τ*, ns
**F**_**54**_ **I‐BODIPY**	357 (59027), 591 (32646), 638 (95630)	656	0.20	1.72
**F**_**54**_ **‐BODIPY**	355 (90398), 589 (44450), 642 (158072)	652	0.77	4.70

### 2.2 Physicochemical Characterization of Biocompatible PLGA NPs Loaded With the Fluorinated BODIPYs

**FIGURE 3:**
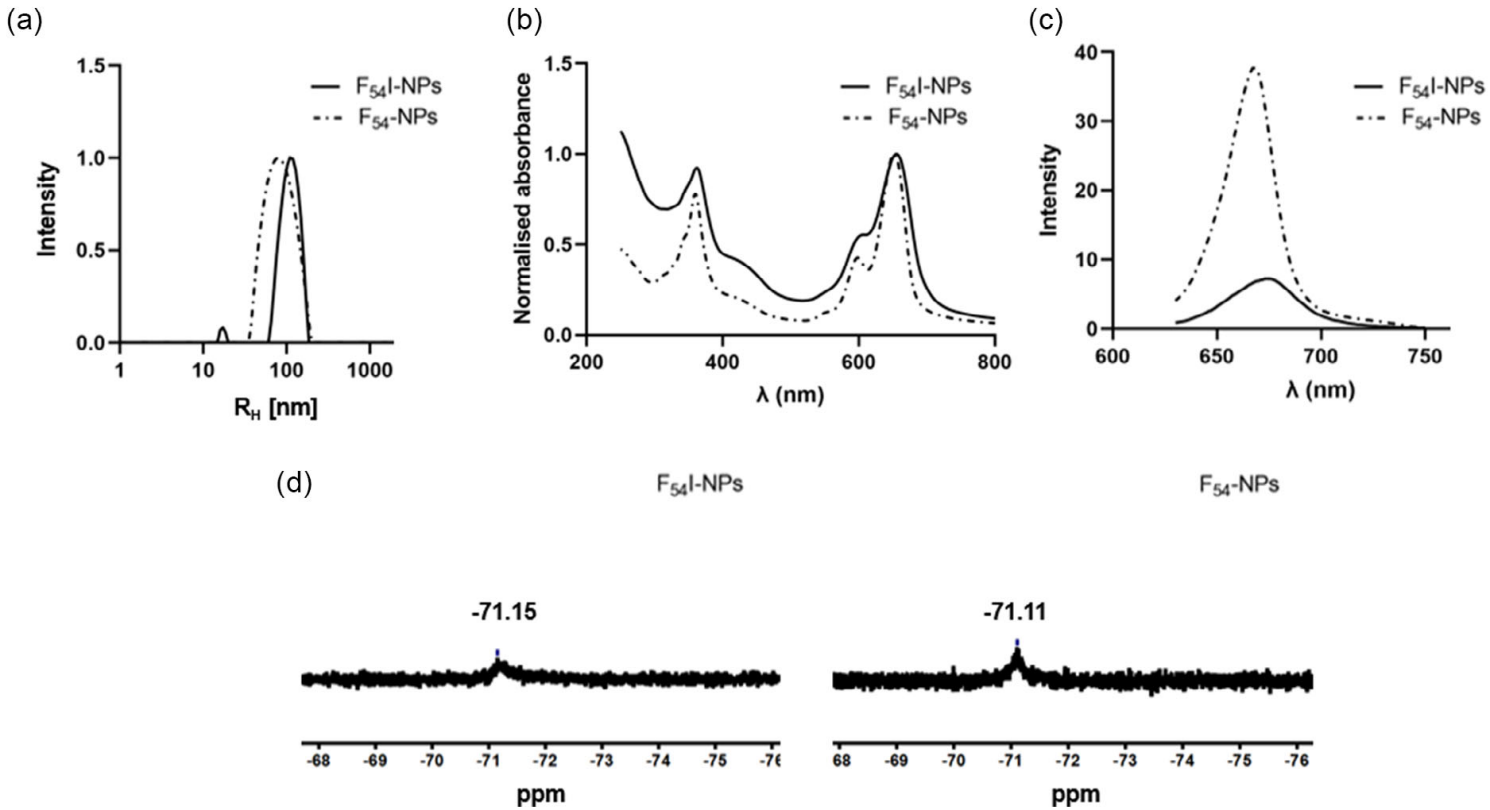
Spectroscopic properties of NPs formulations encapsulating **F**_**54**_ **I‐BODIPY** (**F**_**54**_ **I‐NPs**) and **F**_**54**_ **‐BODIPY** (**F**_**54**_ **‐NPs**), respectively: (a) DLS intensity‐weighted average size distributions (at *θ *= 90°) for NPs formulations encapsulating; (b) UV–vis absorption spectra; (c) photoluminescence spectra at *λ*(exc) = 620 nm; and (d) ^19^F‐NMR spectra.

The fluorinated dyes **F**_**54**_ **‐BODIPY** and **F**_**54**_ **I‐BODIPY** were successfully dispersed in aqueous media through encapsulation within PLGA nanoparticles (NPs), stabilized using the FDA‐approved block copolymer surfactant Pluronic F68, following the protocol described in Section 4.2. The resulting NP dispersions **F**_**54**_ **I‐NPs** and **F**_**54**_ **‐NPs** were characterized by DLS and zeta‐potential measurements. Both formulations yielded colloidally stable nanoparticles with comparable hydrodynamic diameters, polydispersity indices (PDI), and surface charges, as summarized in Table 2 and shown in Figure 3a. The negative zeta potential values (approximately −28 mV) are attributed to the deprotonated terminal carboxylic acid groups of the PLGA chains present at the NP surface.

**TABLE 2:** Hydrodynamic size and zeta‐potential data of PLGA NPs loaded with F‐BODIPYs in aqueous solutions.

Nps		F‐BODIPY	PFCE/BODIPY (molar ratio)	*R* *_H_*, nm	PDI	Zeta‐potential, mV
**F**_**54**_ **I‐NPs**		**F**_**54**_ **I‐BODIPY**	—	78 ± 8	0.20 ± 0.05	−28 ± 8
**F**_**54**_ **‐NPs**		**F**_**54**_ **‐BODIPY**	—	71 ± 7	0.20 ± 0.04	−28 ± 10
**F**_**54**_ **IP‐NPs**		**F**_**54**_ **I‐BODIPY**	9	90 ± 10	0.30 ± 0.03	−23 ± 7
**F**_**54**_ **P‐NPs**		**F**_**54**_ **‐BODIPY**	9	94 ± 8	0.20 ± 0.03	−28 ± 6

The presence of the nonsoluble BODIPY probes embedded in PLGA NPs is confirmed by UV–vis absorption measurements (Figure 3b). Fluorescence emission spectra were recorded upon excitation at 620 nm (Figure 3c) and revealed a single emission maximum at nearly identical wavelengths for both NP formulations. As expected, the fluorescence intensity was higher for the non‐iodinated BODIPY, consistent with the known quenching effect of iodine atoms [24]. Nevertheless, the fluorescence quantum yield significantly decreases from dilute solution (*Φ* *_f_* = 77.0 % for **F**_**54**_ **‐BODIPY**) to nanoconfined environment (*Φ* *_f_* = 18% for **F**_**54**_ **‐NPs**), likely due to aggregation effect within NPs following encapsulation [25]. This hypothesis was further supported by ^19^F‐NMR measurements of the two formulations, which displayed weak and broadened signals centered at approximately −71.1 ppm (Figure 3d). The poor ^19^F‐NMR response is attributed to restricted molecular mobility arising from tight packing and π–π stacking interactions between the BODIPY cores, which limit the motion of the fluorinated chains. Notably, the ^19^F‐NMR signal of **F**_**54**_ **‐NPs** was slightly sharper and exhibited lower background noise compared to that of the iodinated analog **F**_**54**_ **I‐NPs**. However, overall, both F‐BODIPY derivatives encapsulated in PLGA NPs showed decreased fluorescence emission and weak ^19^F‐NMR signals, thereby constraining their effectiveness as standalone imaging agents.

### 2.3 PFCE‐Assisted Signal Activation of F‐BODIPYs in NP Formulations

One of the primary factors underlying the poor imaging performance of **F**_**54**_ **I‐NPs** and **F**_**54**_ **‐NPs** is likely the dense packing of the F‐BODIPY molecules within the confined core of the PLGA NPs. Reduced intermolecular distances can promote aggregation‐induced quenching, leading to diminished fluorescence emission, while simultaneously restricting the mobility of the fluorinated chains and resulting in a weak ^19^F‐NMR response. A potential strategy to enhance the imaging performance of these probes involves the incorporation of a secondary fluorinated phase within the NP, which could promote a higher affinity of F‐BODIPY to the NP core, increasing its dispersion in the aqueous solution and modifying its packing constrains. Among the possible fluorinated phases, we selected perfluoro‐15‐crown‐5‐ether (PFCE), a well‐established ^19^F‐MRI probe, already encapsulated in PLGA NPs [26], characterized by a distinct chemical shift (−91.8 ppm) from that of F‐BODIPY derivatives, facilitating unambiguous spectral discrimination and interpretation and providing potentially two distinct signals in ^19^F‐MRI.

PLGA NPs containing PFCE were prepared at the same F‐BODIPY concentration as those described in 4.2, using an F‐BODIPY/PFCE ratio of 1:9. The incorporation of PFCE into the formulation led to the formation of NPs with a larger hydrodynamic diameter, consistent with effective encapsulation of PFCE within the NP core. PFCE‐loaded **F**_**54**_ **‐BODIPY** NPs (**F**_**54**_ **P‐NPs**) exhibited PDI comparable to those of the corresponding formulations without PFCE, whereas PFCE‐loaded **F**_**54**_ **I‐BODIPY** NPs (**F**_**54**_ **IP‐NPs**) displayed increased polydispersity, with a PDI of approximately 0.3 (Table 2). The zeta potential remained essentially unchanged across formulations, supporting the conclusion that PFCE did not affect NP surface properties.

**FIGURE 4:**
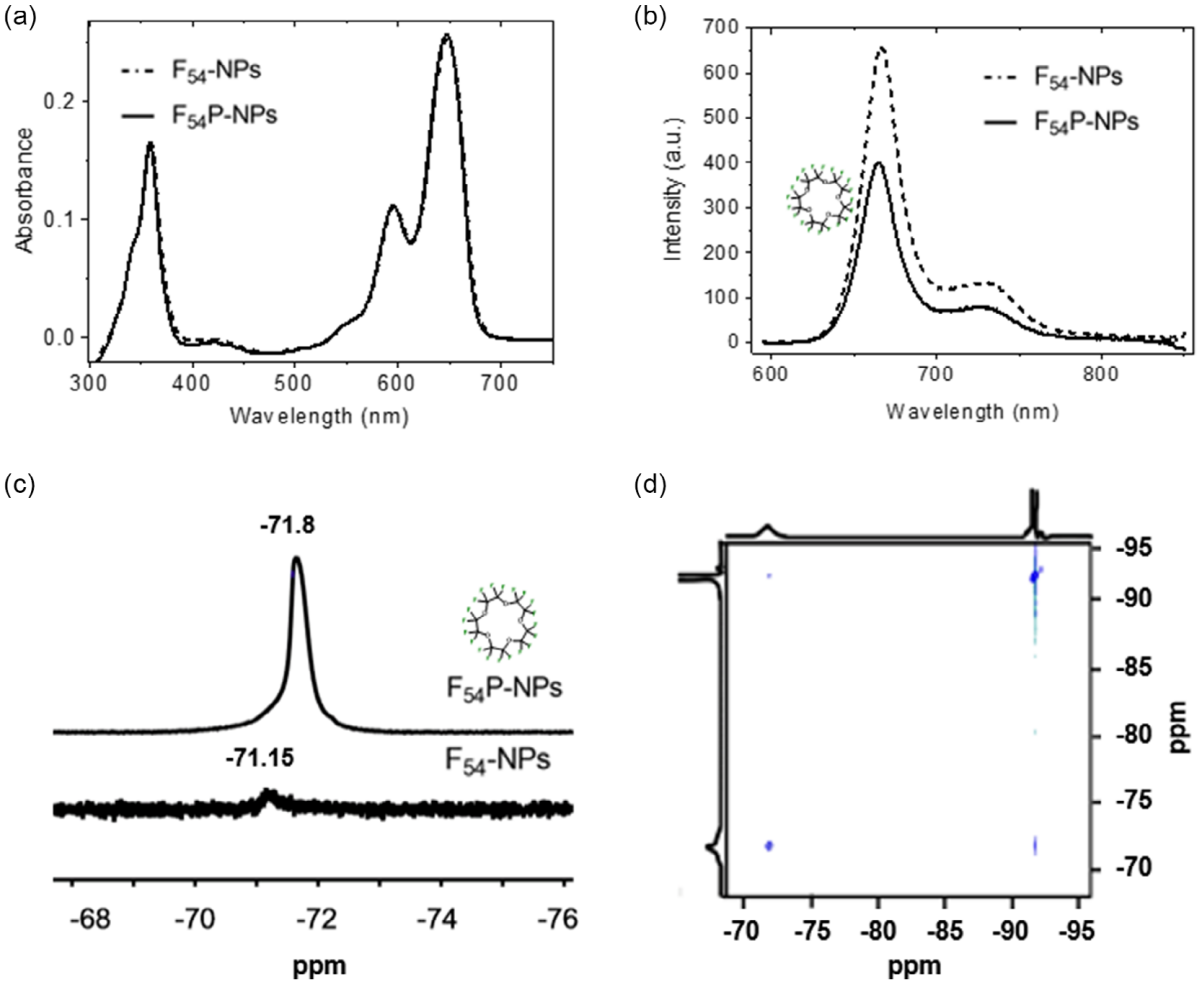
(a) UV‐vis absorption spectra of **F_54_‐NPs** and **F_54_P‐NPs** and (b) photoluminescence spectra at *λ*(exc) = 620 nm of **F_54_‐NPs** and **F_54_P‐NPs**; spectra were acquired under dilution conditions of the stock suspensions (120‐fold for **F_54_IP‐NPs**, 400‐fold for **F_54_P‐NPs**) to ensure a value of *A* < 0.1 at the excitation wavelength. Dilution factors are reported for experimental completeness and are not directly comparable as a measure of chromophore content since the starting concentrations of the stock suspensions were not normalized (c) ^19^F‐NMR spectrum of the samples and (d) ^19^F‐^19^F NOESY spectrum of **F_54_P‐NPs**.

Although the overall NP morphology was not significantly altered by PFCE encapsulation (see Figure S2 ), the spectroscopic behavior was markedly influenced by the presence of the fluorinated phase. In fact, **F**_**54**_ **IP‐NPs** exhibited a pronounced bathochromic shift of the main absorption band. A similar shift was observed in the emission spectrum, with a limited enhancement in fluorescence intensity (Figure S5). In contrast, no red shift was detected for **F**_**54**_ **P‐NPs**. Instead, at the same probe concentration (as determined by UV‐Vis absorption) a reduced fluorescence emission intensity was observed in the presence of PFCE, accompanied by a decrease in the fluorescence quantum yield (*Φ*_*f*_  = 18% for **F**_**54**_ **‐NPs** vs. *Φ*_*f*_  = 10% for **F**_**54**_ **P‐NPs**). Notably, PFCE markedly enhanced the encapsulation efficiency of the probe in **F54P‐NPs**, enabling these NPs to achieve the same absorbance as **F54‐NPs** despite being diluted fourfold (Figure 4a). These results suggest that the presence of PFCE within the NPs alters the organization of the F‐BODIPY molecules in the NPs core, promoting interactions that, at the cost of a reduction in fluorescence, are nevertheless advantageous for the development of a multimodal probe, as clearly shown by ^19^F‐NMR experiments performed on **F**_**54**_ **IP‐NPs** and **F**_**54**_ **P‐NPs**. Indeed, distinct signals corresponding to both PFCE and F‐BODIPYs were clearly detected (Figure S3). Notably, the spectral features in the −71 ppm region differed between the two formulations: The **F**_**54**_ **IP‐NPs** displayed a clear peak splitting, with a dominant resonance at −71.8 ppm accompanied by a distinct secondary peak at −71.2 ppm (Figure S3). In contrast, the **F**_**54**_ **P‐NPs** spectrum showed a predominantly single resonance centered at −71.8 ppm (Figure 4c), characterized by a broadened and asymmetric base extending toward −71.2 ppm rather than a fully resolved split peak. The peak at −71.2 ppm could be related to a possible residual component of packed BODIPYs, while the peak at −71.8 ppm could be attributed to the molecules interacting with PFCE (Figures 4c and S3). To evaluate closeness and interactions between PFCE and F‐BODIPYs in **F**_**54**_ **P‐NPs**, ^19^F‐^19^F NOESY measurements were performed on these NPs in aqueous solution.^19^F Nuclei that are close to each other (<6 Å) are detected by NOESY due to cross‐space magnetization transfer between the nuclei. A nuclear Overhauser effect was observed between PFCE and F‐BODIPY ^19^F, suggesting that these nuclei were close within the NPs (Figure 4d).

## 3 Conclusions

In conclusion, we developed a non‐iodinated F‐BODIPY derivative **F**_**54**_ **‐BODIPY** designed to reduce packing constraints and improve imaging performance upon encapsulation in PLGA NPs. While initial nanoformulations of F‐BODIPYs exhibited limited fluorescence and weak ^19^F‐NMR signals, these shortcomings were attributed to dense molecular packing within the NP core, leading to aggregation‐induced quenching and restricted fluorine mobility. The incorporation of a fluorinated phase, PFCE, effectively overcame these limitations by improving molecular mobility and restoring ^19^F detectability. ^19^F‐^19^F NOESY experiment confirmed close spatial interactions between PFCE and F‐BODIPY within the NP core, supporting a “dissolution‐like” effect that reduces π–π stacking. Although PFCE co‐encapsulation resulted in a decrease in the fluorescence quantum yield of the formulation, this optimized system allows the F‐BODIPY derivative to act as a bimodal tracer of the fluorinated phase, enabling simultaneous monitoring via fluorescence and ^19^F‐NMR. Overall, these results demonstrate that co‐encapsulation with a fluorinated phase is an effective strategy for activating and optimizing the multimodal imaging performance of F‐BODIPY‐loaded PLGA NPs.

## 4 Experimental Section

### 4.1 Synthesis of Compound 7 and Physicochemical Characterization of F_54_‐BODIPY

#### 4.1.1 General

All available reagents were purchased from commercial sources and were used without any further purification. Solvents were purified by standard methods and dried if necessary. 4,4‐Difluoro‐8‐mesityl‐1,7‐dimethyl‐2,6‐diiodo‐3,5‐bis((E)‐4‐methylstyryl)‐4‐bora‐3a, 4a‐diaza‐s‐indacene (**6**) was prepared as described in the literature [15]. Thin layer chromatography (TLC) was conducted on plates precoated with silica gel Si 60‐F254 (Merck, Darmstadt, Germany). The detailed procedures for the synthesis of intermediates **3**, **4** and **5** are provided in the Supporting Information. Gravimetric column chromatography was carried out by using silica gel Si 60, 230–400 mesh, 0.040–0.063 mm (Merck, Darmstadt, Germany). Flash chromatography was carried out on J. T. Baker silica gel mesh size 230–400. Proton and heteronuclear NMR spectra were recorded at 298 ± 3 K on a Bruker Avance III 400 equipped with a 5 mm‐QNP probe (^19^F‐^31^P‐^13^C/^1^H); chemical shifts are indicated in parts per million downfield from SiMe_4_, using the residual proton (CHCl_3_ = 7.26 ppm, acetone‐d6 = 2.05 ppm) and carbon (CDCl_3_ = 77.0 ppm, acetone‐d6 = 29.84 ppm) solvent resonances as internal reference. Protons and carbon resonance assignments were established using ^13^C APT (jmod), 2D ^1^H‐^1^H COSY‐45 (cosyqf45), 2D ^13^C‐^1^H HETCOR (hxcoqf), and 2D ^1^H‐^13^C HMBC (low‐pass J filter) (hmbclpndqf) experiments. Coupling constants values J are given in Hz. Mass spectrometry analyses were performed at the MS facility of the Unitech COSPECT at the University of Milan (Italy) on a VG Autospec M246 (Fisons) spectrometer with EBE geometry, equipped with EI, and on a Q‐ToF SYNAPT G2‐S*i* HDMS 8K (Waters). Samples were dissolved in THF and diluited with MeOH (HPLC grade, ca. 10 μM).

#### 4.1.2 4,4‐Difluoro‐8‐mesityl‐1,7‐dimethyl‐2,6‐bis(2,6‐dimethylphenyl)‐3,5‐bis((E)‐4‐methylstyryl)‐4‐bora‐3a, 4a‐diaza‐s‐indacene (7)

A flame‐dried Schlenk tube equipped with a stir bar was loaded with BODIPY **6** (0.30 g, 0.365 mmol), (2,6‐dimethylphenyl)boronic acid (0.273 g, 2.61 mmol), dicyclohexyl(2′, 6‐dimethoxy‐[1,1‐biphenyl]‐2‐yl)phosphane (S‐PHOS, 60 mg, 0.146 mmol), palladium acetate (8.2 mg, 36.5 μmol), and tribasic potassium phosphate (0.62 g, 2.92 mmol). Cyclopentyl methyl ether (8 mL) was added; the final mixture was deaerated at low temperature (three times) and stirred vigorously at 110 °C. After 2‐h time, thin‐layer chromatography (EtP/CH_2_Cl_2_ 7:3) showed the complete conversion of the starting product.

After cooling to room temperature, the mixture was diluted with ethyl acetate (25 mL) and the organic phase was washed with water (three times with 10 mL). The organic phase was dried over MgSO_4_ and evaporated under reduced pressure. The resulting residue was purified by column gravimetric chromatography (silica gel, EtP/CH_2_Cl_2_ 7:3) to give the title compound (**7**, 0.253 g) in 89% yield as a purple metallic solid. ^1^H NMR (401 MHz, Acetone‐*d*_6_) *δ* 7.84 (*d*, *J* = 16.5 Hz, 2H), 7.38–7.25 (m, 2H), 7.25–7.01 (m, 14H), 6.38 (*d*, *J* = 16.6 Hz, 2H), 2.33 (s, 3H), 2.31 (s, 6H), 2.25 (s, 6H), 2.07 (s, 12H), 1.19 (s, 6H). ^13^C NMR (101 MHz, Acetone‐*d*_6_) *δ* 148.09, 138.21, 138.07, 137.75, 137.60, 136.18, 134.50, 134.19, 133.25, 133.16, 131.41, 130.70, 130.41, 128.58, 128.16, 127.18, 126.73, 125.88, 117.73, 19.46, 19.36, 18.48, 17.97, 9.19. MS (EI, 70 eV): *m/z* (%): 778.41 (100, [M]^+•^, calcd for C_54_H_53_BF_2_N_2_: 778.4270), 779.26 (60, [M + 1]^+•^, ^13^C_1_), 777.52 (25, [M − 1]^+•^, ^10^B), 661 (6, [M − C_9_H_9_]^+^, calcd for C_45_H_44_BF_2_N_2_^+^ 661.3566, loss of one (E)‐(4‐methylstyryl) radical from the 3‐ or 5‐position), 389.27 (4, [M]^2+^, doubly ionized molecular ion) (Figure S30).

#### 4.1.3 4,4‐(3‐(3‐(perfluoro‐tert‐butoxy)‐2,2‐bis(perfluoro‐tert‐butoxymethyl)propoxy)propoxy)‐8‐mesityl‐1,7‐dimethyl‐2,6‐bis(2,6‐dimethylphenyl)‐3,5‐bis((*E*)‐4‐methylstyryl)‐4‐bora‐3a, 4a‐diaza‐*s*‐indacene (F_54_‐BODIPY)

BODIPY **7** (0.138 g, 0.177 mmol) was dissolved in dry CH_2_Cl_2_ (7 mL) at 50 °C; solid AlCl_3_ (71 mg, 0.531 mmol) was added and the mixture was stirred at 50 °C for 5 min. The violet‐blue solution was then cooled at room temperature before adding dropwise a solution of **5** (0.790 g, 0.887 mmol) in a mixture of CH_2_Cl_2_ (5 mL) and the minimum amount of THF (0.5 mL). The reaction was followed by TLC (EtP/Et_2_O 98:2). After 2 h at room temperature, the starting product had completely disappeared. The solvents were removed by evaporation under reduced pressure, and the solid residue was purified by flash chromatography column (silica gel, EtP/Et_2_O, 98:2) collecting the first dark blue fraction. This was then redissolved in the minimum amount of Et_2_O, and MeOH (20 mL) was added. The mixture was left overnight during which a precipitate was formed, which was filtered to give the title compound (**F**_**54**_ **‐BODIPY**, 360 mg) as indigo crystalline solid in 81% yield. ^1^H NMR (401 MHz, Acetone‐*d*_6_) *δ* 8.36 (*d*, *J* = 16.8 Hz, 2H), 7.28 (dd, *J* = 8.5, 6.5 Hz, 2H), 7.20 (*d*, *J* = 7.5 Hz, 4H), 7.12 (s, 8H), 7.09 (s, 2H), 6.25 (*d*, *J* = 16.8 Hz, 2H), 4.14 (s, 12H), 3.33–3.24 (m, 8H), 3.20 (*t*, *J* = 6.6 Hz, 4H), 2.33 (s, 3H), 2.29 (s, 6H), 2.26 (s, 6H), 2.08 (s, 12H), 1.47–1.38 (m, 4H), 1.38–1.26 (m, 8H), 1.24–1.13 (m, 10H). ^13^C NMR (101 MHz, Acetone‐*d*_6_) *δ* 149.93, 139.61, 139.21, 139.07, 137.97, 137.59, 136.35–135.67 (m), 134.63, 134.12, 133.46, 131.77, 130.16, 129.90, 128.76, 128.46, 127.70, 122.00, 121.14 (*q*, *J* = 292.9 Hz), 82.16–79.31 (m), 72.11, 66.38, 65.73, 61.98, 47.19, 33.15, 30.03, 27.57, 26.71, 21.21, 20.30, 19.53, 11.03. ^19^F NMR (377 MHz, Acetone‐*d*_6_) *δ* −70.99 ppm. HRMS (ESI): *m/z*: calcd for C_100_H_93_BF_54_N_2_O_10_^+•^ [M]^+•^: 2518.6077; found: 2518.6069 (*Δ* = −0.3 ppm); calcd for C_100_H_93_BF_54_N_2_NaO_10_^+^ [M + Na]^+^: 2540.5957; found: 2540.6025 (*Δ* = +2.7 ppm). Characteristic fragments: *m/z* 1629.5189 [M − OR]^+^ (calcd for C_77_H_73_BF_27_N_2_O_5_^+^: 1629.5181, *Δ* = +0.5 ppm); 1628.5211 [M − ROH]^+•^ (calcd for C_77_H_72_BF_27_N_2_O_5_^+•^: 1628.5103, *Δ* = +6.6 ppm); 771.4487 [(dipy)B(OMe)]^+^ (calcd for C_55_H_56_BN_2_O^+^: 771.4486, *Δ* = +0.1 ppm); 757.4322 [(dipy)B(OH)]^+^ (calcd for C_54_H_54_BN_2_O^+^: 757.4329, *Δ* = −0.9 ppm); 756.4279 [(dipy)B=O]^+•^ (calcd for C_54_H_53_BN_2_O^+•^: 756.4251, *Δ* = +3.7 ppm). *R* = —(CH_2_)_6_OCH_2_C(CH_2_OC(CF_3_)_3_)_3_; dipy = C_54_H_53_N_2_ (Figures S31 and S32).

### 4.2 Formulation Procedure for the PLGA NPs Loaded With the F‐BODIPYs

Initially, the hydrophobic components 1.1 μmol PLGA, (Resomer RG 504 H, Poly (D, L‐lactide‐co‐glycolide) 50:50 having M_w_ = 38,000–54,000 g/mol) and 6.1 μmol of F‐BODIPY were dissolved in 5 mL ethyl acetate. An aqueous solution of the amphiphilic surfactant was prepared by dissolving 17 μmol Pluronic F68 (average *M* *_w_* = 8350 g/mol) in Milli Q. The ratio between the organic and aqueous solvents was optimized as 1:2 as a low organic‐aqueous ratio will lead to faster evaporation and yield smaller NPs. The two phases were mixed, tip‐sonicated at 40% amplitude for 40 s and then magnetically stirred at 700 rpm at room temperature for 3 h to ensure the complete formation and stabilization of the polymeric nanodroplets. The organic solvent was then removed by rotary evaporation under reduced pressure (up to 80 mbar) at room temperature. Finally, the samples were centrifuged for 1 h at 8500 rpm at 20 °C and the pellet was resuspended in 2 ml of water. Samples were left to rest for one night to eliminate the non‐stable precipitate. The FB‐NPs were synthesized in the presence and absence of a second liquid perfluorinated agent PFCE. PFCE was added to the sample mixture after the addition of surfactant and before the tip sonication at F‐BODIPY:PFCE ratio1:9.

## Funding

This work was supported by the Ministero dell'Università e della Ricerca (2022598YAX and 2017MYBTXC).

## Conflicts of Interest

The authors declare no conflicts of interest.

## Supporting information

Further experimental details, including synthetic procedures for intermediates **2‐5**, single‐crystal X‐ray diffraction experiment and crystallographic data, and nanoparticles characterization methods are included.

## Data Availability

The data that supports the findings of this study are available in the supplementary material of this article.

## References

[1] X. Wang , Q. Ding , R. R. Groleau , et al., “Fluorescent Probes for Disease Diagnosis,” Chemical Reviews 124 (2024): 7106. 38760012 10.1021/acs.chemrev.3c00776 PMC11177268

[2] J. Bischof , G. Fletcher , P. Verkade , et al., “Multimodal Bioimaging Across Disciplines and Scales: Challenges, Opportunities and Breaking Down Barriers,” npj Imaging 2 (2024): 5. 40603654 10.1038/s44303-024-00010-w PMC12118647

[3] B. L. Bona , O. Koshkina , C. Chirizzi , V. Dichiarante , P. Metrangolo , and F. Baldelli Bombelli , “Multibranched‐Based Fluorinated Materials: Tailor‐Made Design of ^19^F‐MRI Probes,” Accounts of Materials Research 4 (2023): 71.

[4] I. Tirotta , V. Dichiarante , C. Pigliacelli , et al., “ ^19^F Magnetic Resonance Imaging (MRI): From Design of Materials to Clinical Applications,” Chemical Reviews 115 (2015): 1106. 25329814 10.1021/cr500286d

[5] D. Janasik and T. Krawczyk , “ ^19^F MRI Probes for Multimodal Imaging,” Chemistry—A European Journal 28 (2022): e202102556. 34705306 10.1002/chem.202102556

[6] O. Maxouri , Z. Bodalal , M. Daal , et al., “How to ^19^F MRI: Applications, Technique, and Getting Started,” BJR|Open 5 (2023): 20230019. 37953866 10.1259/bjro.20230019 PMC10636348

[7] C. Guo , M. Xu , S. Xu , and L. Wang , “Multifunctional nanoprobes for both fluorescence and ^19^F magnetic resonance imaging,” Nanoscale 9 (2017): 7163. 28513699 10.1039/c7nr01858d

[8] A. M. Huynh , A. Müller , S. M. Kessler , et al., “Small BODIPY Probes for Combined Dual ^19^F MRI and Fluorescence Imaging,” ChemMedChem. (2016): 1568. 27347843 10.1002/cmdc.201600120

[9] Y. Zhang , S. Bo , T. Feng , et al., “A Versatile Theranostic Nanoemulsion for Architecture‐Dependent Multimodal Imaging and Dually Augmented Photodynamic Therapy,” Advanced Materials 31 (2019): 1806444. 10.1002/adma.201806444 30907469

[10] A. Li , F. Wang , M. Jiang , Y. Li , X. Zhou , and Z. Jiang , “Fluorinated Hypoxia‐Responsive Aza‐BODIPY for NIR‐II FL/ ^19^F MR/PA Imaging and Phototherapy of Lung Cancer,” Advanced Science 13 (2026): e21886. 41549220 10.1002/advs.202521886 PMC13042524

[11] G. Mandriota , N. Depalo , E. Fanizza , et al., “Encapsulation of PERFECTA Fluorinated Probe in a Core–Shell Nanostructured Lipid Carrier for ^19^F‐MRI Molecular Imaging,” ACS Applied Nano Materials 9 (2026): 3679–3688.

[12] I. Tirotta , A. Mastropietro , C. Cordiglieri , et al., “A Superfluorinated Molecular Probe for Highly Sensitive in Vivo ^19^F‐MRI,” Journal of the American Chemical Society 136 (2014): 8524. 24884816 10.1021/ja503270n

[13] E. T. Ahrens and U. Flögel , “Fluorine Magnetic Resonance Imaging,” Fluorine Magnetic Resonance Imaging: Methods and Applications in Biomedicine (Jenny stanford publishing, 2024), 1.

[14] C. Chirizzi , D. De Battista , I. Tirotta , et al., “Multispectral MRI With Dual Fluorinated Probes to Track Mononuclear Cell Activity in Mice,” Radiology 291 (2019): 351. 30888930 10.1148/radiol.2019181073

[15] M. I. Martinez Espinoza , L. Sori , A. Pizzi , et al., “BODIPY Dyes Bearing Multibranched Fluorinated Chains: Synthesis, Structural, and Spectroscopic Studies,” Chemistry – A European Journal 25 (2019): 9078. 31184410 10.1002/chem.201901259

[16] L. Chen , S. Lei , J. Zhang , et al., “Hierarchical Assembly of Fluorinated BODIPY‐Based Nanoribbons for Highly Efficient Metal‐Free Photocatalytic Hydrogen Production,” Journal of the American Chemical Society 148 (2026): 11127. 41771044 10.1021/jacs.5c22856

[17] A. Loudet and K. Burgess , “BODIPY Dyes and Their Derivatives: Syntheses and Spectroscopic Properties,” Chemical Reviews 107 (2007): 4891. 17924696 10.1021/cr078381n

[18] D. Tian , F. Qi , H. Ma , et al., “Domino‐Like Multi‐Emissions Across Red and Near Infrared From Solid‐State 2‐/2,6‐Aryl Substituted BODIPY Dyes,” Nature Communications 9 (2018): 2688. 10.1038/s41467-018-05040-8 PMC6043560 30002375

[19] A. M. Gómez , L. Infantes , J. Ticona‐Chambi , et al., “Naturally J‐Aggregated F‐BODIPYs: Self‐Assembly Organization Driven by Substitution Pattern,” Journal of Molecular Liquids 394 (2024): 123773.

[20] Z. Liu , Z. Jiang , M. Yan , and X. Wang , “Recent Progress of BODIPY Dyes With Aggregation‐Induced Emission,” Frontiers in Chemistry 7 (2019): 712. 31709235 10.3389/fchem.2019.00712 PMC6824186

[21] T. Patten , S. H. Röttger , P. G. Jones , D. B. Werz , and A. Krawczuk , “Tuning Solid‐State Fluorescence in BODIPY Dyes through Substitution‐Controlled Packing Motifs,” Advanced Optical Materials 14 (2026): e02067.

[22] T. Ozdemir , S. Atilgan , I. Kutuk , et al., “Solid‐State Emissive BODIPY Dyes With Bulky Substituents as Spacers,” Organic Letters 11 (2009): 2105. 19419209 10.1021/ol9005568

[23] A. C. Benniston and G. Copley , “Lighting the Way Ahead With Boron Dipyrromethene (Bodipy) Dyes,” Physical Chemistry Chemical Physics 11 (2009): 4124. 19458813 10.1039/b901383k

[24] R. B. Weinberg , Mark Muyskens , “An Iodine Fluorescence Quenching Clock Reaction,” Journal of Chemical Education 84 (2007): 797.

[25] L. A. Antina , A. A. Ksenofontov , A. V. Kazak , N. V. Usol’tseva , E. V. Antina , and M. B. Berezin , “Effect of Ms‐Substitution on Aggregation Behavior and Spectroscopic Properties of BODIPY Dyes in Aqueous Solution, Langmuir‐Schaefer and Poly(methyl Methacrylate) Thin Films,” Colloids and Surfaces A: Physicochemical and Engineering Aspects 618 (2021): 126449.

[26] E. Hoogendijk , E. Swider , A. H. J. Staal , et al., “Continuous‐Flow Production of Perfluorocarbon‐Loaded Polymeric Nanoparticles: From the Bench to Clinic,” ACS Applied Materials and Interfaces 12 (2020): 49335. 33086007 10.1021/acsami.0c12020 PMC7645868

