# Supplementary material for: Design and Characterization of Dual‐Modal Fluorinated BODIPY Probes
Source: Chempluschem. 2026 Aug 13;91(8):e70222. doi: 10.1002/cplu.70222 (PMC13472697; doi:10.1002/cplu.70222)
Supplement: Supplementary file 1 [file CPLU-91-e70222-s001.pdf]

SUPPORTING INFORMATION

Design and characterization of dual-modal fluorinated BODIPY probes

Nazeeha Ayaz+[a], Marta Rosati+[a], Marco Damoli[a], Johnny Camilo Perez Rosas[a], Andrea Pizzi[a], Pierangelo Metrangolo[a], Simonetta Orlandi[b], Marta Penconi[c], Marco Cavazzini*[b] and Francesca Baldelli Bombelli*[a]

[a] Laboratory of Supramolecular and Bio-Nanomaterials (SupraBioNano Lab), Department of Chemistry, Materials and Chemical Engineering, “Giulio Natta”, Politecnico di Milano, 20133 Milan, Italy
[b] Institute of Chemical Sciences and Technologies “Giulio Natta” (CNR-SCITEC), National Research Council (CNR), via C. Golgi 19, 20133 Milan, Italy
[c] Institute of Chemical Sciences and Technologies “Giulio Natta” (CNR-SCITEC), National Research Council (CNR), via Fantoli 16/15, 20138 Milan, Italy

Table of Contents

Methods **3**

Single-crystal X-ray diffraction *3*
Figure S1. Chemical structure of **F_{54}-$(CH_2)_3$BODIPY**. 4
Table S1. Crystallographic data for **F_{54}-$(CH_2)_3$BODIPY**. 4

Dynamic Light Scattering *5*
Figure S2. a) Intensity-weighted average size distribution at 90° for sample formulation of **F_{54}-NPs** and **F_{54}P-NPs**, upon the addition of PFCE; b) Intensity-weighted average size distribution at 90° for sample formulation of **F_{54}I-NPs** and **F_{54}IP-NPs**, upon the addition of PFCE. 5

^{19}F-NMR on PLGA Nanoparticles *5*
Figure S3. ^{19}F-NMR spectrum of a) **F_{54}-NPs** and PFCE and b) **F_{54}IP-NPs** and PFCE. Solvent:H_2O/D_2O (10:1 v/v). 6
Figure S4. ^{19}F NMR spectrum of **F_{54}-BODIPY**. 6

Z potential *6*

UV-Vis and Fluorescence Spectroscopy *6*
Figure S5. a) Absorbance spectra and b) fluorescence spectra of **F_{54}I-NPs** and **F_{54}IP-NPs**; c) ^{19}F-NMR spectrum of **F_{54}I-NPs** and **F_{54}IP-NPs** showing a sharper peak of F-BODIPY at -71.11 ppm and the shift of the peak to -71.8 ppm upon addition of PFCE. 7

Synthesis of intermediates **8**

Figure S6. Molecular structure and table of ^{1}H NMR assignments of 4,4-difluoro-8-mesityl-1,7-dimethyl-2,6-bis(2,6-dimethylphenyl)-3,5-bis((E)-4-methylstyryl)-4-bora-3a,4a-diaza-s-indacene (**7**). 9
Figure S7. ^{1}H NMR spectrum of 4,4-difluoro-8-mesityl-1,7-dimethyl-2,6-bis(2,6-dimethylphenyl)-3,5-bis((E)-4-methylstyryl)-4-bora-3a,4a-diaza-s-indacene (**7**). 10

Figure S8. ^{1}H NMR spectrum of 4,4-difluoro-8-mesityl-1,7-dimethyl-2,6-bis(2,6-dimethylphenyl)-3,5-bis((E)-4-methylstyryl)-4-bora-3a,4a-diaza-s-indacene (**7**): a) SW = 8.00 – 6.30 ppm; b) SW = 2.50 – 1.10 ppm. 11
Figure S9. Bidimensional COSYQF45 of 4,4-difluoro-8-mesityl-1,7-dimethyl-2,6-bis(2,6-dimethylphenyl)-3,5-bis((E)-4-methylstyryl)-4-bora-3a,4a-diaza-s-indacene (**7**): a) full spectrum; b) SW(f1,f2) = 8.30 – 6.00 ppm. 12
Figure S10. Molecular structure and table of ^{13}C NMR tentative assignments of 4,4-difluoro-8-mesityl-1,7-dimethyl-2,6-bis(2,6-dimethylphenyl)-3,5-bis((E)-4-methylstyryl)-4-bora-3a,4a-diaza-s-indacene (**7**). 13
Figure S11. ^{13}C NMR spectrum of 4,4-difluoro-8-mesityl-1,7-dimethyl-2,6-bis(2,6-dimethylphenyl)-3,5-bis((E)-4-methylstyryl)-4-bora-3a,4a-diaza-s-indacene (**7**). 14
Figure S12. ^{13}C NMR spectrum of 4,4-difluoro-8-mesityl-1,7-dimethyl-2,6-bis(2,6-dimethylphenyl)-3,5-bis((E)-4-methylstyryl)-4-bora-3a,4a-diaza-s-indacene (**7**): a) SW = 141.0 – 135.0 ppm; b) SW = 132.0 – 118.0 ppm. 15
Figure S13. Bidimensional HXCOQF spectra of 4,4-difluoro-8-mesityl-1,7-dimethyl-2,6-bis(2,6-dimethylphenyl)-3,5-bis((E)-4-methylstyryl)-4-bora-3a,4a-diaza-s-indacene (**7**): a) full spectrum; b) SW(f1) = 8.10 – 6.10 ppm, SW(f2) = 142.0 – 115.0 ppm. 16
Figure S14. Bidimensional HMBCLPNDQF spectrum of 4,4-difluoro-8-mesityl-1,7-dimethyl-2,6-bis(2,6-dimethylphenyl)-3,5-bis((E)-4-methylstyryl)-4-bora-3a,4a-diaza-s-indacene (**7**): a) full spectrum; b) SW(f1) = 154.0 – 123.0 ppm, SW(f2) = 8.00 – 6.20 ppm. 17
Figure S15. Molecular structure and table of ^{1}H NMR assignments of compound **F_{54}-BODIPY**. 19
Figure S16. ^{1}H NMR spectrum of compound **F_{54}-BODIPY**. 20
Figure S17. ^{1}H NMR spectrum of compound **F_{54}-BODIPY**: a) SW = 8.80 – 6.00 ppm; b) SW = 7.35 – 7.00 ppm. 21
Figure S18. ^{1}H NMR spectrum of compound **F_{54}-BODIPY**: a) SW = 4.40 – 3.00 ppm; b) SW = 2.40 – 1.00 ppm. 22
Figure S19. Bidimensional COSYQF90 of compound **F_{54}-BODIPY**: a) full spectrum; b) SW(f1,f2) = 7.50 – 6.90 ppm. 23
Figure S20. Molecular structure and table of ^{13}C NMR tentative assignments **F_{54}-BODIPY**. 24
Figure S21. ^{13}C NMR spectrum of **F_{54}-BODIPY**. 25
Figure S22. ^{13}C NMR spectrum of **F_{54}-BODIPY**: a) SW = 152.4 – 132.4 ppm; b) SW = 132.5 – 127.0 ppm. 26
Figure S23. ^{13}C NMR spectrum of **F_{54}-BODIPY**: a) SW = 127.2 – 57.6 ppm; b) SW = 52.0 – 6.0 ppm. 27
Figure S24. ^{13}C-APT NMR spectrum of **F_{54}-BODIPY.** 28
Figure S25. Bidimensional HXCOQF spectra of **F_{54}-BODIPY**: a) full spectrum; b) SW(f1) = 8.60 – 6.00 ppm, SW(f2) = 137.0 – 120.0 ppm. 29
Figure S26. Bidimensional HXCOQF spectra of **F_{54}-BODIPY**. a) SW(f1) = 4.50 – 2.85 ppm, SW(f2) = 75.0 – 60.0 ppm; b) SW(f1) = 2.60 – 0.80 ppm, SW(f2) = 34.5 – 8.0 ppm. 30
Figure S27. Bidimensional HMBCLPNDQF spectrum of **F_{54}-BODIPY**: a) full spectrum; b) SW(f1) = 154.0 – 123.0 ppm, SW(f2) = 8.60 – 6.00 ppm. 31
Figure S28. Bidimensional HMBCLPNDQF spectrum of **F_{54}-BODIPY**: a) SW(f1) = 70.5 – 43.0 ppm, SW(f2) = 4.40 – 3.00 ppm; b) SW(f1) = 142.0 – 125.0 ppm, SW(f2) = 2.50 – 1.00 ppm. 32
Figure S29. MS-EI^{+} of 4,4-difluoro-8-mesityl-1,7-dimethyl-2,6-bis(2,6-dimethylphenyl)-3,5-bis((E)-4-methylstyryl)-4-bora-3a,4a-diaza-s-indacene (**7**). 33
Figure S30. Molecular-ion cluster of 4,4-difluoro-8-mesityl-1,7-dimethyl-2,6-bis(2,6-dimethylphenyl)-3,5-bis((E)-4-methylstyryl)-4-bora-3a,4a-diaza-s-indacene (**7**) in MS-EI^{+}. 34
Figure S31. HRMS-ESI^{+} of compound **F_{54}-BODIPY**. 35
Figure S32. Molecular-ion cluster of compound **F_{54}-BODIPY** in HRMS-ESI^{+}: a) simulated, b) experimental. 36

References **37**

Methods

Single-crystal X-ray diffraction

Several crystallization conditions have been tested for **F_{54}-BODIPY,** including slow evaporation of pure organic solvents or binary/ternary mixtures of organic solvents. The general procedure consists in dissolving 3 mg of the compound in the selected solvent (1-3 mL based on solubility) in a borosilicate glass vial, allowing the solvent to evaporate slowly under a laminar flow hood. In the case of suspensions or not completely clear solutions, sonication and gentle heating were applied. Some precipitates form within 3 days.

Table S0. Crystallization tests done on F_{54}-BODIPY

**Solvent**	**Result**
DCM	Amorphous material
Hexane	Microcrystalline powder
Acetone	**Crystals**
Ethyl acetate	Amorphous material
Benzene	Amorphous material
Chloroform	Amorphous material
Carbon tetrachloride	Amorphous material
Ethyl acetate/Methanol 1:1	Amorphous material
Ethyl acetate/Ethanol 1:1	**Crystals**
Ethyl acetate/Acetonitrile 1:1	Amorphous material
Acetonitrile/Ethanol/ Ethyl acetate 1:1:1	Amorphous material
Acetone/Methanol 1:1	Microcrystalline powder
Acetone/Ethanol 1:1	Amorphous material
Acetone/Acetonitrile 1:1	Amorphous material
Chloroform/Methanol 1:1	Amorphous material
Chloroform /Ethanol 1:1	Amorphous material
Chloroform /Acetonitrile 1:1	Amorphous material

The best crystals, obtained from both acetone and an ethyl acetate/ethanol (1:1) solvent mixture, were examined using an XtaLAB Synergy-S single-crystal X-ray diffractometer. Despite their promising morphology, the crystals exhibited poor diffraction quality, even with extended exposure times. We were just able to determine a reasonable unit cell and the space group, reported as it follows:

a. 11.7918(15)	α. 90	Space group P21/m
b. 62.366(16)	β. 94.417(10)	Cell volume (Å^3) 12211(4)
c. 16.653(2)	γ. 90	

Single crystal X-ray diffraction data of compound **F_{54}-$(CH_2)_3$BODIPY (Figure S1)** were collected on a Bruker APEX-II diffractometer equipped with sealed-tube and CCD detector, using Mo-Kα radiation (λ = 0.71073 Å). Data collection was performed at room temperature (296 K) to avoid undesired phase transitions occurring at lower T. The diffraction data were indexed, integrated and scaled using SAINT. [**2012**, DOI: https://journals.iucr.org/e/services/stdswrefs.html].The structures were solved by SHELXS[1] and refinements were carried out by full-matrix least squares on F^2 using the SHELXL program.[2] Crystals appear as thin colored, low-diffracting plates. Hydrogen atoms were included at calculated positions through HFIX instruction. Anisotropic thermal motion refinement was used for all the atoms. Geometric restraints on bond lengths and angles (DFIX, DANG) and thermal motion parameters restrains (ISOR) have been applied on disordered and poorly defined moieties, in particular on the fluorinated regions. Some –CF_3 groups were treated as disordered with independent free variables (FVAR). Pictures were prepared using CCDC Mercury.[3]

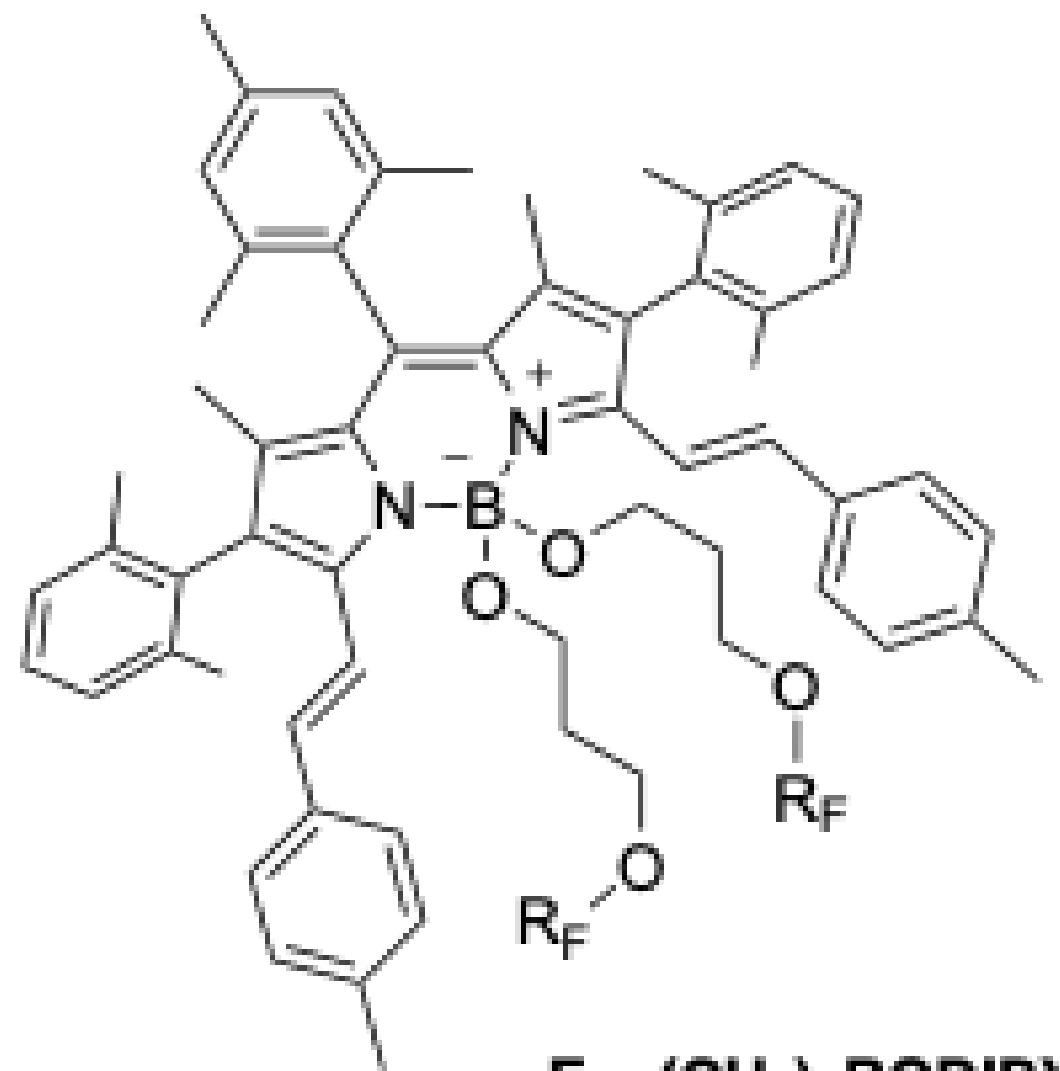

Figure S1. Chemical structure of **F_{54}-$(CH_2)_3$BODIPY**.

Table S1. Crystallographic data for **F_{54}-$(CH_2)_3$BODIPY**.

**Identification code**	F_{54}-$(CH_2)_3$BODIPY
**CCDC deposition number**	2531948
**Empirical formula**	$C_{94}H_{81}BF_{54}N_2O_{10}$
**Formula weight**	2435.41
**Temperature/K**	296.15
**Crystal system**	monoclinic
**Space group**	C2/c
**a/Å**	62.247(2)
**b/Å**	19.9003(8)
**c/Å**	19.3011(8)
**α/°**	90
**β/°**	106.586(2)
**γ/°**	90
**Volume/Å^3**	22914.1(16)
**Z**	8
**ρ_{calc}g/cm^3**	1.412
**μ/mm^{-1}**	0.150
**F(000)**	9840.0
**Crystal size/mm^3**	0.1 × 0.1 × 0.05
**Radiation**	MoKα (λ = 0.71073)
**2Θ range for data collection/°**	3.218 to 36.7
**Index ranges**	-54 ≤ h ≤ 54, -17 ≤ k ≤ 17, -17 ≤ l ≤ 16
**Reflections collected**	77116
**Independent reflections**	8241 [R_{int} = 0.0998, R_{sigma} = 0.0495]
**Data/restraints/parameters**	8241/697/1501
**Goodness-of-fit on F^2**	2.285
**Final R indexes [I>=2σ (I)]**	R_1 = 0.1921, wR_2 = 0.4827
**Final R indexes [all data]**	R_1 = 0.2461, wR_2 = 0.5394
**Largest diff. peak/hole / e Å^{-3}**	0.84/-0.62

Dynamic Light Scattering

An ALV apparatus equipped with ALV-5000/EPP Correlator, special optical fiber detector and ALV/CGS-3 Compact goniometer was used for making size measurements with DLS. The light source was a He-Ne laser (λ = 633 nm), 22 mW output power. Measurements were performed at 25 °C by transferring ≈ 1 mL of sample solution into a cylindrical Hellma scattering cell. Data analysis was performed according to standard procedures and autocorrelation functions were analyzed through a constrained regularization method (Laplace inversion of the time auto-correlation functions), CONTIN, for obtaining the particle size distribution.

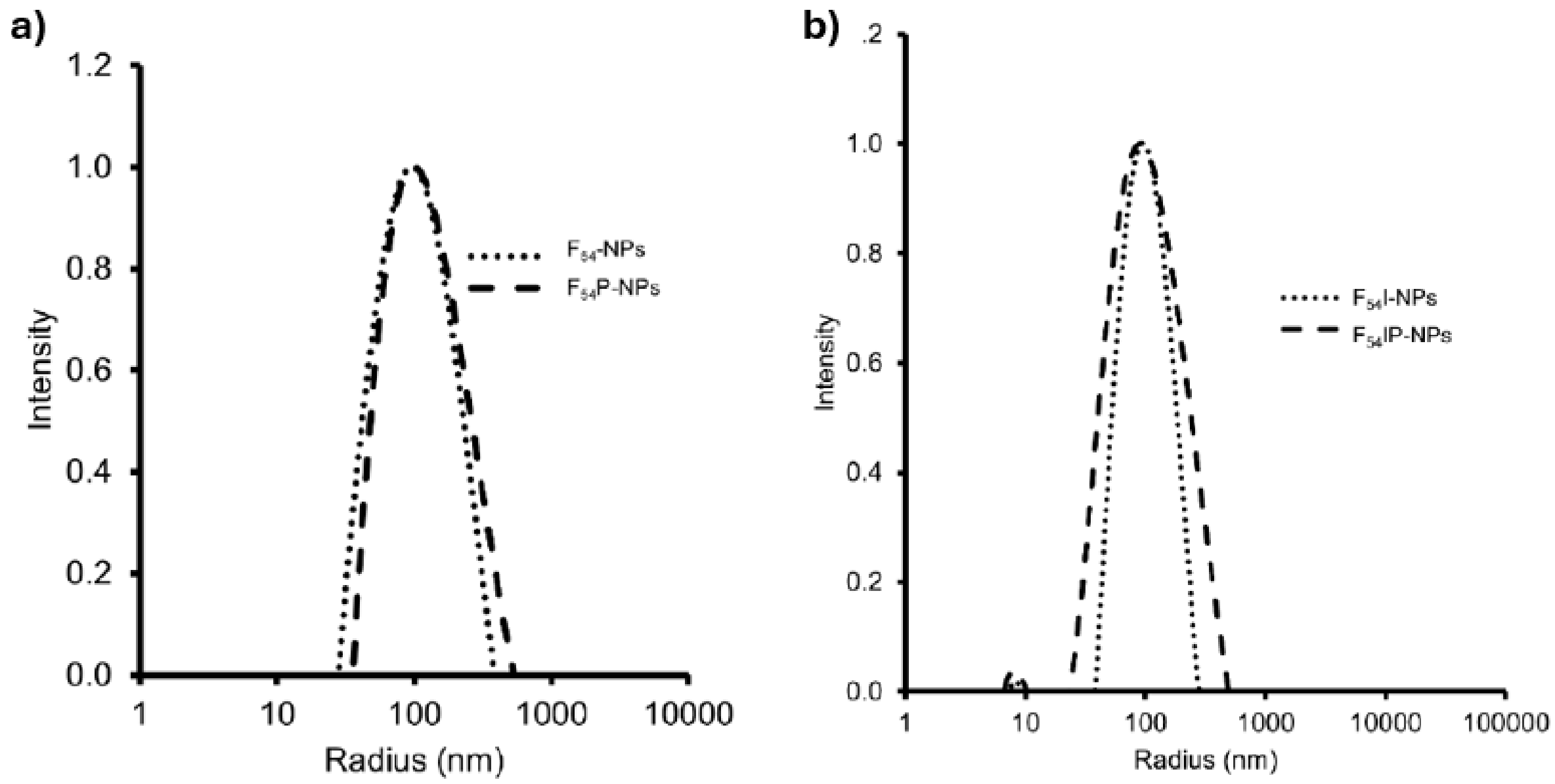

Figure S2. a) Intensity-weighted average size distribution at 90° for sample formulation of **F_{54}-NPs** and **F_{54}P-NPs**, upon the addition of PFCE; b) Intensity-weighted average size distribution at 90° for sample formulation of **F_{54}I-NPs** and **F_{54}IP-NPs**, upon the addition of PFCE.

^{19}F-NMR on PLGA Nanoparticles

All the NMR spectra were recorded on a Bruker AV400 Bruker AvanceIII 400 MHz spectrometer equipped with a 5 mm QNP probe (^{19}F, ^{31}P–^{13}C/^{1}H). NMR spectra were recorded at (300 ± 3 K). To 400 μL of BODIPY NPs aqueous dispersions, 40μL of D_2O was added in the ratio 1:10 v/v for ^{19}F-NMR measurements. Data were processed with MestreNova 10.0 from Mestre-Lab.

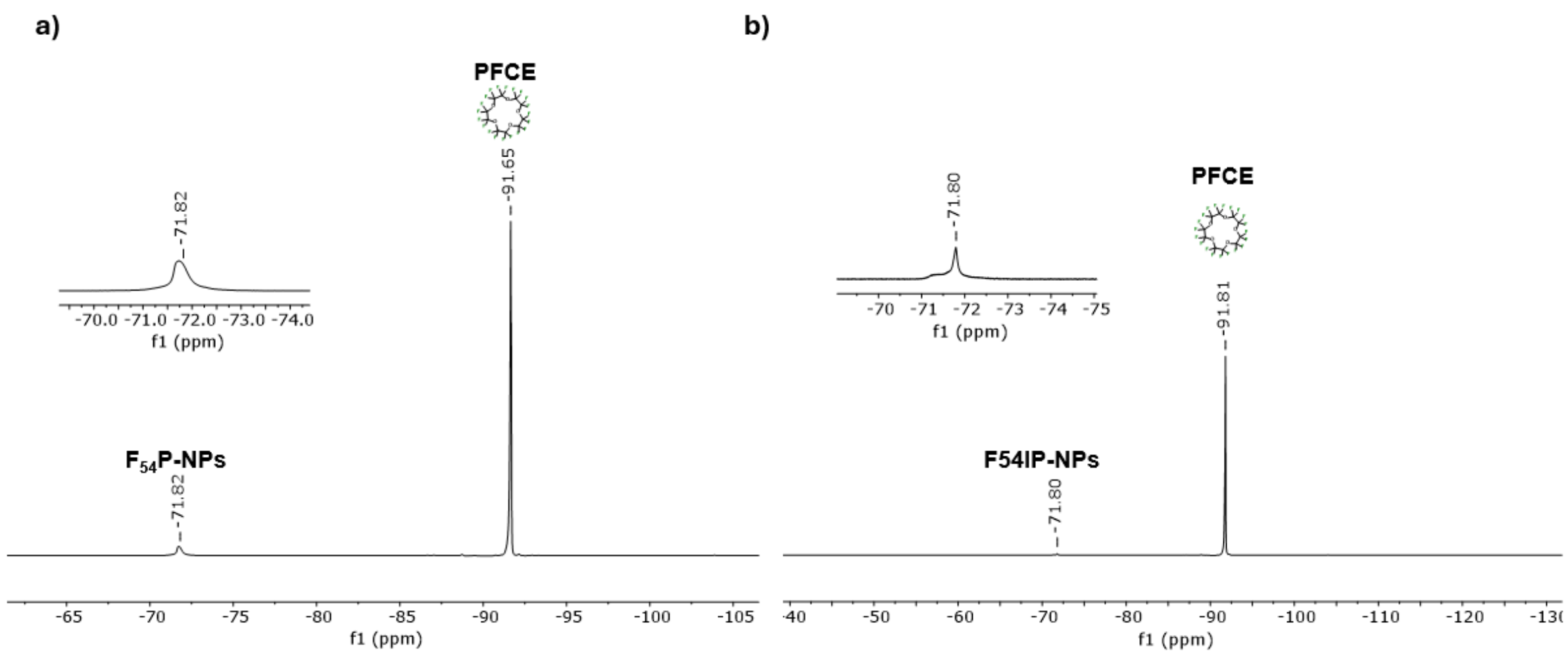

Figure S3. ^{19}F-NMR spectrum of a) **F_{54}-NPs** and PFCE and b) **F_{54}IP-NPs** and PFCE. Solvent:H_2O/D_2O (10:1 v/v).

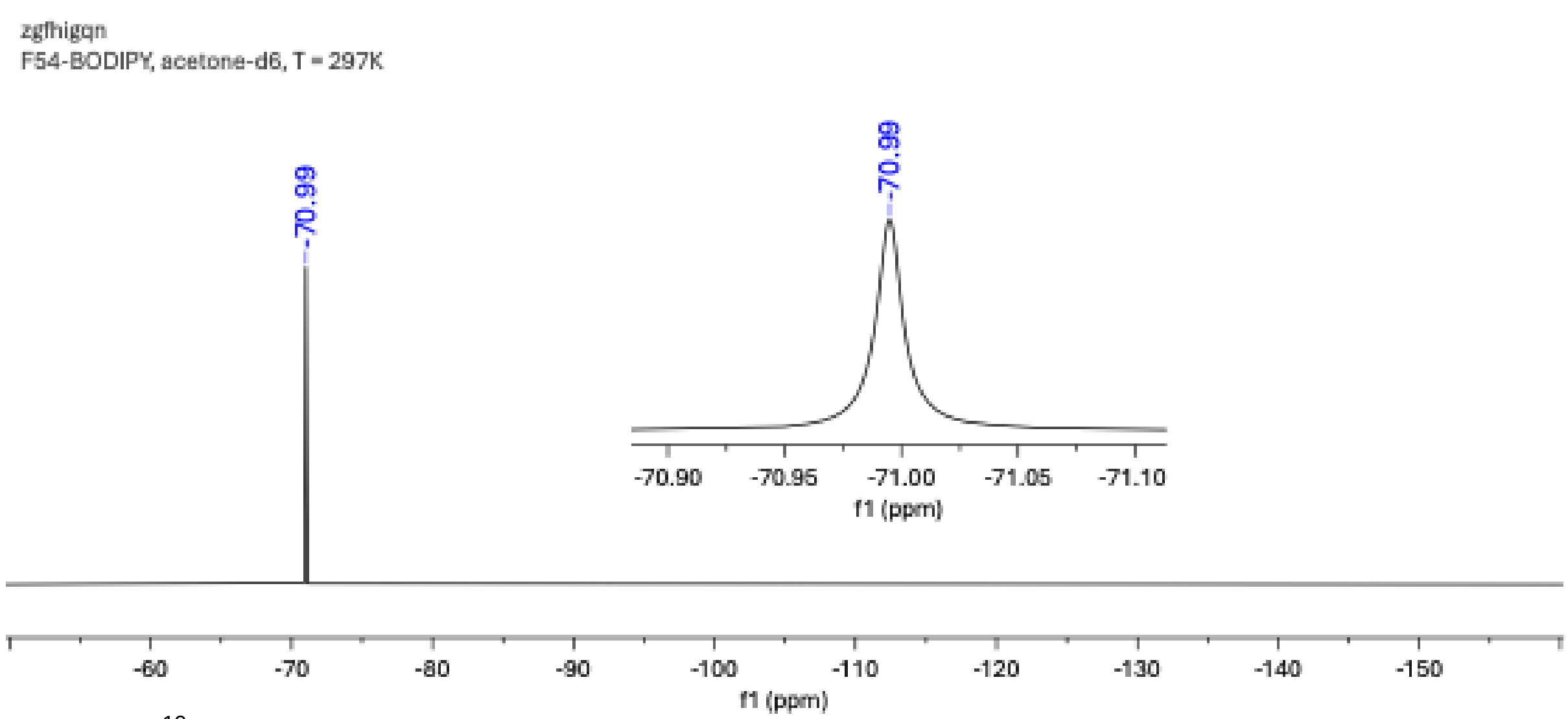

Figure S4. ^{19}F NMR spectrum of **F_{54}-BODIPY**.

Z potential

Zetasizer Nano ZS (Malvern Instrument, Malvern, Worcestershire, UK), equipped with a 633 nm red laser and measuring the scattered light at an angle of 173° was used to measure the surface charge of the samples. The analysis was performed at a temperature of 25 °C in folded capillary cells, i.e., U-shaped cells with two Au plated beryllium/copper electrodes at the top. The cells were primarily washed with Milli Q water and conditioned with the sample dispersant before loading ≈ 0.8 mL of sample solution, ensuring that the Au-plated electrodes were immersed and no bubbles were present inside the cell.

UV-Vis and Fluorescence Spectroscopy

The absorption spectra were recorded with a Shimadzu UV-3600 plus and a Jasco V-630 spectrophotometers in 1 cm path length quartz cell. Luminescence quantum efficiencies (QE) were measured using a Hamamatsu C9920 system equipped with a xenon lamp, calibrated

integrating sphere and C10027 photonic multichannel analyser. Absolute fluorescence quantum yields were acquired in solutions and nanoformulations at absorbance < 0.1 at the excitation wavelength to avoid reabsorption and inner filter effect. Steady state emission and excitation spectra and photoluminescence lifetimes were obtained with a FLS 980 spectrofluorimeter (Edinburgh Instrument Ltd.). Continuous excitation for the steady state measurements was provided by a 450 W Xenon arc lamp. Emission spectra were corrected for the wavelength-dependent sensitivity of the detector. Photoluminescence time-resolved measurements were carried out by TCSPC (time-correlated single-photon counting) method with an Edinburgh Picosecond Pulsed Diode Laser EPL-375 (Edinburgh Instrument Ltd.). Spectra present in figures 3c (main text) and S5b were reordered with a commercial spectrofluorometer (Jasco, FP-8500) at λex = 620 nm and λem = 630 - 730 nm.

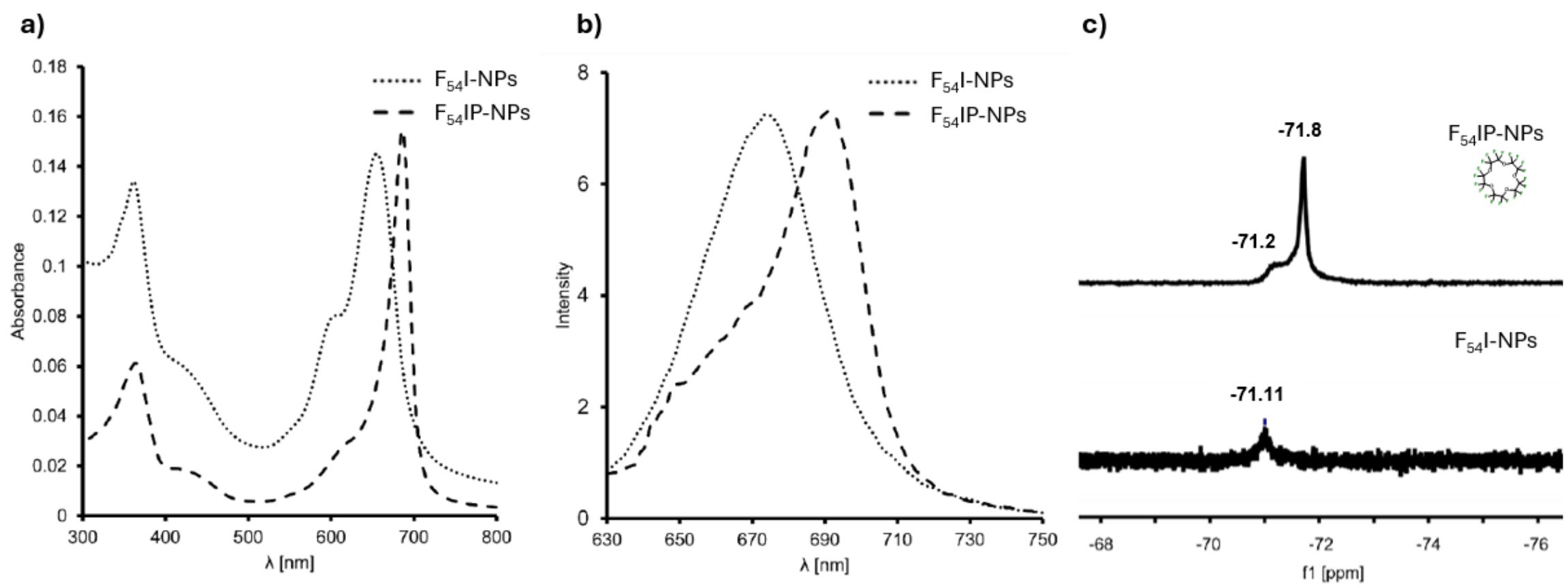

Figure S5. a) Absorbance spectra and b) fluorescence spectra of **F_{54}I-NPs** and **F_{54}IP-NPs**; c) ^{19}F-NMR spectrum of **F_{54}I-NPs** and **F_{54}IP-NPs** showing a sharper peak of F-BODIPY at -71.11 ppm and the shift of the peak to -71.8 ppm upon addition of PFCE.

Synthesis of intermediates

Synthetic strategy was adapted from already reported procedures.[4, 5]

6-Benzyloxy-1-bromo-hexane (**2**)

A solution of 6-bromo-1-hexan-1-ol (2 g, 11 mmol) and benzyl bromide (1.2 mL, d = 1.438, 10 mmol) in dry THF (13 mL) was cooled to 0°C in an ice-water bath and stirred for 10 min. Sodium hydride (440 mg, 60% mineral oil dispersion, 11 mmol) was added and the mixture was stirred at low temperature for 1 h and then the bath was removed, and the mixture was stirred at room temperature overnight. The mixture was quenched with water and extracted with DCM. The organic phase was washed with water, dried over $MgSO_4$, filtered and the solvent was removed under vacuum affording the title compound 6-benzyloxy-1-bromo-hexane_(**2**, 1.71 g) in a quantitative yield as an oil. Physical and spectroscopic data agree with those reported in the literature.[6]

2-[(6-(benzyloxy)hexyloxy)methyl]-2-(hydroxymethyl)propane-1,3-diol (**3**)

To a suspension of KOH (290 mg, 5.17 mmol) in DMSO (5 mL) solid pentaerythritol **1** (682 mg, 5 mmol) was added and the mixture was stirred until complete dissolution. A solution of **2** (1.39 g, 5.14 mmol) in DMSO (3 mL) was then added and the mixture left stirring overnight. The reaction was quenched with water and extracted with AcOEt. The organic phase was washed thoroughly with water, dried over $MgSO_4$, filtered and the solvent was removed under vacuum. Purification of the crude material by chromatography (silica gel, hexane/AcOEt 2:8 →100% AcOEt →AcOEt/MeOH (95:5), afforded the title compound 2-[(6-(benzyloxy)hexyloxy)methyl]-2-(hydroxymethyl)propane-1,3-diol (**3**, 620 mg) in 38% yield as an oil. ^{1}H NMR (400 MHz, $CDCl_3$) δ 7.42 – 7.27 (m, 5H), 4.50 (s, 2H), 3.71 (s, 6H), 3.53 – 3.38 (m, 7H), 2.03 (s, 8H), 1.67-1.53 (m, 4H), 1.45-1.30 (m, 4H). ^{13}C NMR (100.6 MHz, $CDCl_3$) δ 138.70, 128.51, 127.81, 127.68, 73.59, 73.03, 72.10, 70.44, 65.17, 44.90, 29.81, 29.50, 26.11, 26.08.

1-Benzyloxy-6-((3-nonafluoro-tert-butoxy)-2,2-bis((nonafluoro-tert-butoxy)-methyl))-propoxy-hexane (**4**)

To a solution of PPh_3 (9.6 g, 36.6 mmol) in dry Et_2O (40 mL), molecular sieves 4Å (2g) were added under inert atmosphere together with a solution of triol **3** (2 g, 6.12 mmol) in dry Et_2O (10 mL). The mixture was cooled to 0°C and nonafluoro-*tert*-butyl alcohol was added (5.1 mL, d = 1.693, 36.5 mmol) followed by the dropwise addition of a solution of DIAD (7.2 mL, d= 1.027, 36.6 mmol) in dry Et_2O (10 mL). The formation of a white precipitate was observed. The solution was kept at low temperature for 10 min, then allowed warm to room temperature and stirred. After 6 hat room tem perature, the flask was heated at 30°C in an oil bath and stirred at this temperature for 40 h. After cooling, the mixture was filtered under vacuum to remove the solid material and the solvent was removed under reduced pressure. The crude was purified by chromatography (silica gel, hexane/AcOEt (95:5) to afford the title compound **4** (4.44 g) in 74% yield as a transparent oil. ^{1}H NMR (400 MHz, $CDCl_3$) δ 7.40 – 7.28 (m, 5H), 4.50 (s, 2H), 4.03 (s, 6H), 3.46 (t, J = 6.6 Hz, 2H), 3.42 – 3.29 (m, 4H), 1.71 – 1.46 (m, 4H), 1.46 – 1.22 (m, 4H). ^{13}C NMR (100.6 MHz, $CDCl_3$) δ 138.66, 128.37, 127.64, 127.53, 120.44 (q, J = 296 Hz, CF_3), 72.92, 71.59, 70.36, 65.58, 65.42, 46.22, 29.73, 29.34, 26.05, 25.91.

6-((3-Nonafluoro-tert-butoxy)-2,2-bis((nonafluoro-tert-butoxy)-methyl))-propoxy-hexane-1-ol (**5**)

To a solution of the substrate **4** (1.2 g, 1.22 mmol) in AcOEt (15 mL) was added palladium on charcoal (10% Pd/C, 100 mg) and the mixture was hydrogenated at atmospheric pressure and room temperature. At the end of the reaction, the mixture was filtered over a short plug of Celite and the solvent was removed under vacuum to afford the title compound (**5**, 1.0 g) in 92% yield as a dense oil. ^{1}H NMR (400 MHz, $CDCl_3$) δ 4.03 (s, 6H), 3.65 (t, J = 6.6 Hz, 2H), 3.44 – 3.28 (m, 4H), 1.61-1.49 (m, 4H), 1.41-1.26 (m, 4H). ^{13}C NMR (100.6 MHz, $CDCl_3$) δ 120.27 (q, J = 294 Hz, CF_3), 71.65, 65.73, 65.55, 63.06, 46.34, 32.81, 29.47, 25.96, 25.70.

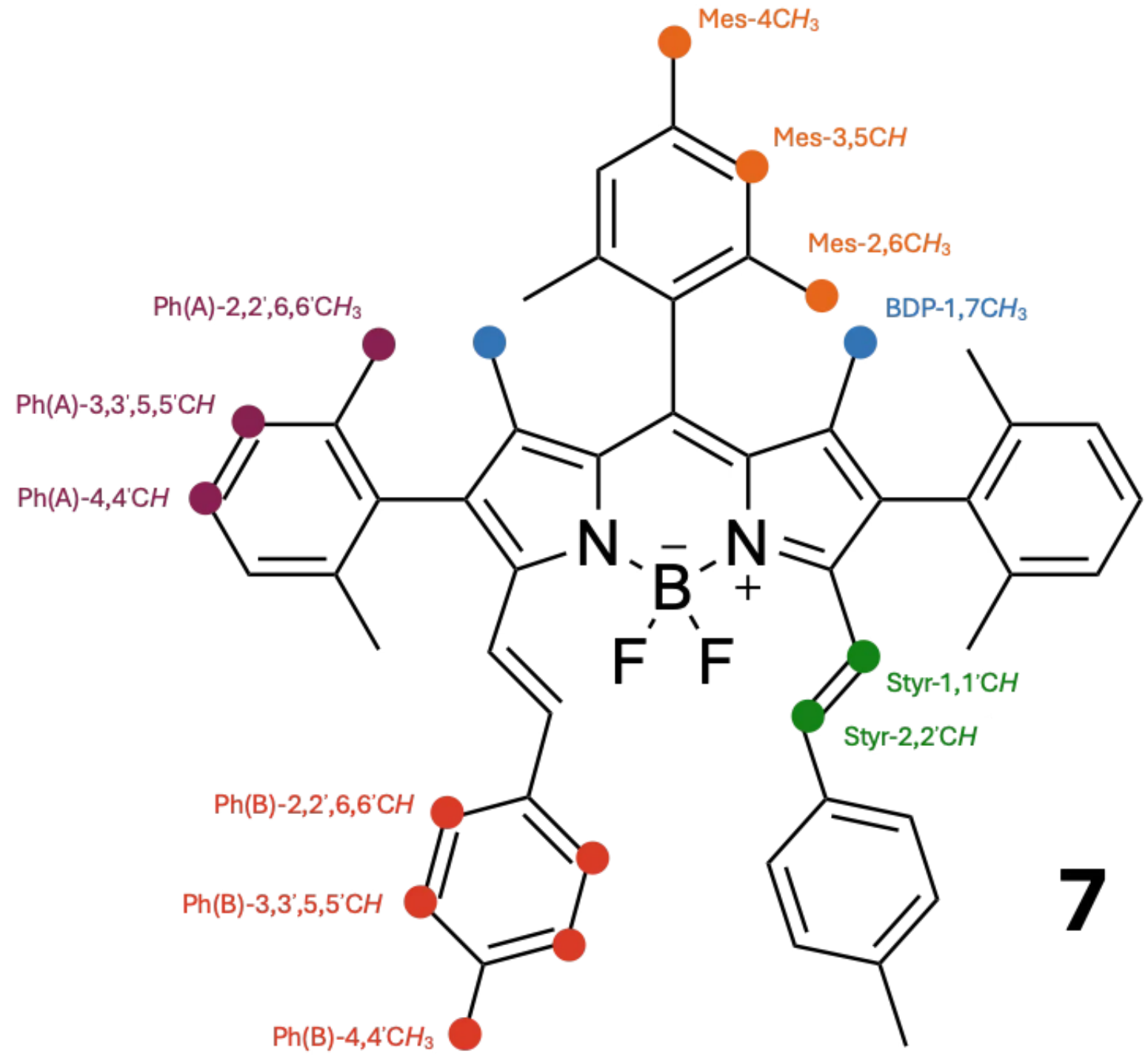

7

	Assignment	Shift	Range	H's	Integral	Multiplicity	J (Hz)
**1**	Styr-1,1'C*H*	7.84	7.91 .. 7.78	2	2.00	d	16.5
**2**	Ph(A)-4,4'C*H*	7.31	7.38 .. 7.25	2	2.03	m	
**3**	Mes-3,5C*H*<br>Ph(B)-2,2',6,6'C*H*<br>Ph(B)-3,3',5,5'C*H*<br>Ph(A)-3,3',5,5'C*H*	7.15	7.25 .. 7.01	14	13.93	m	
**4**	Styr-2,2'C*H*	6.38	6.44 .. 6.32	2	2.01	d	16.5
**5**	Mes-4CH_3	2.33	2.36 .. 2.32	3	3.02	s	
**6**	Mes-2,6CH_3	2.31	2.32 .. 2.29	6	6.11	s	
**7**	Ph(B)-4,4'CH_3	2.25	2.27 .. 2.23	6	6.05	s	
**8**	Ph(A)-2,2',6,6'CH_3	2.07	2.10 .. 2.06	12	12.23	s	
**9**	BDP-1,7CH_3	1.19	1.23 .. 1.16	6	5.96	s	

Figure S6. Molecular structure and table of ^{1}H NMR assignments of 4,4-difluoro-8-mesityl-1,7-dimethyl-2,6-bis(2,6-dimethylphenyl)-3,5-bis((E)-4-methylstyryl)-4-bora-3a,4a-diaza-s-indacene (**7**).

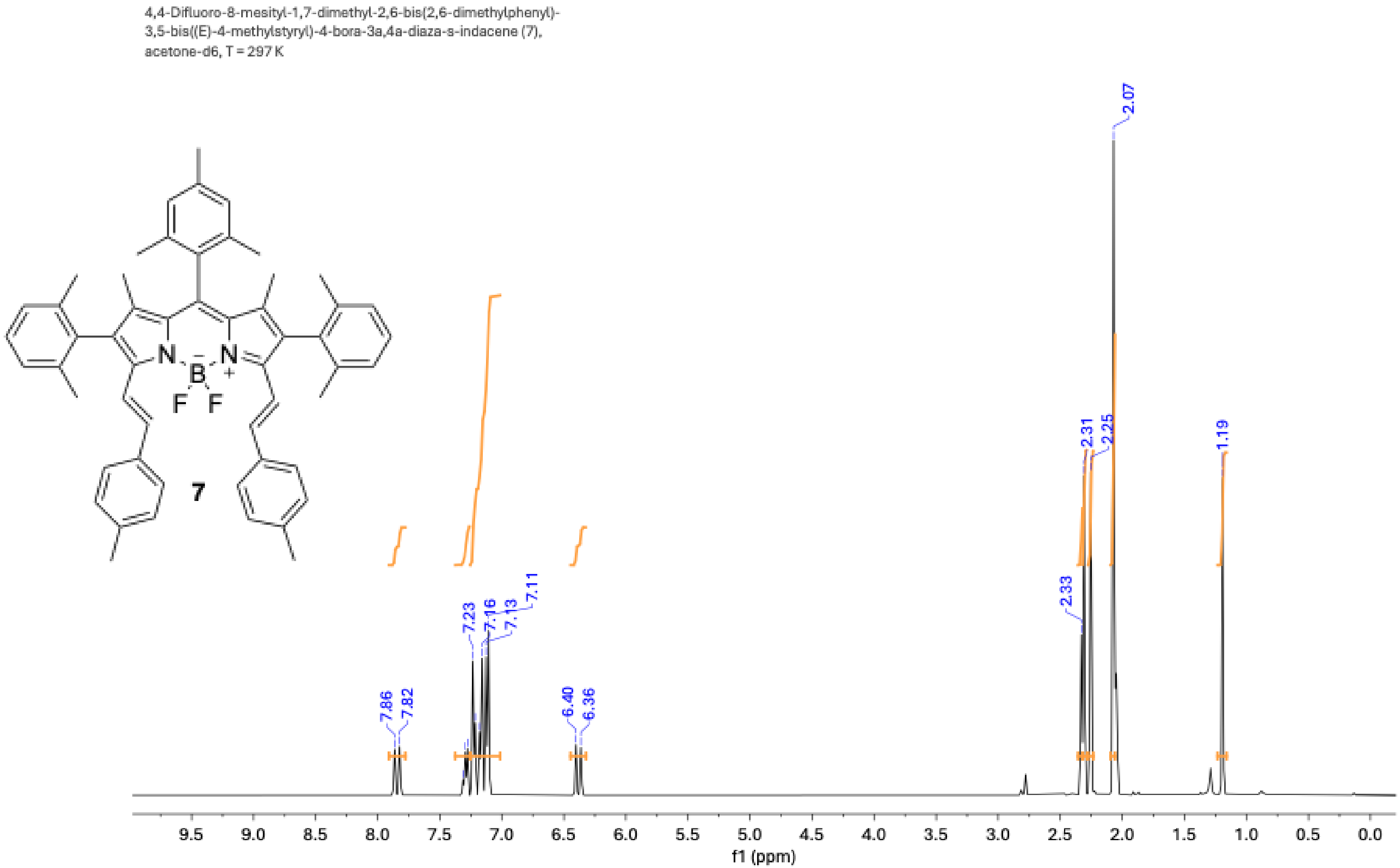

Figure S7. ^{1}H NMR spectrum of 4,4-difluoro-8-mesityl-1,7-dimethyl-2,6-bis(2,6-dimethylphenyl)-3,5-bis((E)-4-methylstyryl)-4-bora-3a,4a-diaza-s-indacene (**7**).

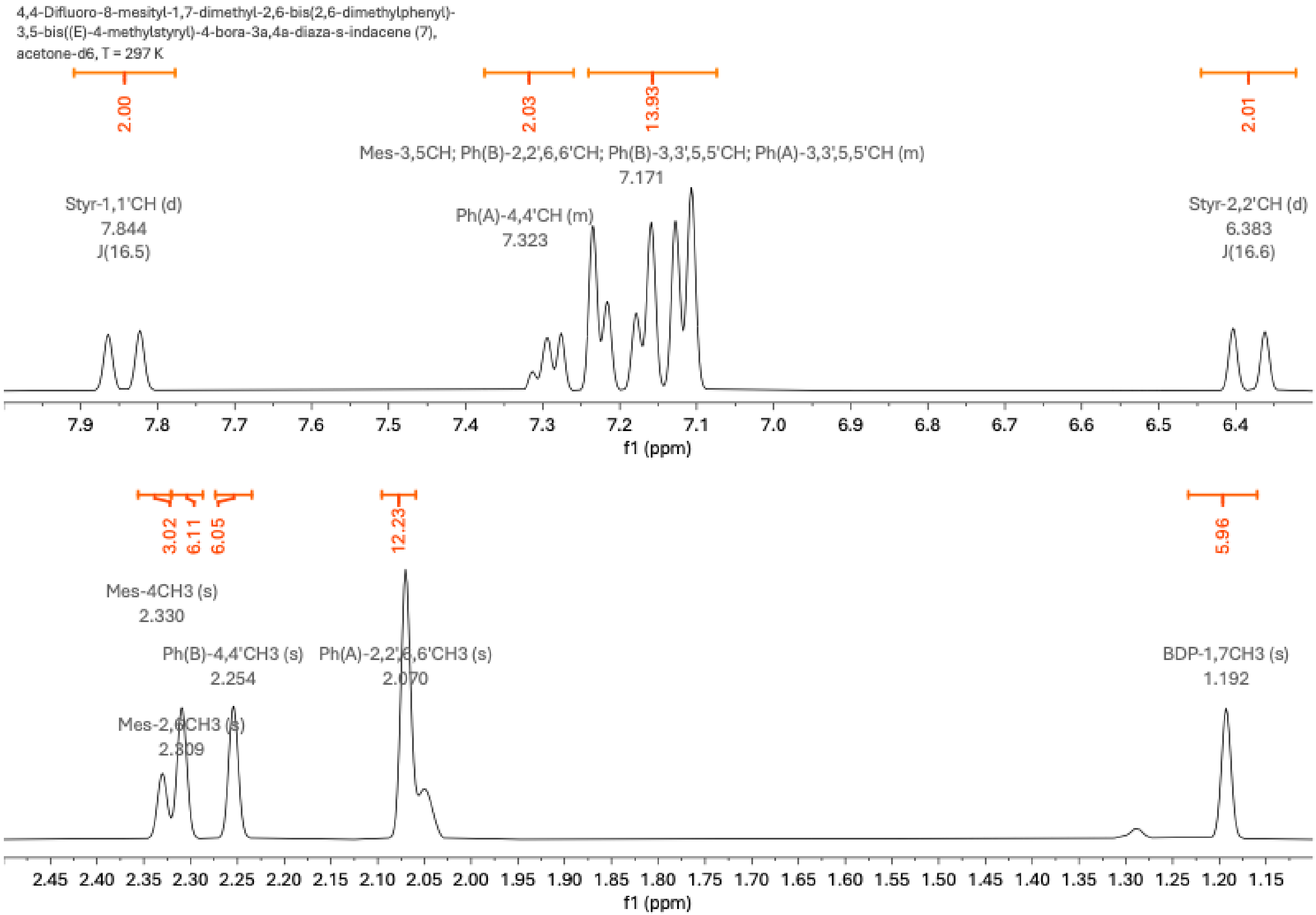

Figure S8. ^{1}H NMR spectrum of 4,4-difluoro-8-mesityl-1,7-dimethyl-2,6-bis(2,6-dimethylphenyl)-3,5-bis((E)-4-methylstyryl)-4-bora-3a,4a-diaza-s-indacene (**7**): a) SW = 8.00 – 6.30 ppm; b) SW = 2.50 – 1.10 ppm.

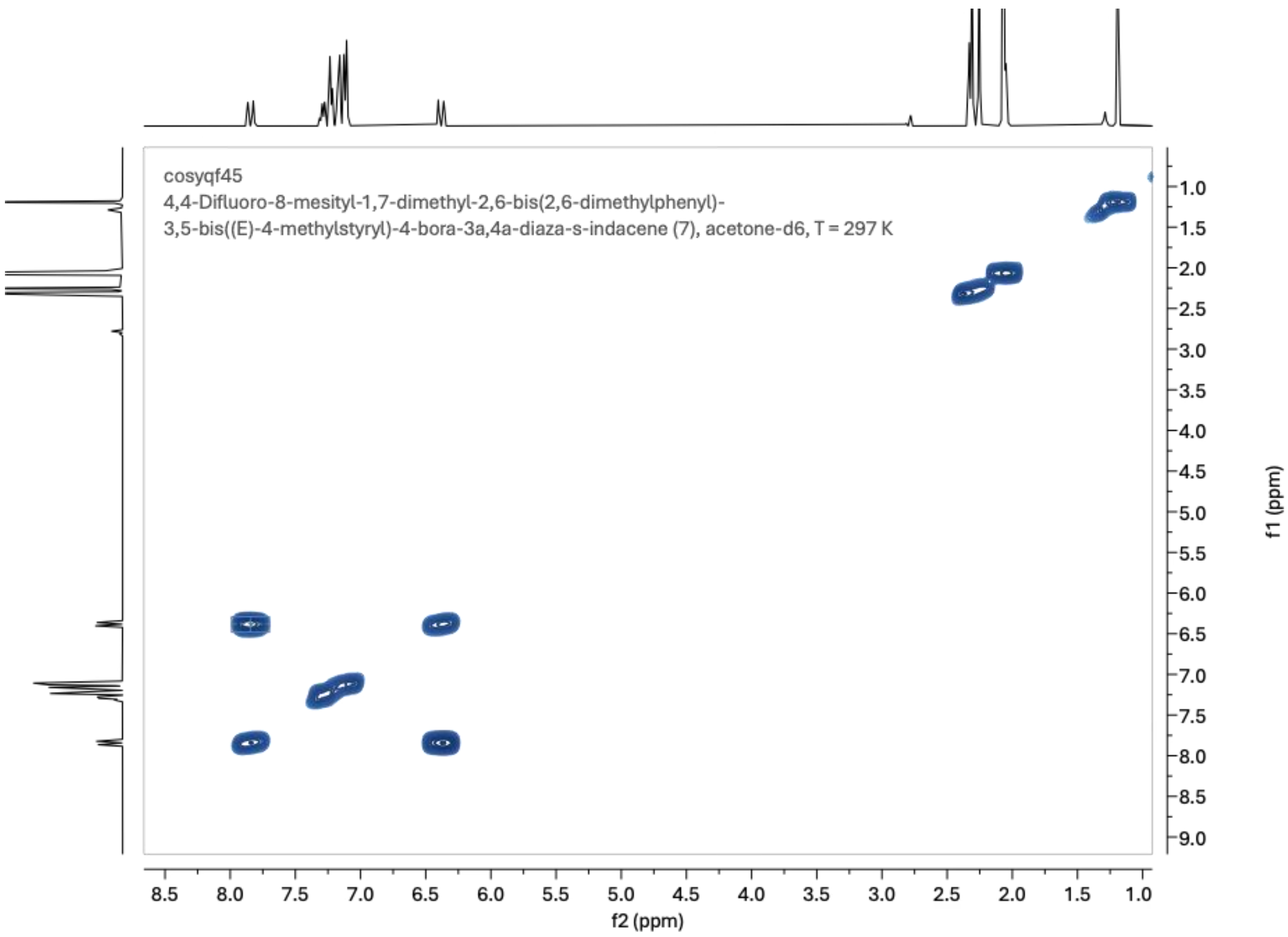

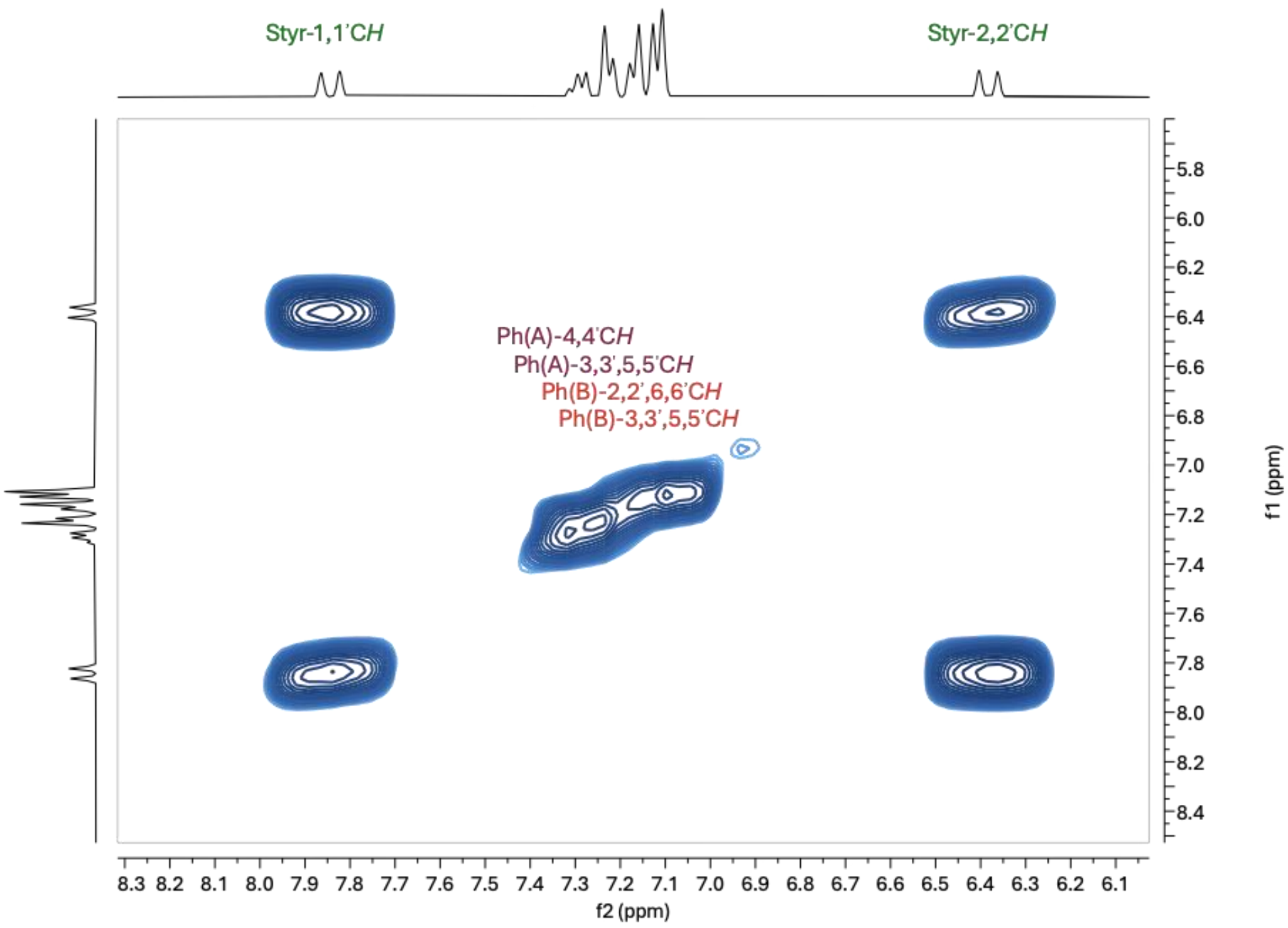

Figure S9. Bidimensional COSYQF45 of 4,4-difluoro-8-mesityl-1,7-dimethyl-2,6-bis(2,6-dimethylphenyl)-3,5-bis((E)-4-methylstyryl)-4-bora-3a,4a-diaza-s-indacene (**7**): a) full spectrum; b) SW(f1,f2) = 8.30 – 6.00 ppm.

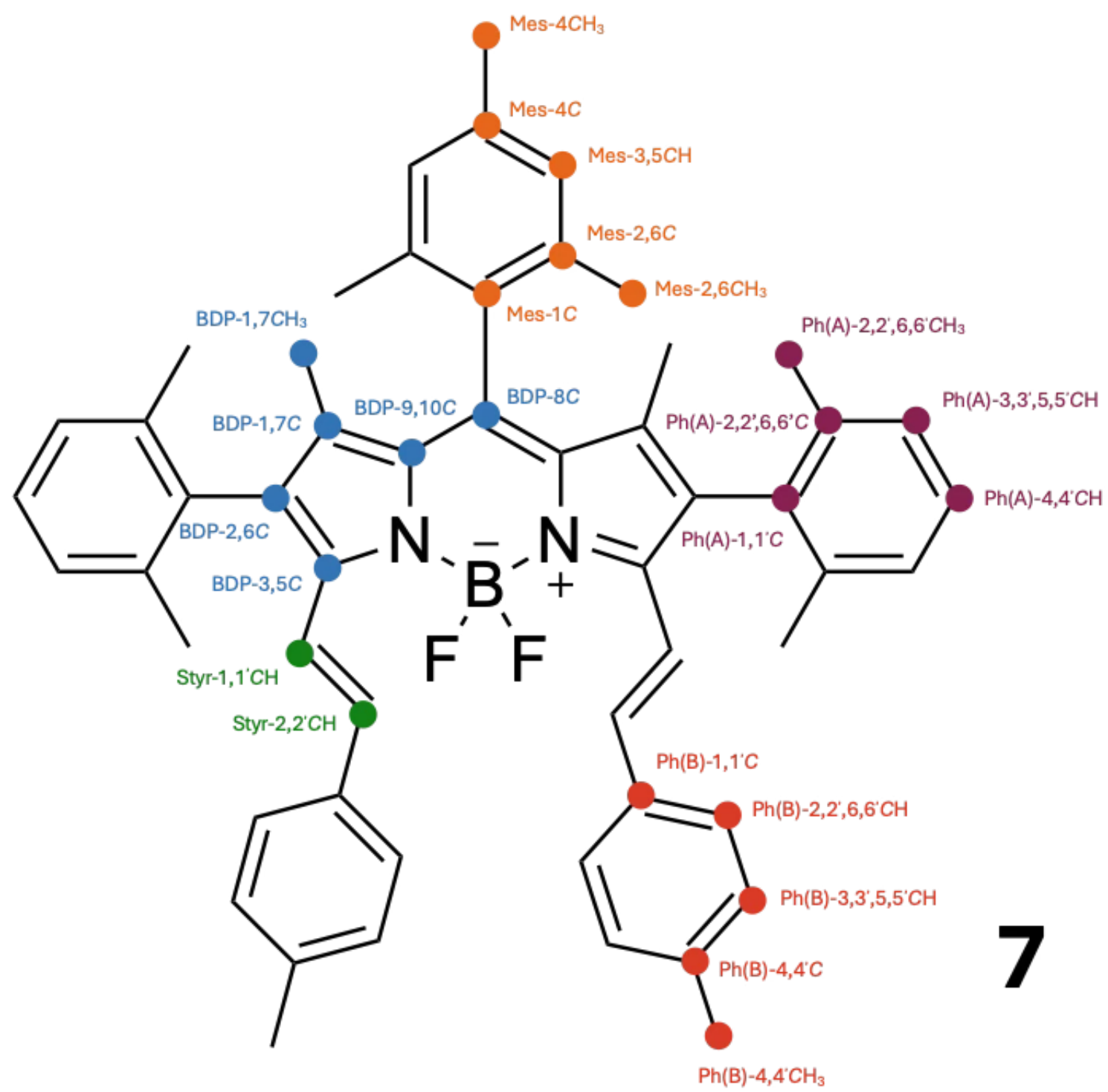

7

	Assignment	Shift
1	BDP-3,5*C*	149.96
2	BDP-9,10*C*	140.09
3	BDP-8*C*	139.95
4	Ph(B)-1,1'*C*	139.63
5	Mes-1*C*	139.48
6	Ph(A)-1,1'*C*	138.06
7	Styr-2,2'*C*H	136.38
8	Ph(B)-4,4'*C*	136.07
9	BDP-1,7*C*	135.13
10	Mes-4*C*	135.04
11	Ph(A)-2,2',6,6'*C*	133.29
12	Mes-2,6*C*	132.58
13	BDP-2,6*C*	132.29
14	Ph(B)-3,3',5,5'*C*H	130.46
15	Ph(A)-4,4'*C*H	130.04
16	Mes-3,5*C*H	129.06
17	Ph(A)-3,3',5,5'*C*H	128.61
18	Ph(B)-2,2'6,6'*C*H	127.76
19	Styr-1,1'*C*H	119.61
20	Mes-2,6*C*H_3	21.34
21	Mes-4*C*H_3	21.24
22	Ph(A)-2,2',6,6'*C*H_3	20.36
23	Ph(B)-4,4'*C*H_3	19.85
24	BDP-1,7*C*H_3	11.07

Figure S10. Molecular structure and table of ^{13}C NMR tentative assignments of 4,4-difluoro-8-mesityl-1,7-dimethyl-2,6-bis(2,6-dimethylphenyl)-3,5-bis((E)-4-methylstyryl)-4-bora-3a,4a-diaza-s-indacene (**7**).

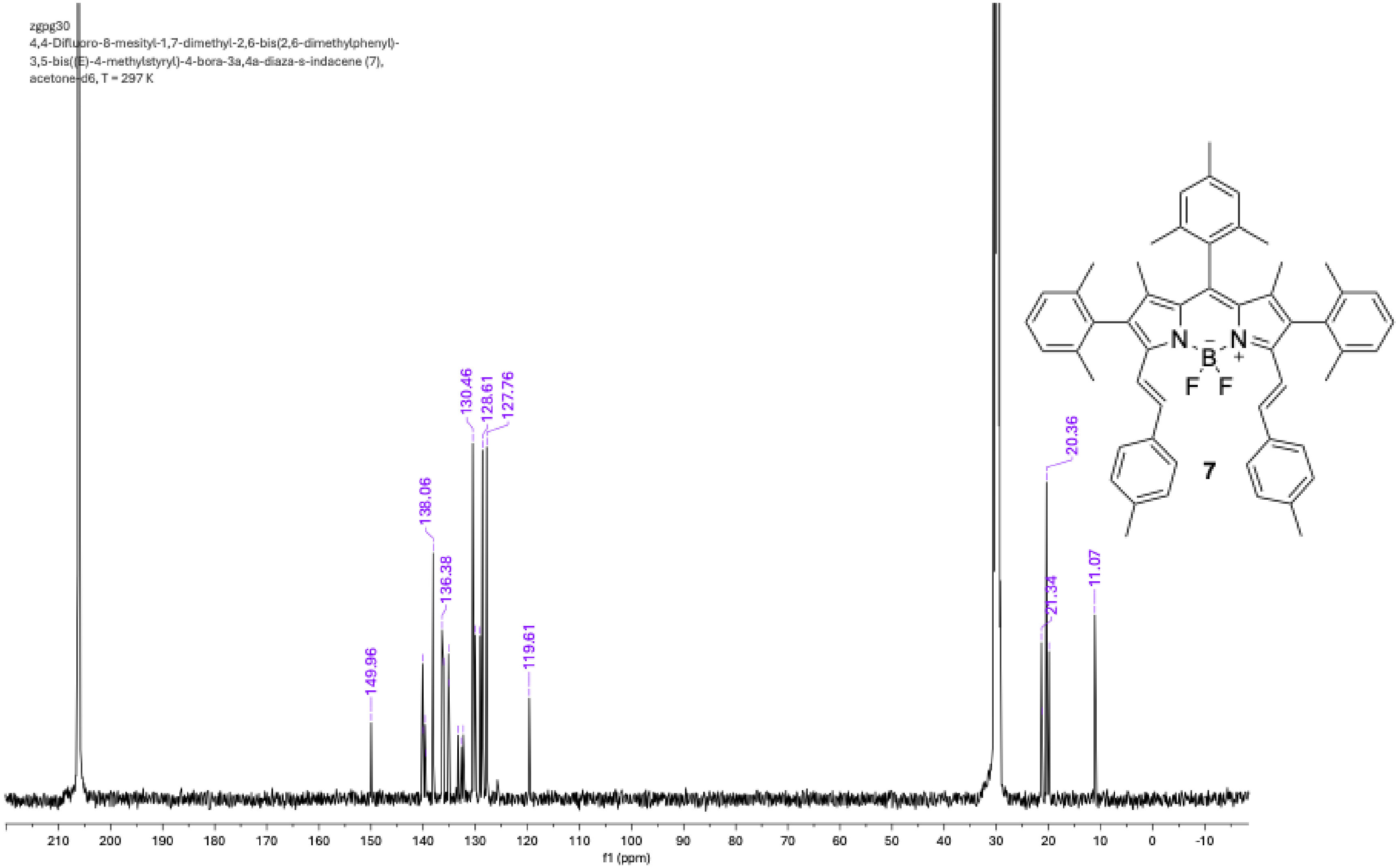

Figure S11. ^{13}C NMR spectrum of 4,4-difluoro-8-mesityl-1,7-dimethyl-2,6-bis(2,6-dimethylphenyl)-3,5-bis((E)-4-methylstyryl)-4-bora-3a,4a-diaza-s-indacene (**7**).

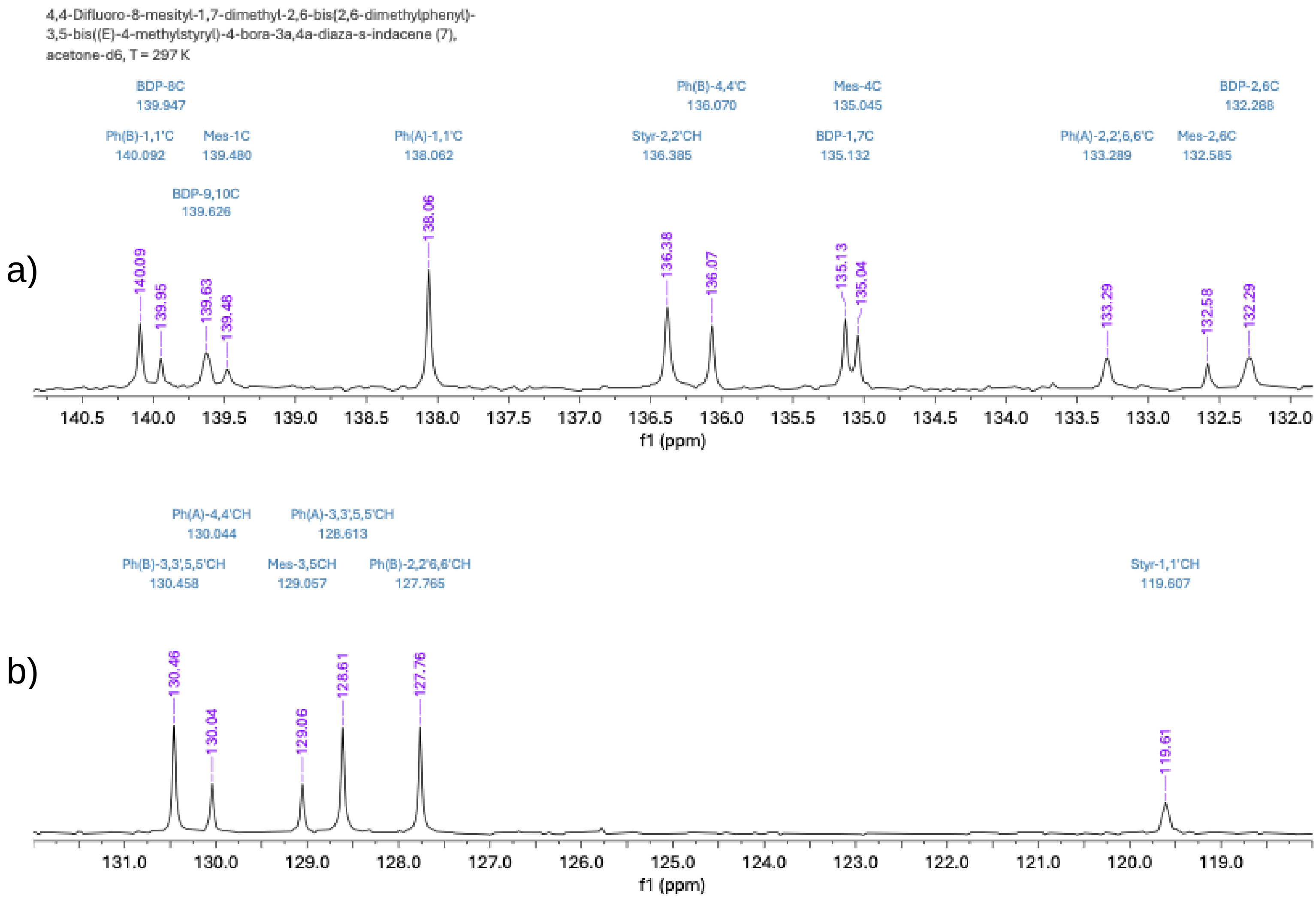

Figure S12. ^{13}C NMR spectrum of 4,4-difluoro-8-mesityl-1,7-dimethyl-2,6-bis(2,6-dimethylphenyl)-3,5-bis((E)-4-methylstyryl)-4-bora-3a,4a-diaza-s-indacene (**7**): a) SW = 141.0 – 135.0 ppm; b) SW = 132.0 – 118.0 ppm.

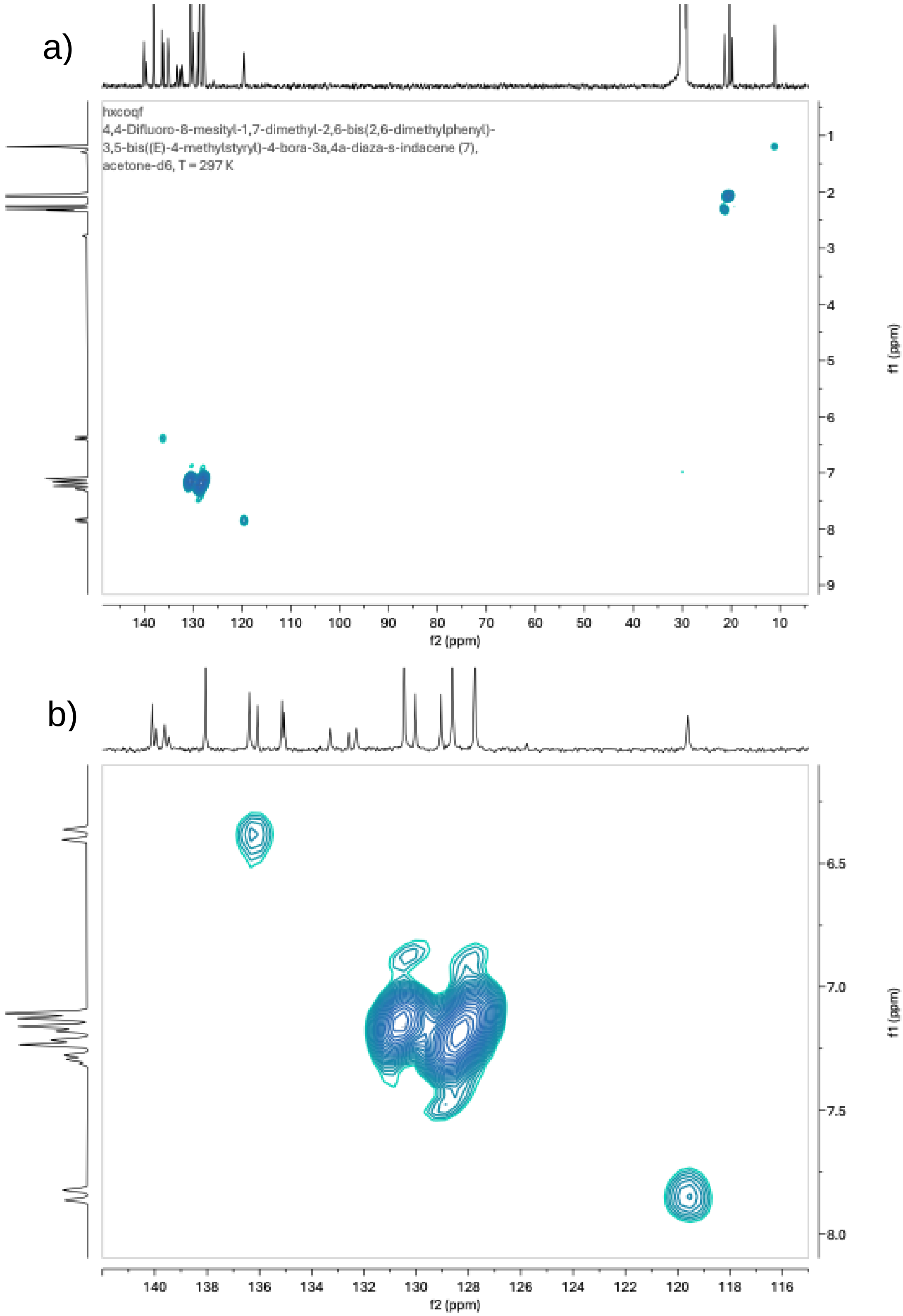

Figure S13. Bidimensional HXCOQF spectra of 4,4-difluoro-8-mesityl-1,7-dimethyl-2,6-bis(2,6-dimethylphenyl)-3,5-bis((E)-4-methylstyryl)-4-bora-3a,4a-diaza-s-indacene (**7**): a) full spectrum; b) SW(f1) = 8.10 – 6.10 ppm, SW(f2) = 142.0 – 115.0 ppm.

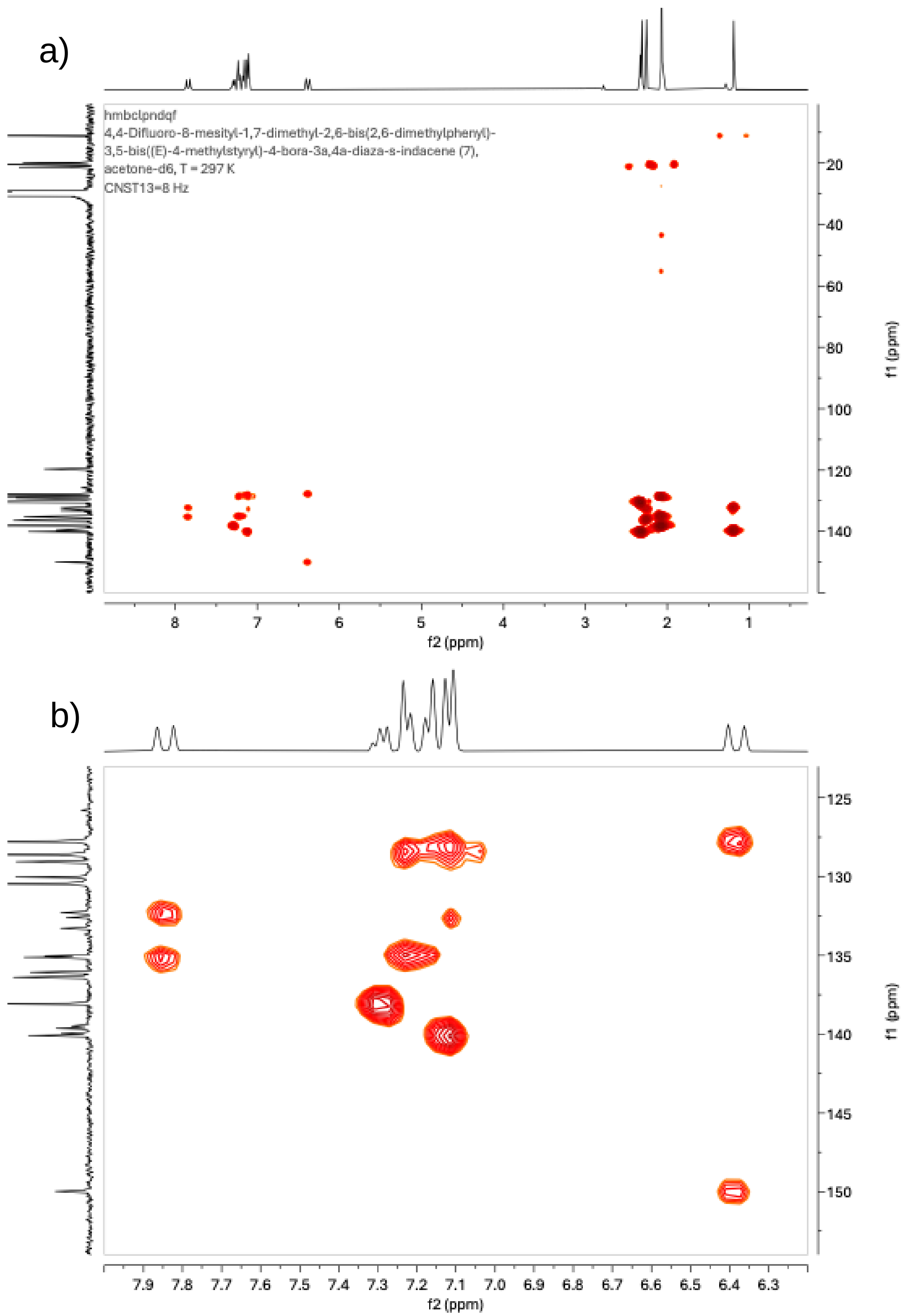

Figure S14. Bidimensional HMBCLPNDQF spectrum of 4,4-difluoro-8-mesityl-1,7-dimethyl-2,6-bis(2,6-dimethylphenyl)-3,5-bis((E)-4-methylstyryl)-4-bora-3a,4a-diaza-s-indacene (**7**): a) full spectrum; b) SW(f1) = 154.0 – 123.0 ppm, SW(f2) = 8.00 – 6.20 ppm.

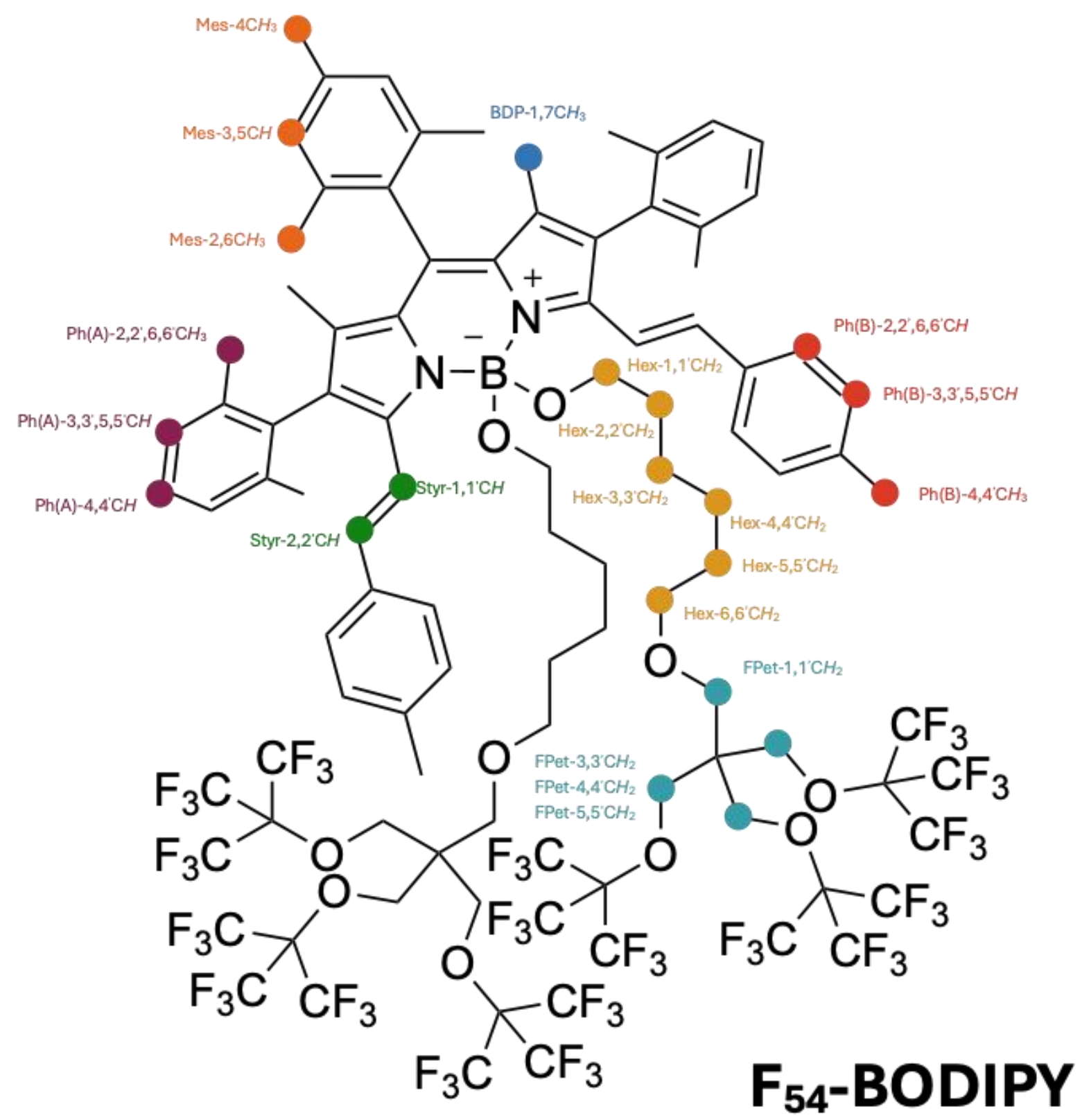

	Assignment	Shift	Range	H's	Integral	Multiplicity	J (Hz)
1	Styr-1,1'*CH*	8.36	8.44 .. 8.29	2	2.00	d	16.81
2	Ph(A)-4,4'*CH*	7.28	7.31 .. 7.25	2	2.03	dd	6.54, 8.46
3	Ph(A)-3,3',5,5'*CH*	7.20	7.24 .. 7.17	4	4.10	d	7.51
4	Ph(B)-2,2',6,6'*CH*; Ph(B)-3,3',5,5'*CH*	7.12	7.14 .. 7.10	8	7.93	s	
5	Mes-3,5*CH*	7.09	7.10 .. 7.08	2	2.09	s	
6	Styr-2,2'*CH*	6.25	6.33 .. 6.14	2	2.01	d	16.81
7	FPET-3,3',4,4',5,5'CH_2	4.14	4.18 .. 4.09	12	12.40	s	
8	FPET-1,1'CH_2; Hex-1,1'CH_2	3.28	3.33 .. 3.24	8	8.11	m	
9	Hex-6,6'CH_2	3.20	3.24 .. 3.16	4	4.20	t	6.59
10	Mes-4CH_3	2.33	2.36 .. 2.31	3	3.11	s	
11	Ph(B)-4,4'CH_3	2.29	2.31 .. 2.28	6	5.82	s	
12	Mes-2,6CH_3	2.26	2.28 .. 2.24	6	6.45	s	
13	Ph(A)-2,2',6,6'CH_3	2.08	2.11 .. 2.07	12	11.70	s	

14	Hex-5,5'CH_2	1.42	1.47 .. 1.38	4	4.66	m	
15	Hex-2,2'CH_2; Hex-3,3'CH_2	1.33	1.37 .. 1.27	8	8.67	m	
16	BDP-1,7CH_3; Hex-4,4'CH_2	1.16	1.24 .. 1.13	10	10.35	m	

Figure S15. Molecular structure and table of ^{1}H NMR assignments of compound **F_{54}-BODIPY**.

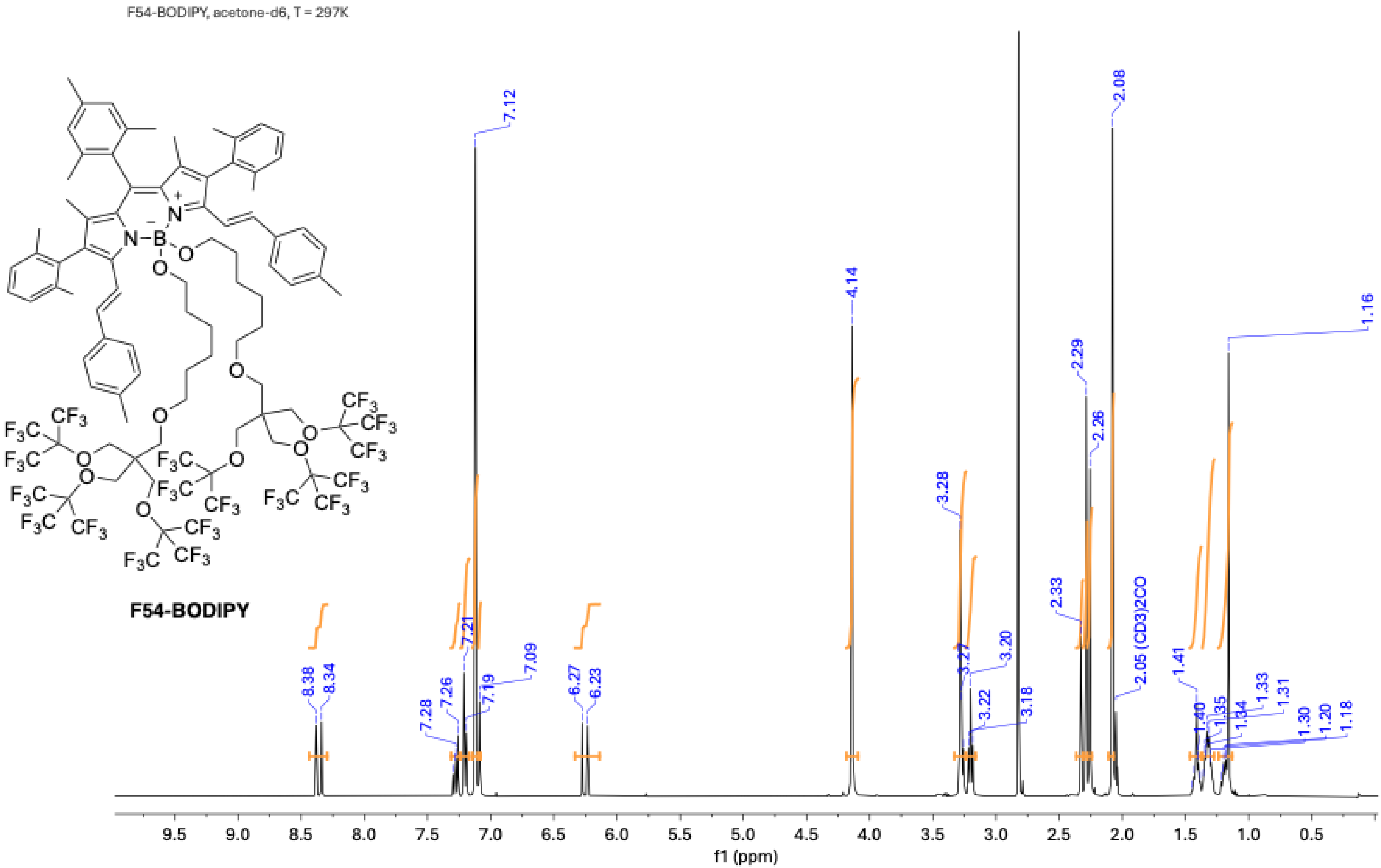

Figure S16. ^{1}H NMR spectrum of compound **F_{54}-BODIPY**.

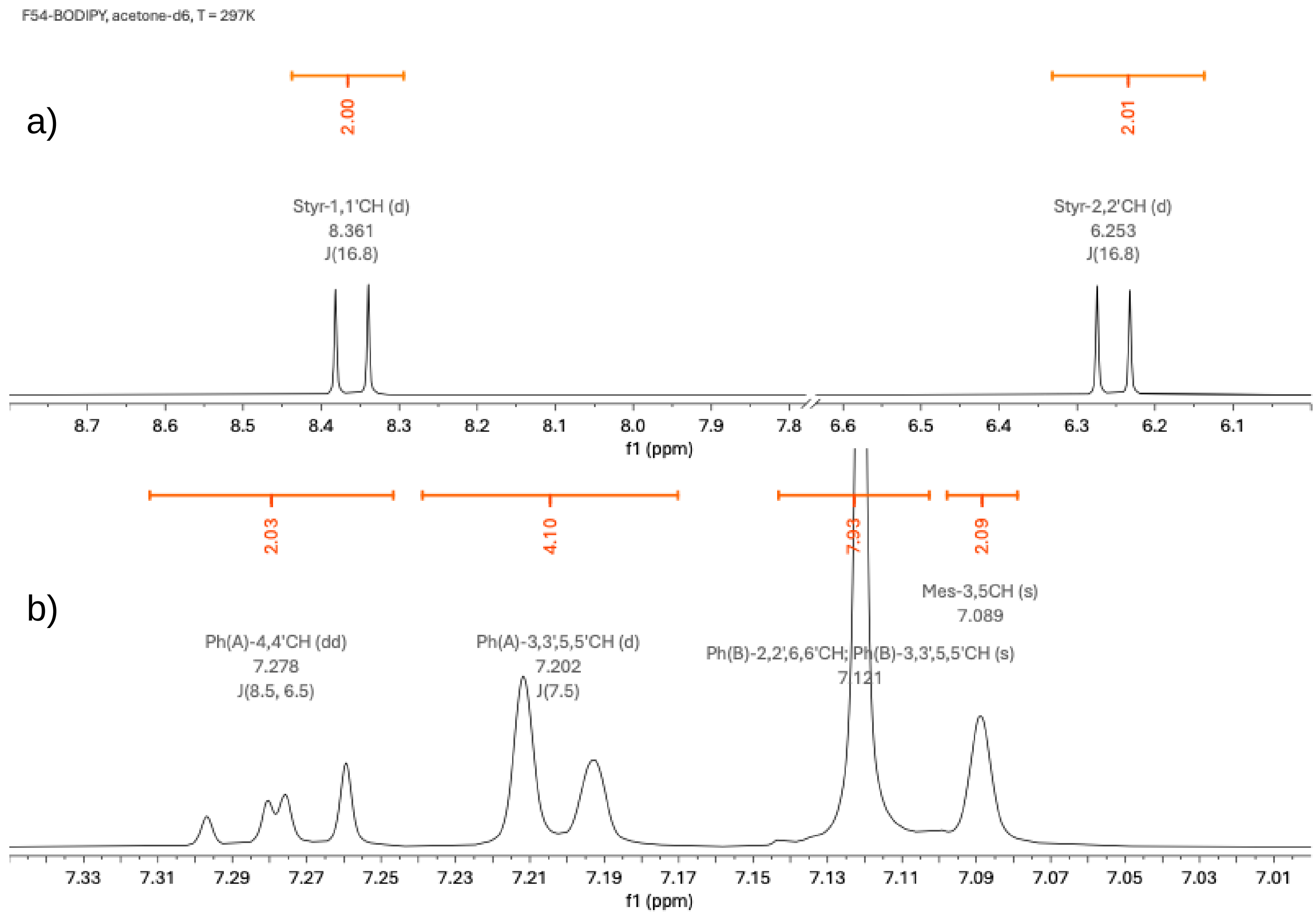

Figure S17. ^{1}H NMR spectrum of compound **F_{54}-BODIPY**: a) SW = 8.80 – 6.00 ppm; b) SW = 7.35 – 7.00 ppm.

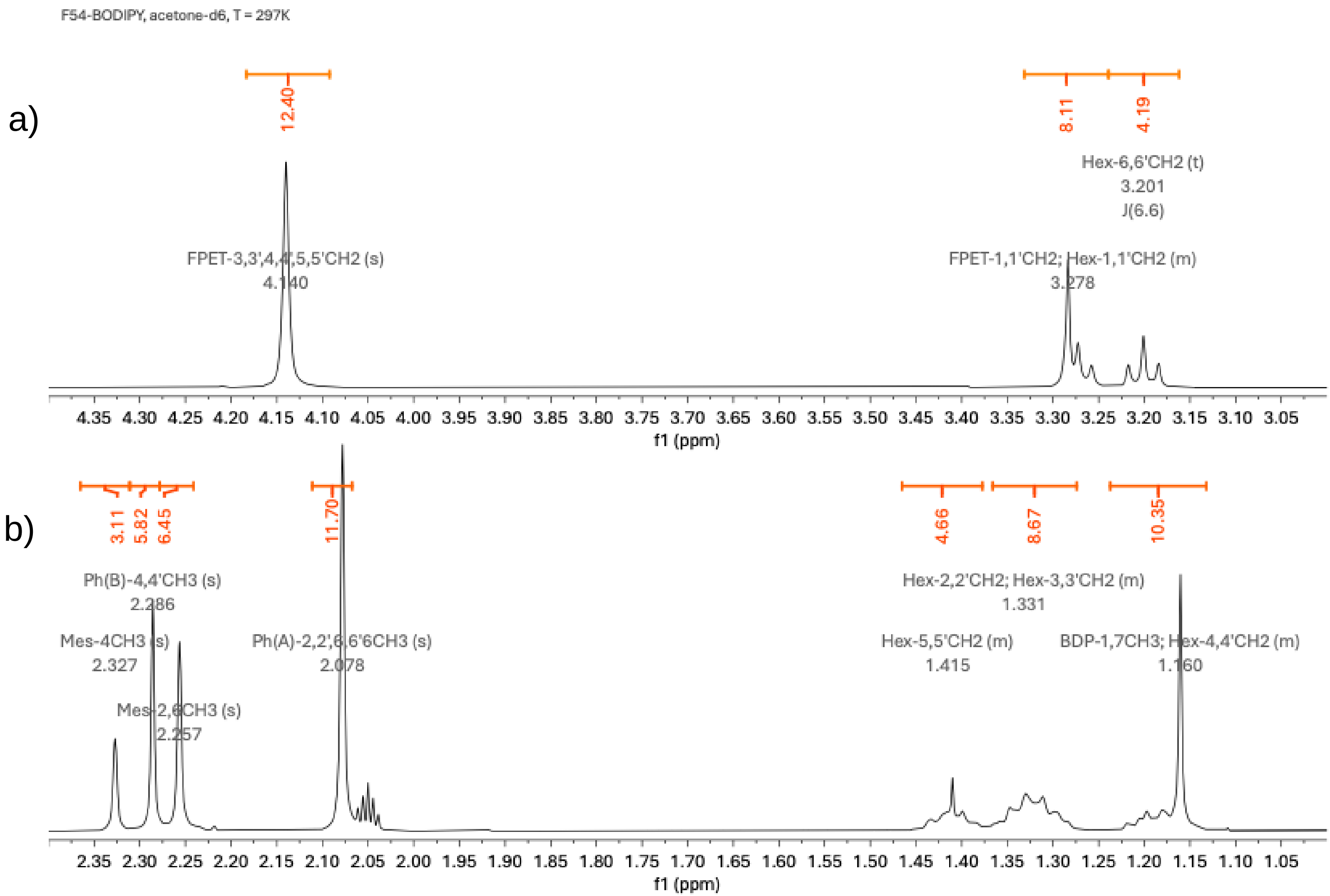

Figure S18. ^{1}H NMR spectrum of compound **F_{54}-BODIPY**: a) SW = 4.40 – 3.00 ppm; b) SW = 2.40 – 1.00 ppm.

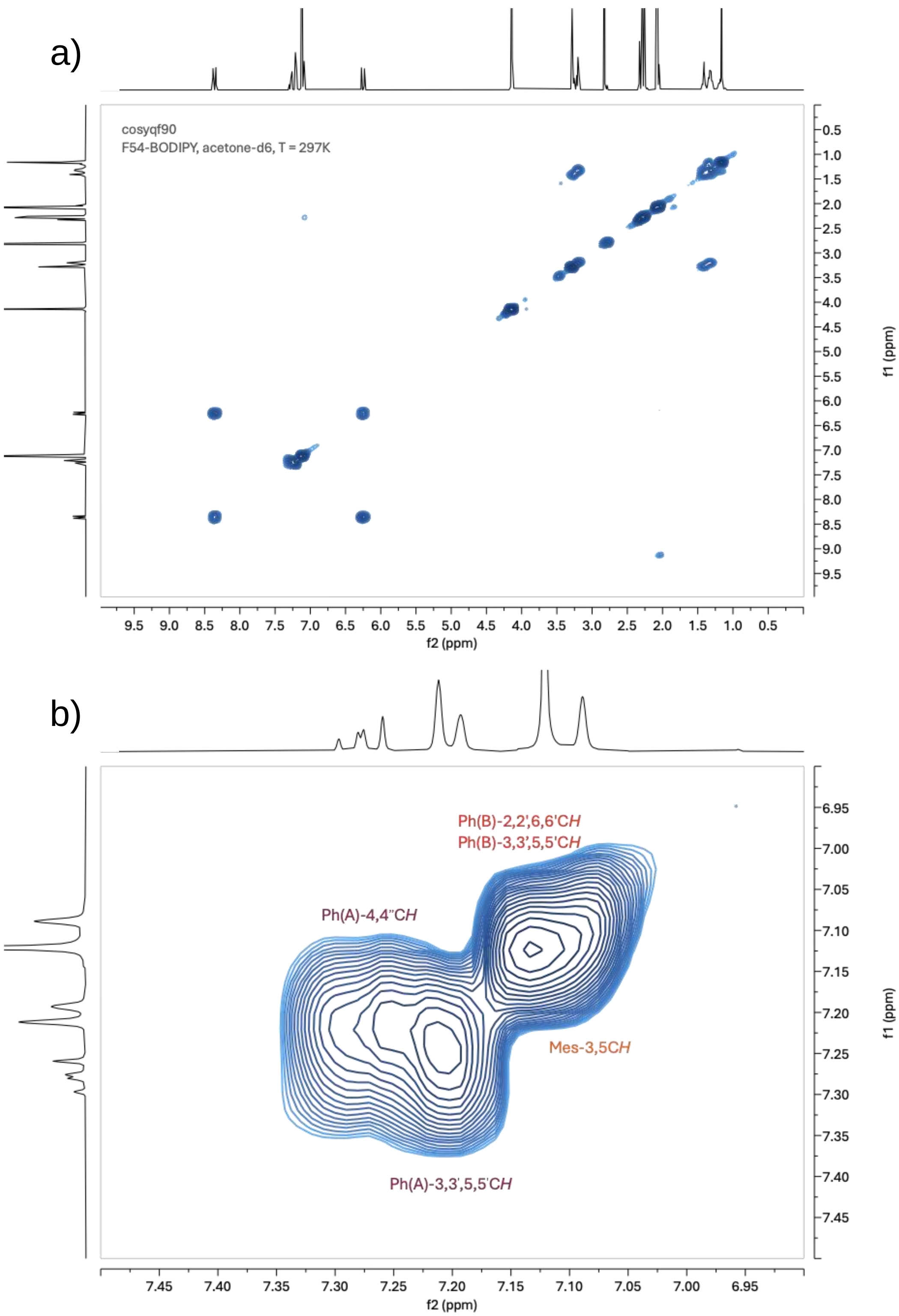

Figure S19. Bidimensional COSYQF90 of compound **F_{54}-BODIPY**: a) full spectrum; b) SW(f1,f2) = 7.50 – 6.90 ppm.

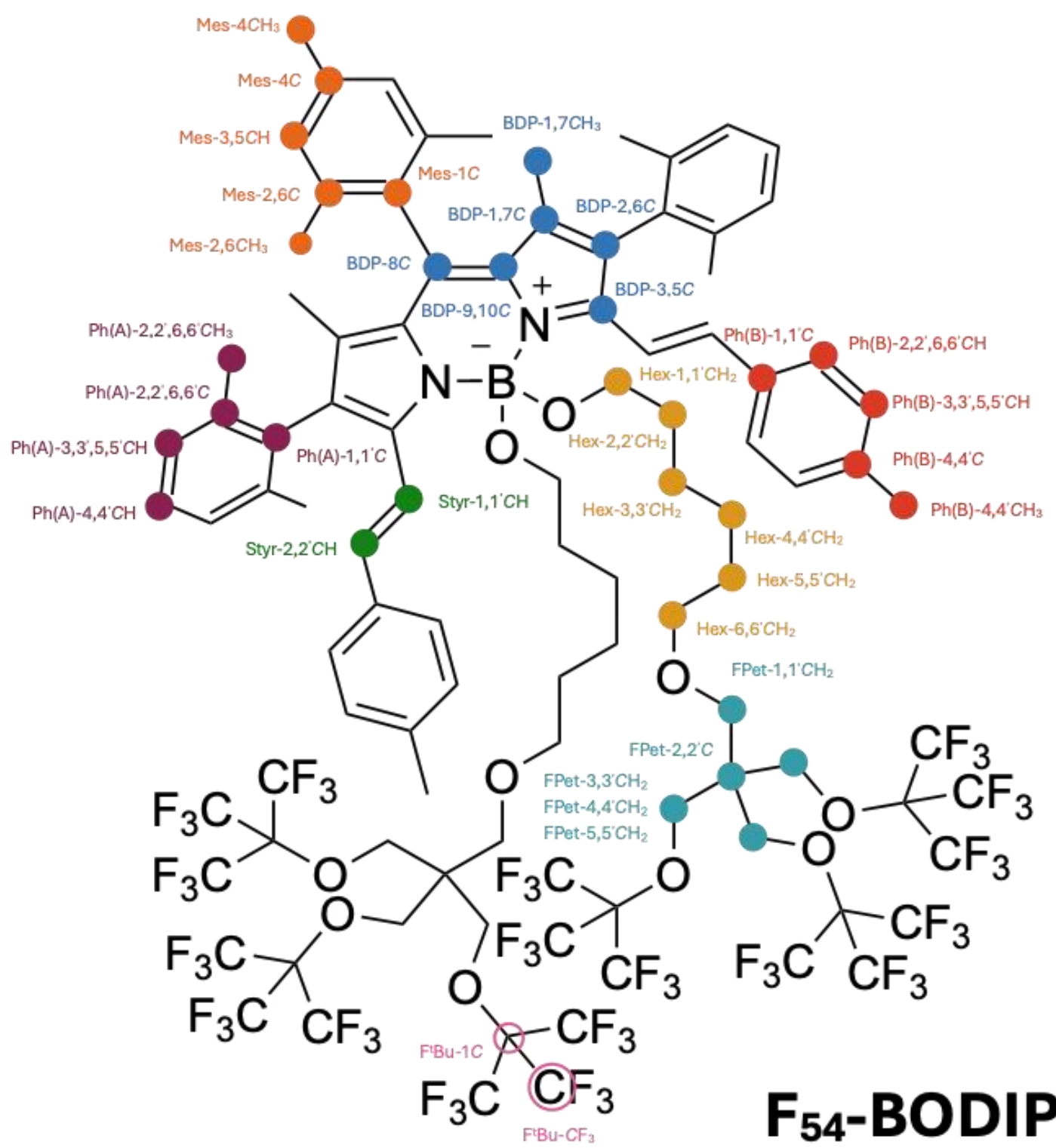

F_{54}-BODIPY

	Assignment	Shift
1	BDP-3,5*C*	149.93
2	BDP-8*C*	139.61
3	Mes-1*C*	139.21
4	Ph(B)-4,4'*C*	139.07
5	Ph(A)-1,1'*C*	137.97
6	BDP-9,10*C*	137.59
7	Mes-2,6*C*; Ph(A)-2,2',6,6'*C*; Ph(B)-1,1'*C* (overlapping)	136.03
8	BDP-1,7*C*	134.63
9	Styr-2,2'CH	134.12
10	Mes-4*C*	133.46
11	BDP-2,6*C*	131.77
12	Ph(B)-3,3',5,5'CH	130.16
13	Mes-3,5CH	129.90
14	Ph(A)-4,4'CH	128.76
15	Ph(A)-3,3',5,5'CH	128.46
16	Ph(B)-2,2',6,6'CH	127.70
17	Styr-1,1'CH	122.00
18	FtBu-*C*F$_3$ (q, J = 292.6 Hz)	121.14
19	FtBu-1,1'*C* (m)	80.47
20	Hex-6,6'*C*H$_2$	72.11
21	FPET-3,3',4,4',5,5'*C*H$_2$	66.38
22	FPET-1,1'*C*H$_2$	65.73
23	Hex-1,1'*C*H$_2$	61.98
24	FPET-2,2'*C*	47.19
25	Hex-5,5'*C*H$_2$	33.15
26	Hex-4,4'*C*H$_2$	27.57
27	Hex-2,2'*C*H$_2$ (overlapping with acetone)	30.00
28	Hex-3,3'*C*H$_2$	26.71
29	Mes-4*C*H$_3$; Ph(B)-4,4'*C*H$_3$	21.21
30	Ph(A)-2,2'6,6'*C*H$_3$	20.30
31	Mes-2,6*C*H$_3$	19.53
32	BDP-1,7*C*H$_3$	11.03

Figure S20. Molecular structure and table of ^{13}C NMR tentative assignments **F_{54}-BODIPY**.

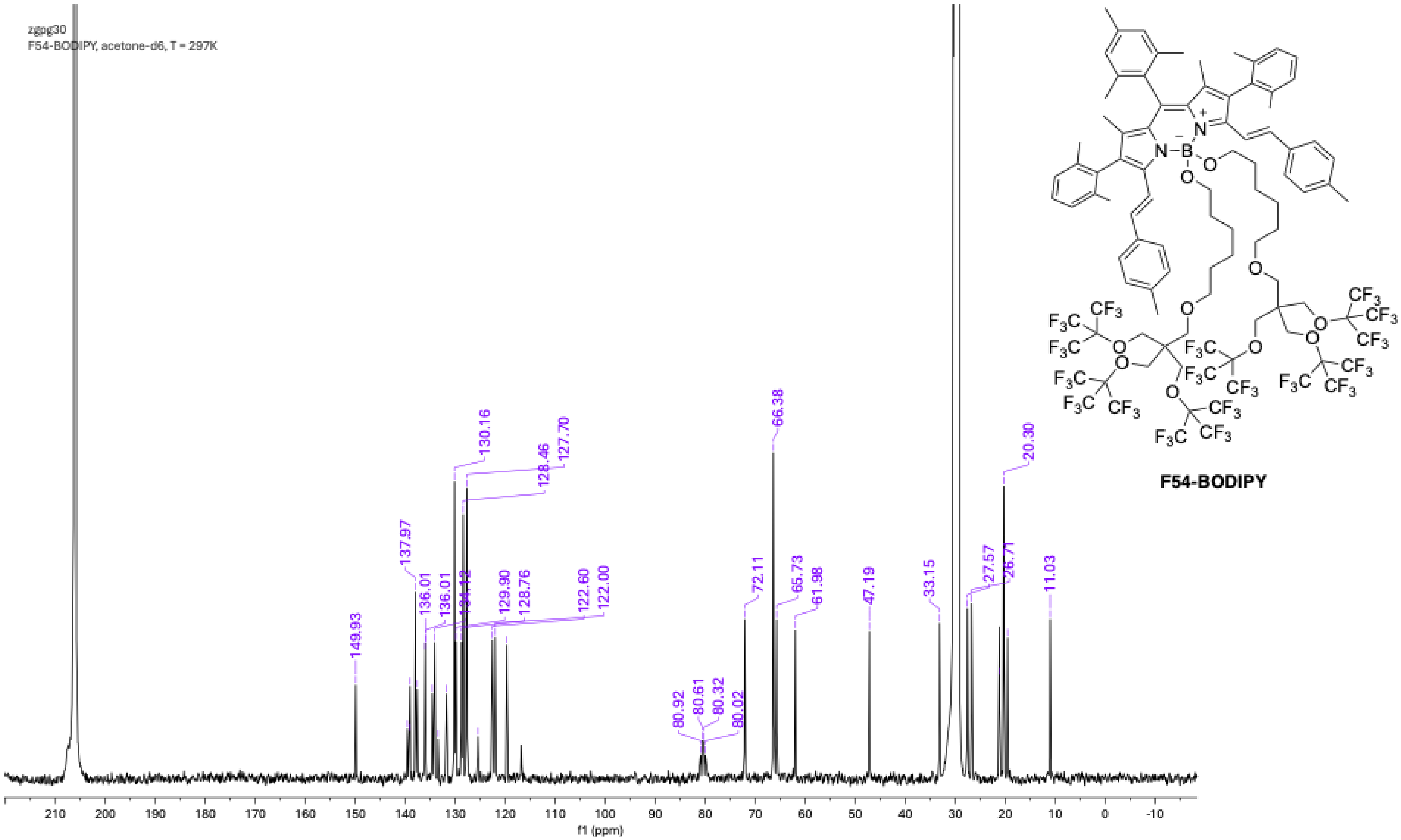

Figure S21. ^{13}C NMR spectrum of **F_{54}-BODIPY**.

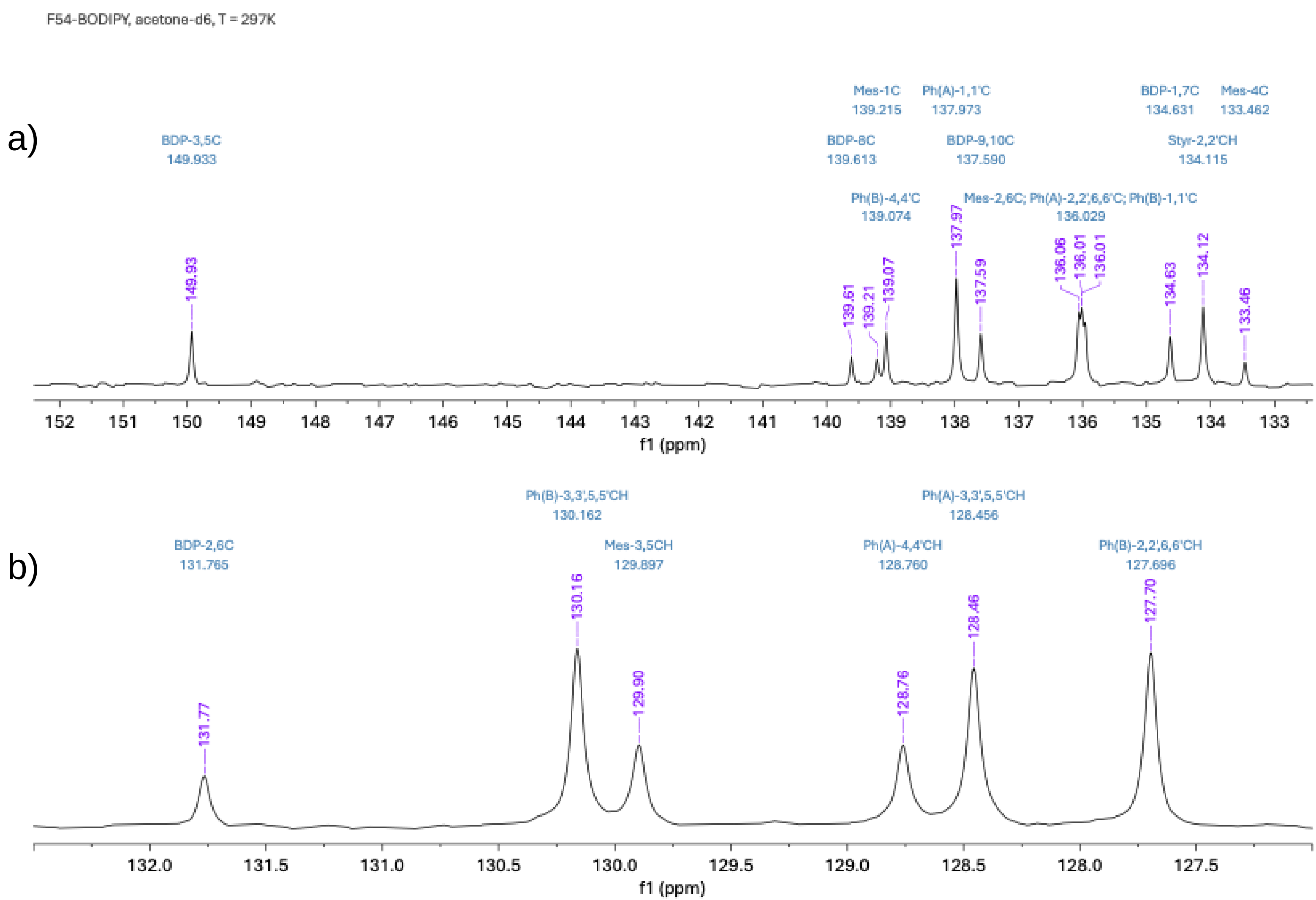

Figure S22. ^{13}C NMR spectrum of **F_{54}-BODIPY**: a) SW = 152.4 – 132.4 ppm; b) SW = 132.5 – 127.0 ppm.

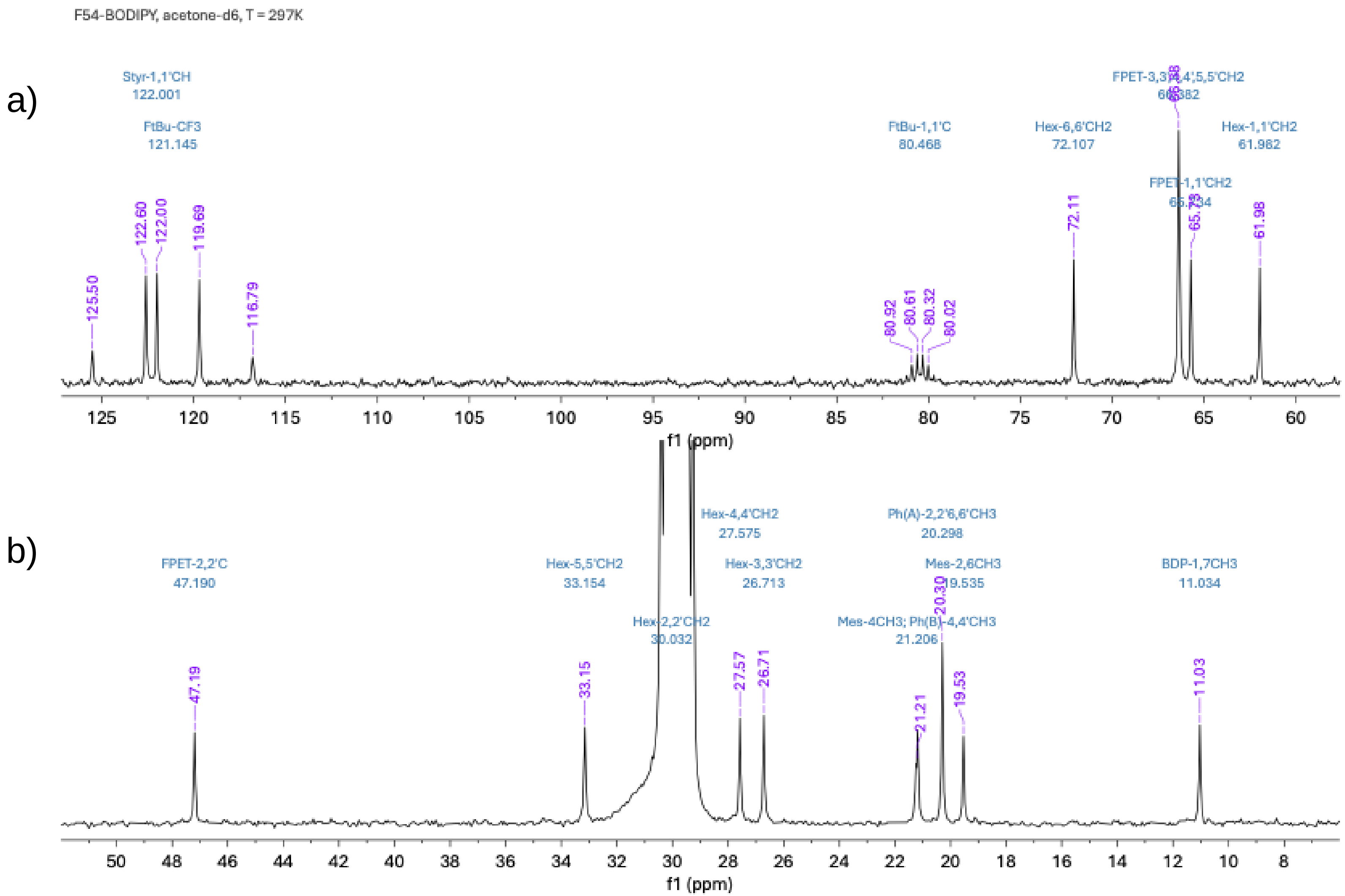

Figure S23. ^{13}C NMR spectrum of **F_{54}-BODIPY**: a) SW = 127.2 – 57.6 ppm; b) SW = 52.0 – 6.0 ppm.

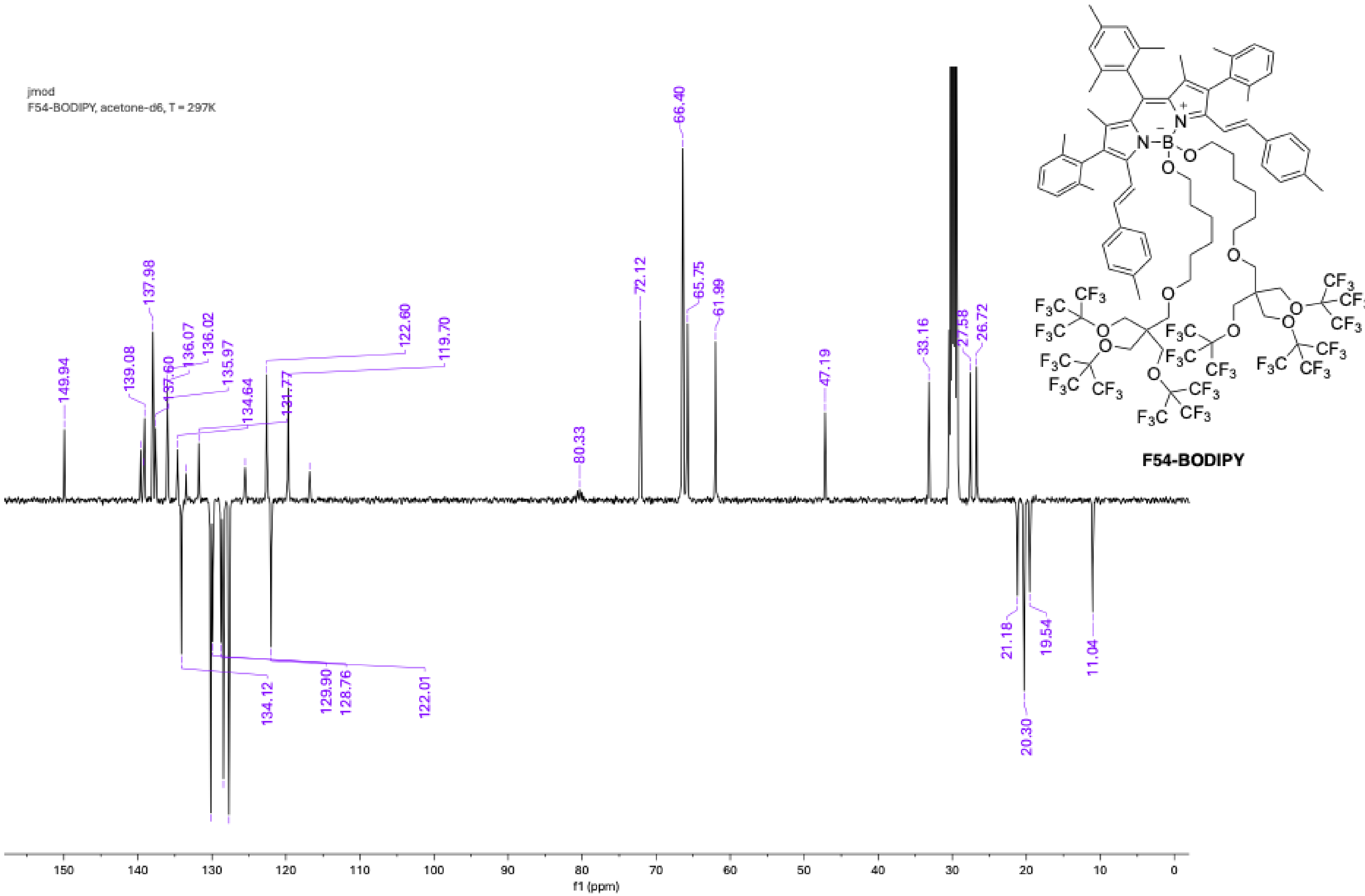

Figure S24. ^{13}C-APT NMR spectrum of **F_{54}-BODIPY.**

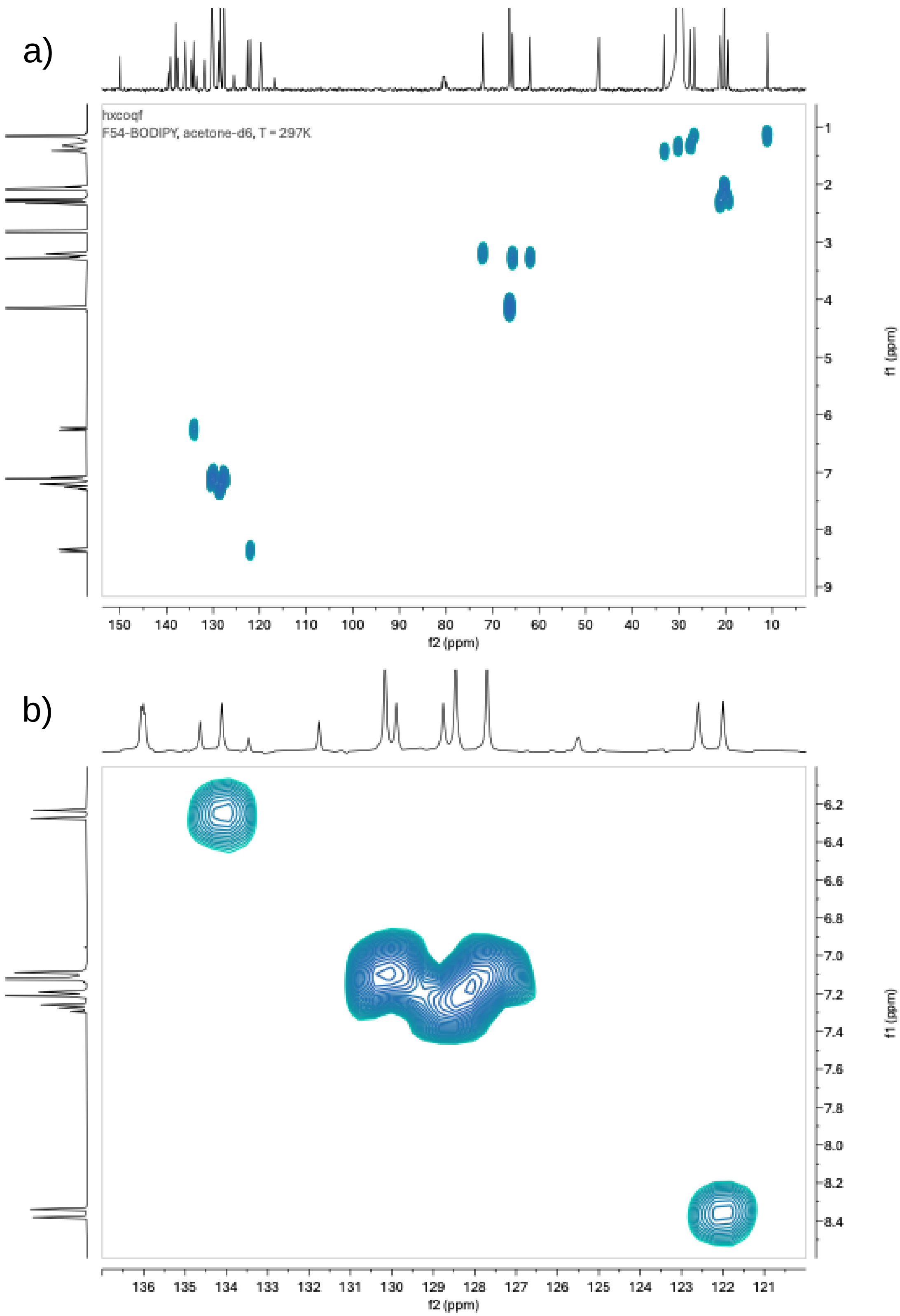

Figure S25. Bidimensional HXCOQF spectra of **F_{54}-BODIPY**: a) full spectrum; b) SW(f1) = 8.60 – 6.00 ppm, SW(f2) = 137.0 – 120.0 ppm.

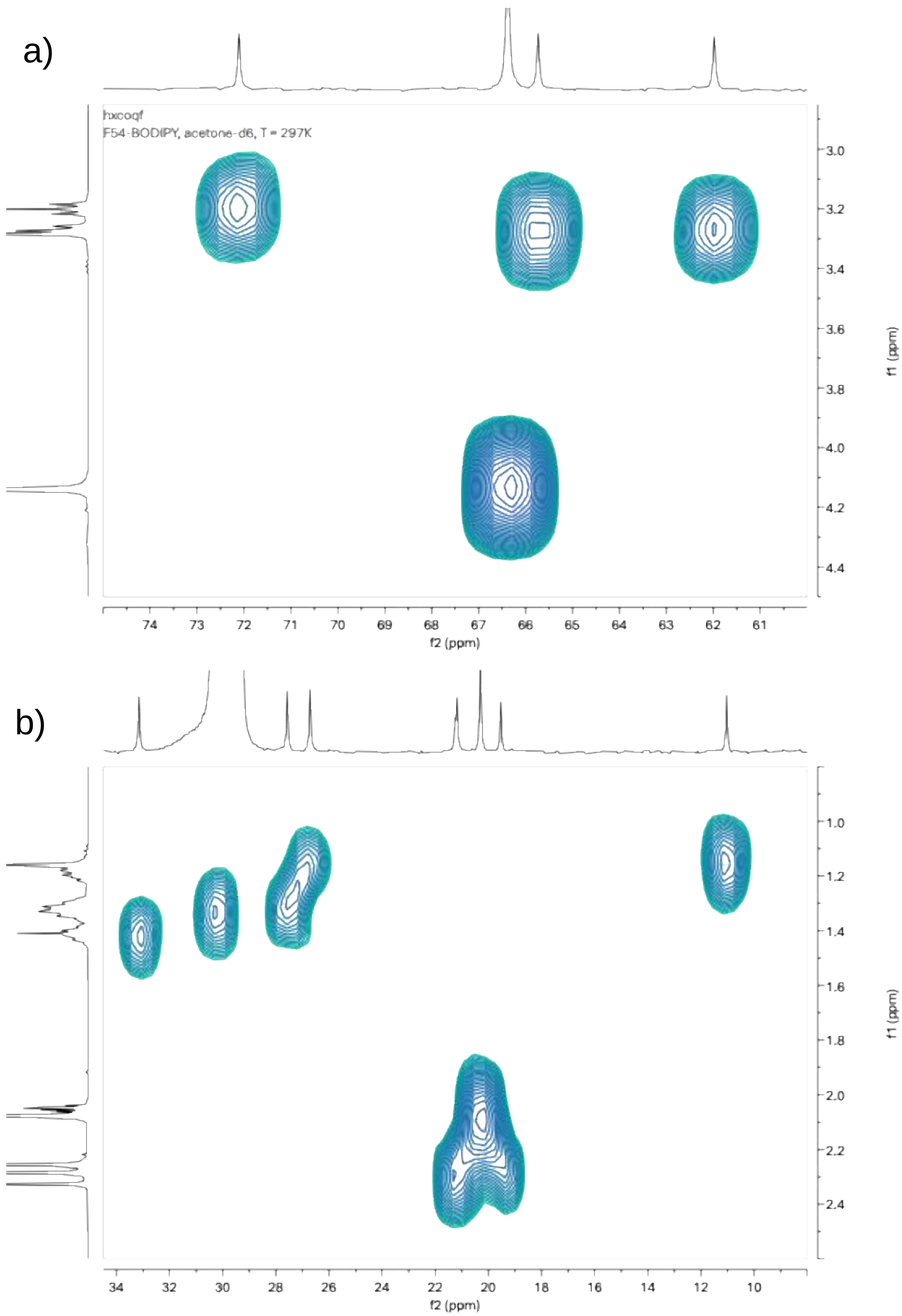

Figure S26. Bidimensional HXCOQF spectra of **F_{54}-BODIPY**. a) SW(f1) = 4.50 – 2.85 ppm, SW(f2) = 75.0 – 60.0 ppm; b) SW(f1) = 2.60 – 0.80 ppm, SW(f2) = 34.5 – 8.0 ppm.

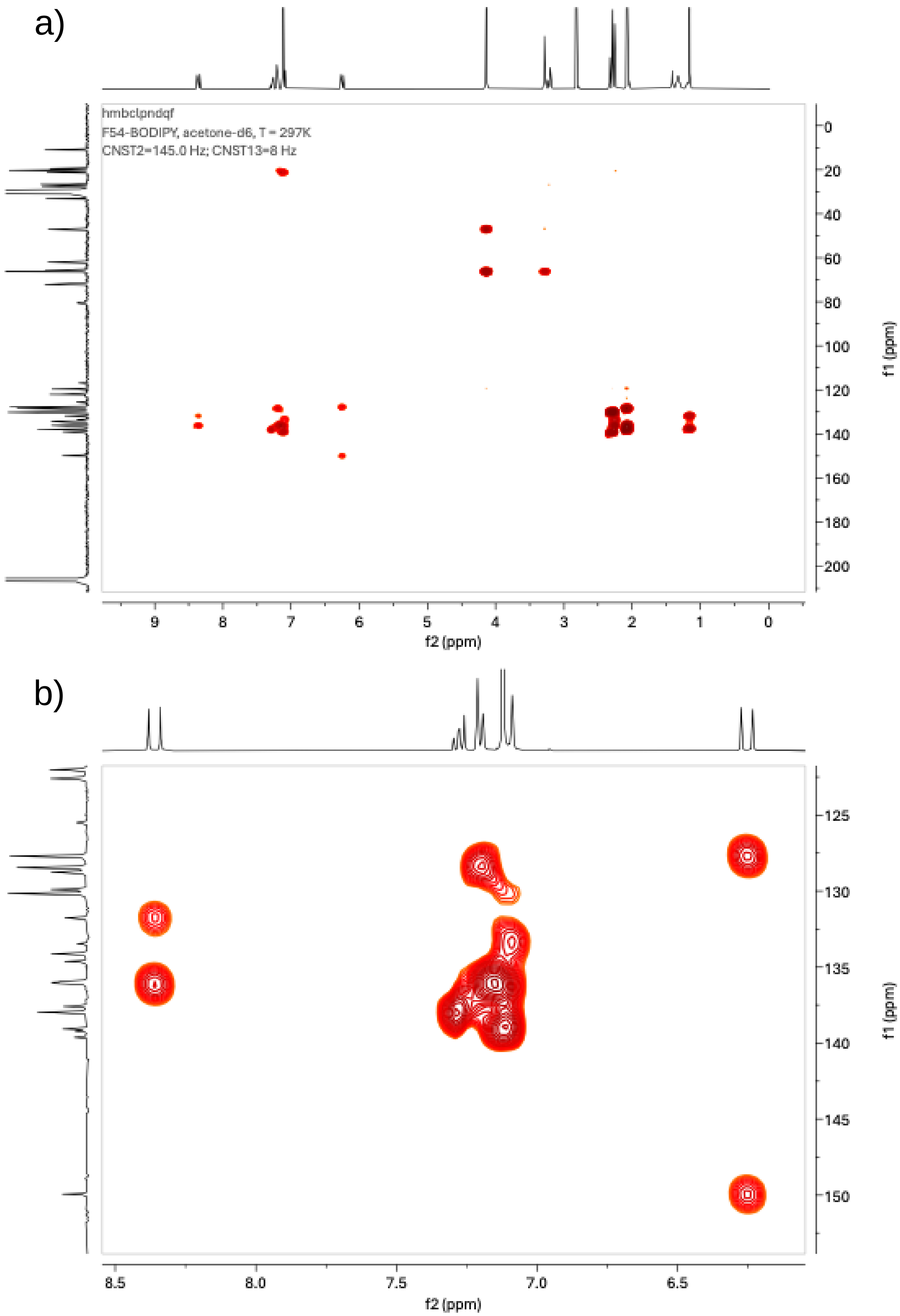

Figure S27. Bidimensional HMBCLPNDQF spectrum of **F_{54}-BODIPY**: a) full spectrum; b) SW(f1) = 154.0 – 123.0 ppm, SW(f2) = 8.60 – 6.00 ppm.

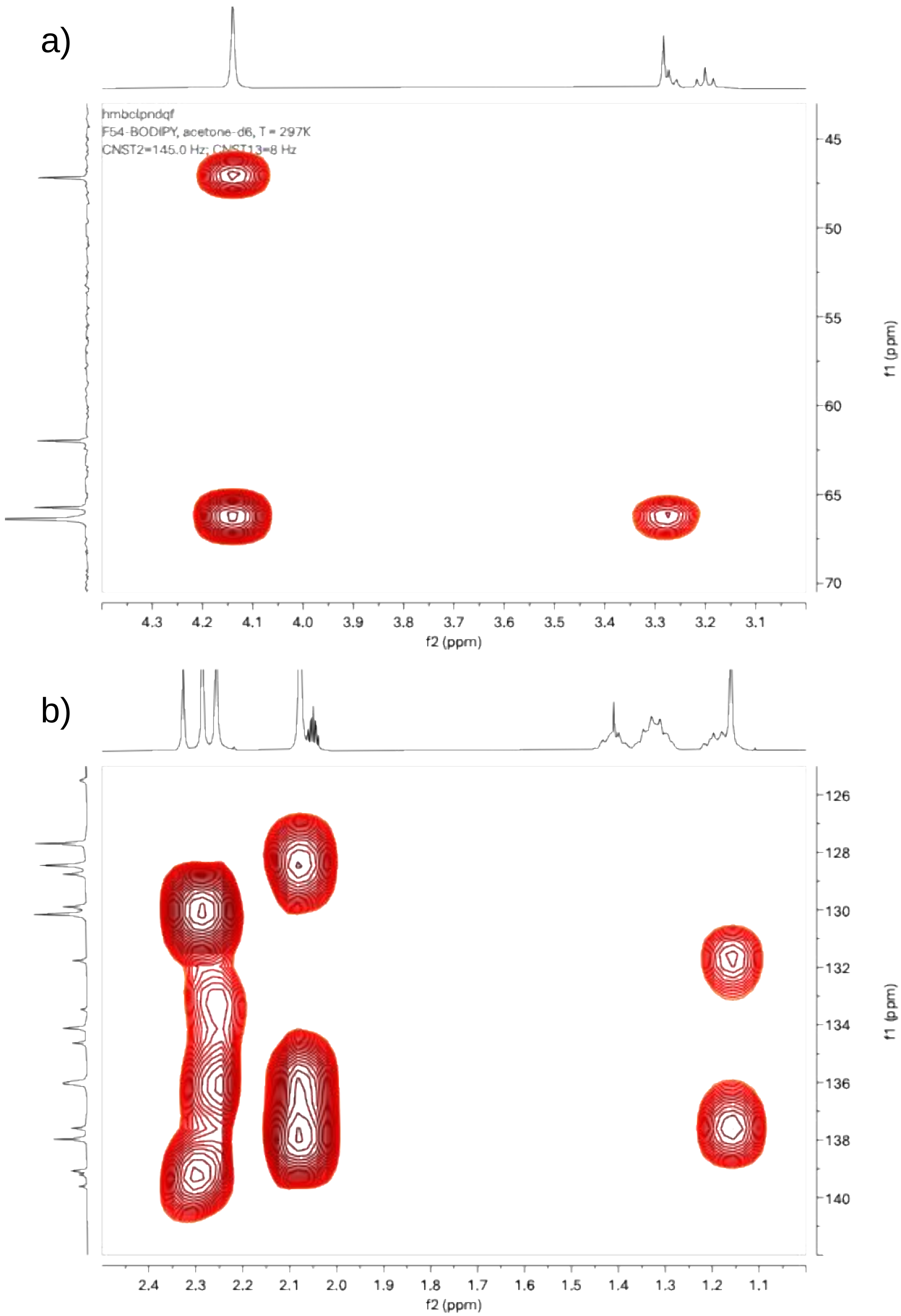

Figure S28. Bidimensional HMBCLPNDQF spectrum of **F_{54}-BODIPY**: a) SW(f1) = 70.5 – 43.0 ppm, SW(f2) = 4.40 – 3.00 ppm; b) SW(f1) = 142.0 – 125.0 ppm, SW(f2) = 2.50 – 1.00 ppm.

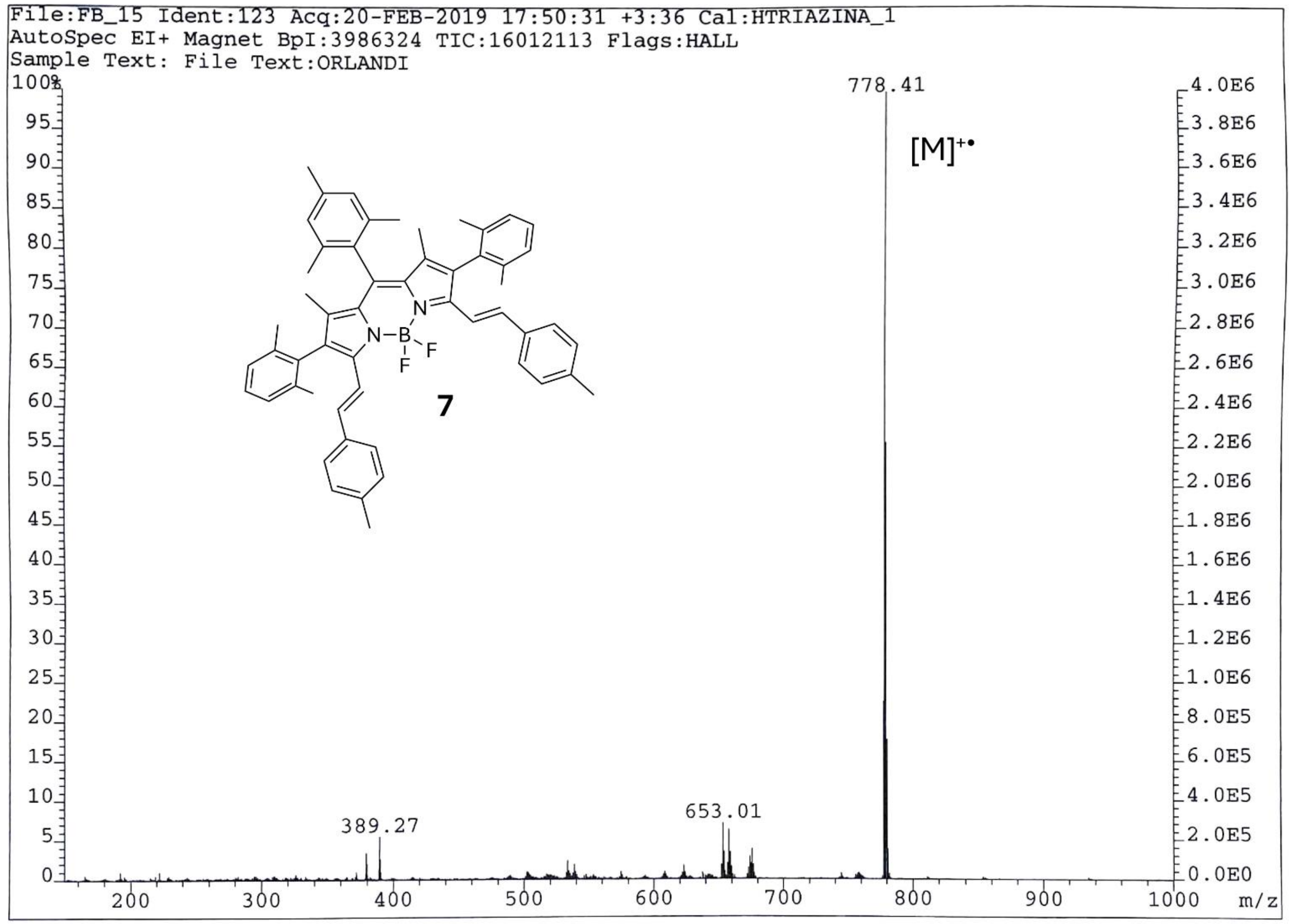

Figure S29. MS-EI+ of 4,4-difluoro-8-mesityl-1,7-dimethyl-2,6-bis(2,6-dimethylphenyl)-3,5-bis((E)-4-methylstyryl)-4-bora-3a,4a-diaza-s-indacene (**7**).

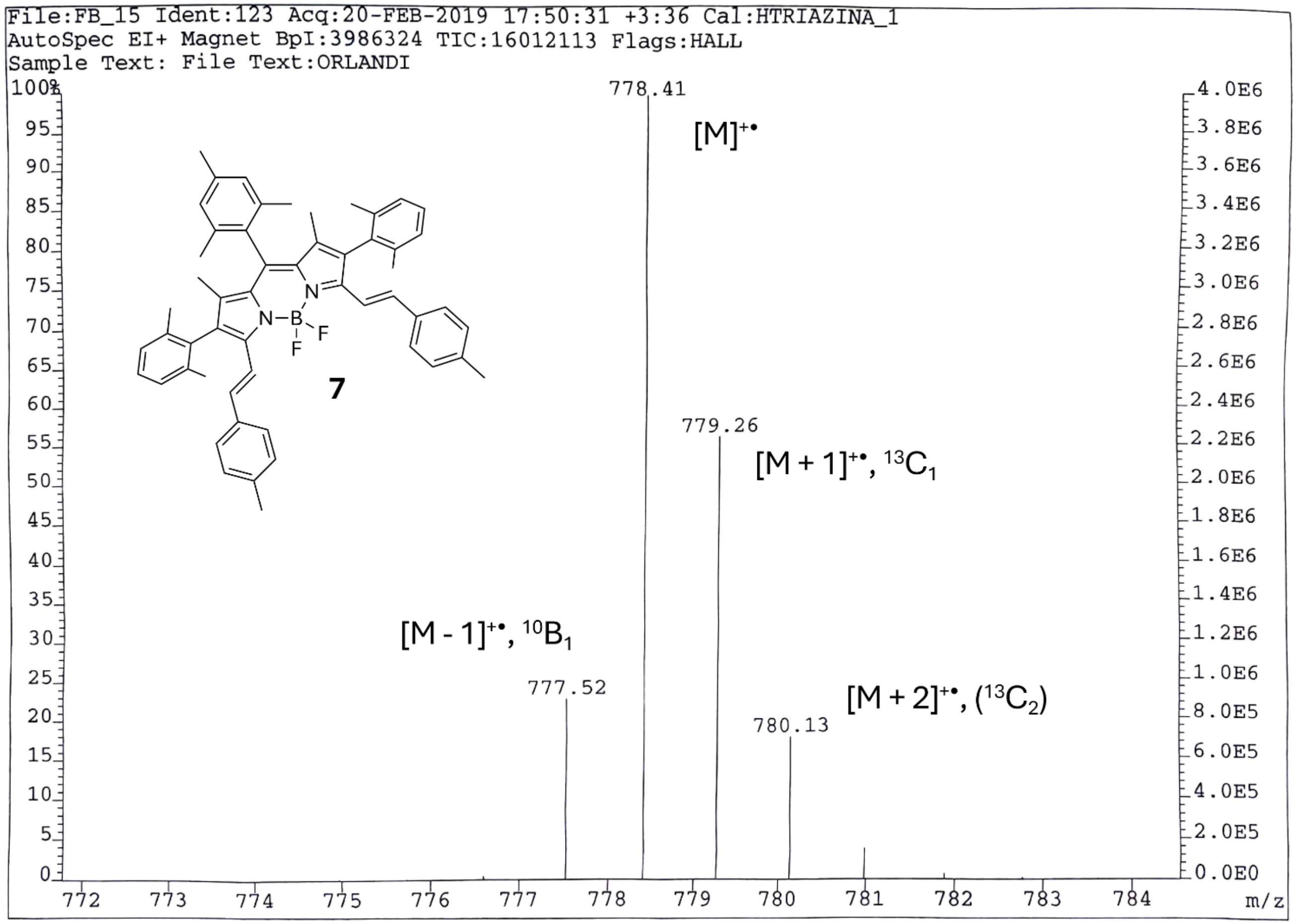

Figure S30. Molecular-ion cluster of 4,4-difluoro-8-mesityl-1,7-dimethyl-2,6-bis(2,6-dimethylphenyl)-3,5-bis((E)-4-methylstyryl)-4-bora-3a,4a-diaza-s-indacene (**7**) in MS-EI$^+$.

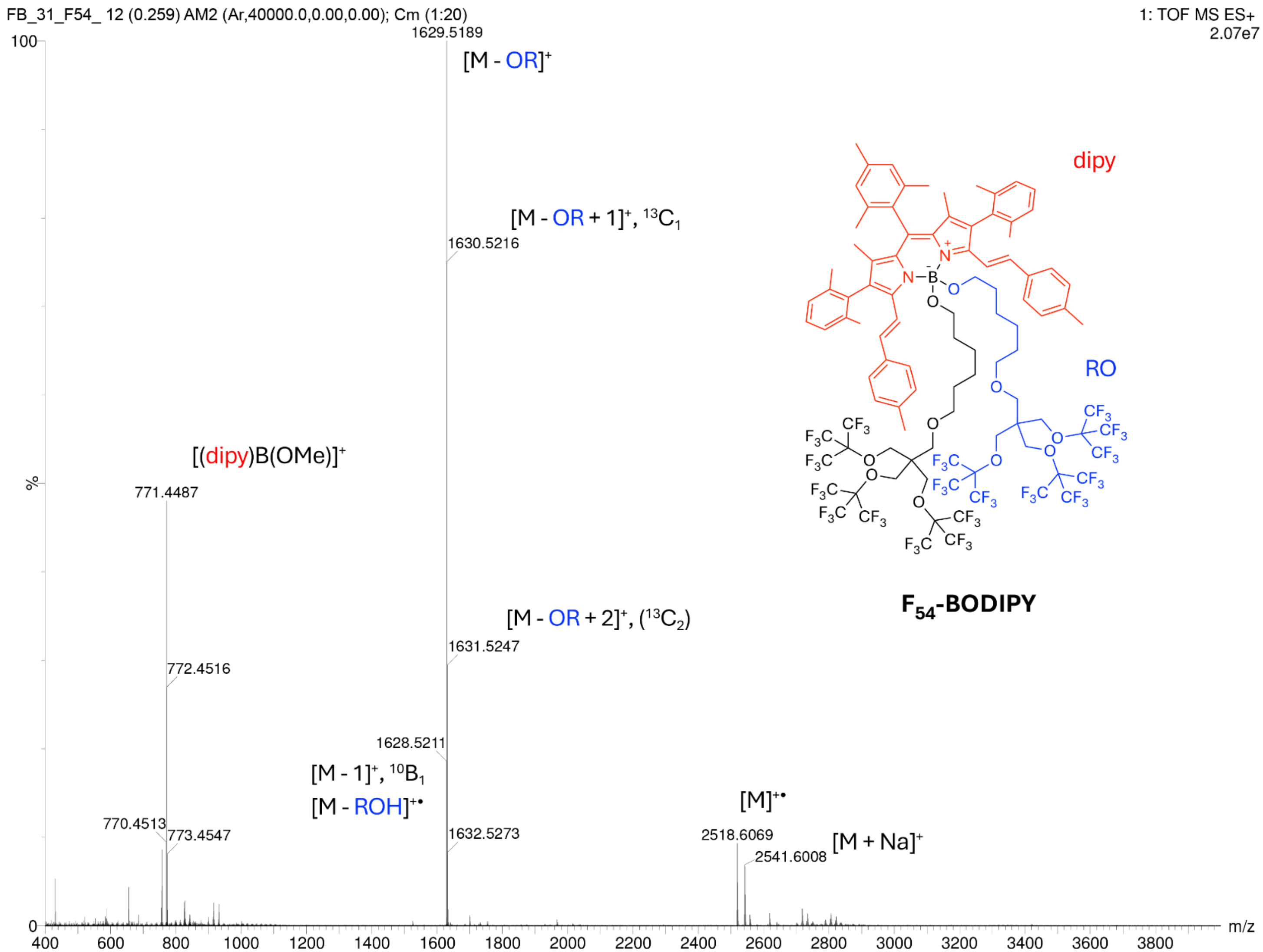

Figure S31. HRMS-ESI+ of compound **F_{54}-BODIPY**.

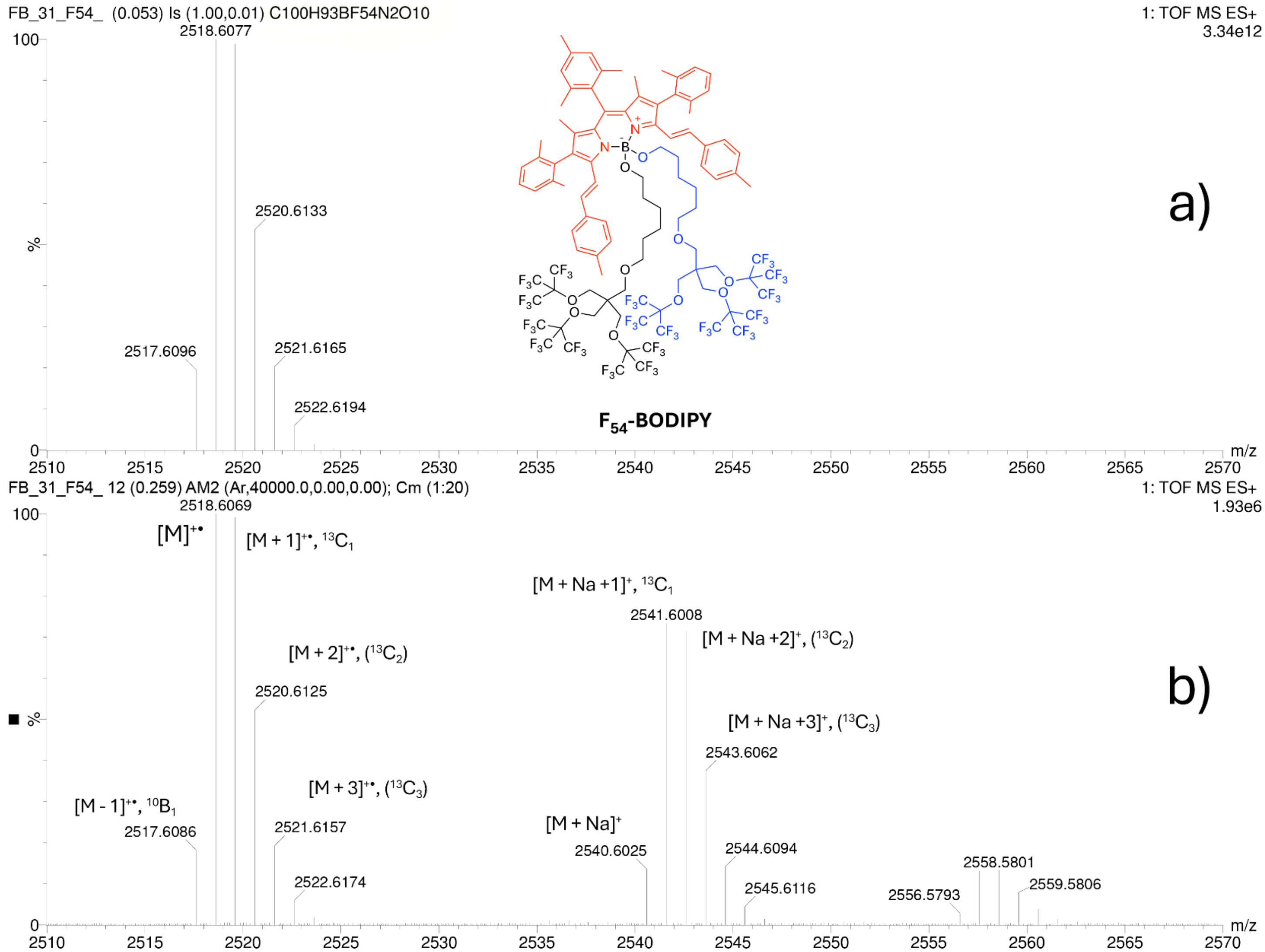

Figure S32. Molecular-ion cluster of compound **F_{54}-BODIPY** in HRMS-ESI$^+$: a) simulated, b) experimental.

References

(1) Sheldrick, G. A short history of SHELX. *Acta Crystallographica Section A* **2008**, *64* (1), 112-122. DOI: doi:10.1107/S0108767307043930.

(2) Sheldrick, G. Crystal structure refinement with SHELXL. *Acta Crystallographica Section C* **2015**, *71* (1), 3-8. DOI: doi:10.1107/S2053229614024218.

(3) Macrae, C. F.; Sovago, I.; Cottrell, S. J.; Galek, P. T. A.; McCabe, P.; Pidcock, E.; Platings, M.; Shields, G. P.; Stevens, J. S.; Towler, M.; et al. Mercury 4.0: from visualization to analysis, design and prediction. *Journal of Applied Crystallography* **2020**, *53* (1), 226-235. DOI: doi:10.1107/S1600576719014092.

(4) Decato, S.; Bemis, T.; Madsen, E.; Mecozzi, S. Synthesis and characterization of perfluoro-tert-butyl semifluorinated amphiphilic polymers and their potential application in hydrophobic drug delivery. *Polymer Chemistry* **2014**, *5* (22), 6461-6471, 10.1039/C4PY00882K. DOI: 10.1039/C4PY00882K.

(5) Jiang, Z.-X.; Yu, Y. B. The design and synthesis of highly branched and spherically symmetric fluorinated oils and amphiles. *Tetrahedron* **2007**, *63* (19), 3982-3988. DOI: https://doi.org/10.1016/j.tet.2007.03.004.

(6) Szabó, D.; Mohl, J.; Bálint, A.-M.; Bodor, A.; Rábai, J. Novel generation ponytails in fluorous chemistry: Syntheses of primary, secondary, and tertiary (nonafluoro-tert-butyloxy)ethyl amines. *Journal of Fluorine Chemistry* **2006**, *127* (11), 1496-1504. DOI: https://doi.org/10.1016/j.jfluchem.2006.06.020.
